# Flavasperfurans A and B, Two New Asperfuran Derivatives From the Marine‐Derived Fungus *Aspergillus flavus*

**DOI:** 10.1002/cbdv.71713

**Published:** 2026-09-15

**Authors:** Chun‐Xiu Wang, Zheng‐Biao Zou, Li‐Sheng Li, Cheng‐Hai Gao, Xian‐Wen Yang

**Affiliations:** ^1^ Institute of Marine Drugs Guangxi University of Chinese Medicine Nanning China; ^2^ Hainan Academy of Medical Sciences Hainan Medical University Haikou China; ^3^ School of Basic Medical Sciences Fujian Medical University Fuzhou China

**Keywords:** *Aspergillus flavus*, ferroptosis inhibitory activity, fungus, marine‐derived, secondary metabolites

## Abstract

Marine microorganisms represent a prolific reservoir of chemically diverse natural products endowed with considerable therapeutic potential. In the present study, two new asperfurans (**1** and **2**) and 23 known compounds (**3**–**25**) were obtained from *Aspergillus flavus*, a marine fungus isolated from a water sample of the Western Pacific. Their structures were determined by detailed analysis of the 1D and 2D NMR, HRESIMS, and NMR calculations with DP4+ probability analysis, as well as ECD calculations. Asperfuran (**3**) exhibited potent inhibitory activity against renin‐angiotensin system‐selective lethal 3 (RSL3)‐induced ferroptosis with a half maximal effective concentration (EC_50_) value of 0.81 µM.

## Graphical abstract

Two new asperfurans (**1** and **2**) and 23 known compounds from marine *Aspergillus flavus* were characterized by NMR, HRESIMS, DP4+, and ECD. Asperfuran (**3**) potently inhibited RSL3‐induced ferroptosis (EC_50_ = 0.81 µM).

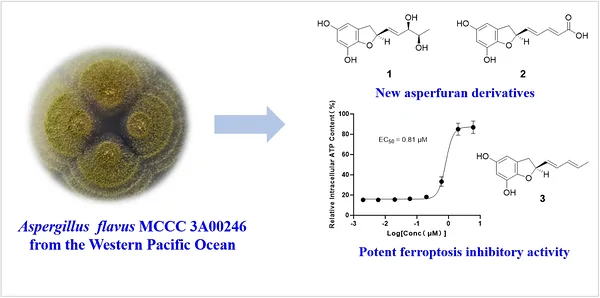

## 1 Introduction

**FIGURE 1:**
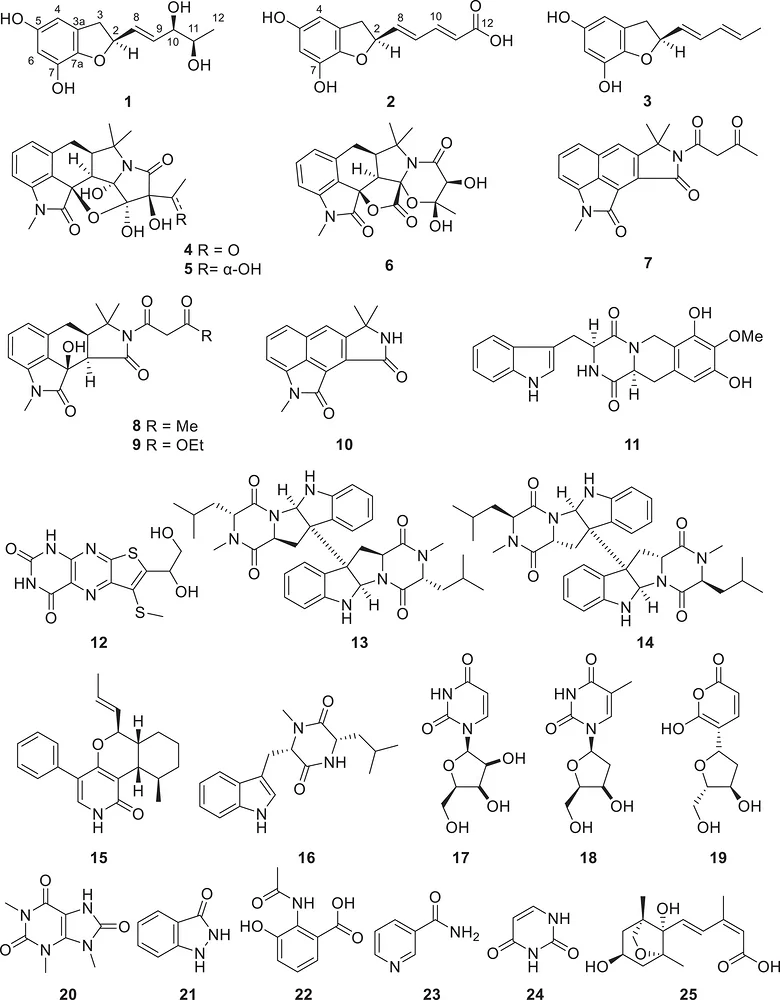
Chemical structures of compounds **1**−**25** of the marine *Aspergillus flavus*.

Marine‐derived fungi have been recognized as an important source of structurally novel and biologically active metabolites [1, 2]. Among them, the genus *Aspergillus* has demonstrated significant potential across agriculture, food, medicine, and environmental protection, emerging as a promising source for the discovery of bioactive compounds [3, 4, 5]. With over 400 species, *Aspergillus* has attracted considerable interest as a rich reservoir of structurally diverse metabolites, including terpenoids, alkaloids, peptides, xanthones, sterols, and polyketides [6, 7, 8, 9, 10, 11]. Notably, these metabolites exhibit a range of bioactivities, such as antiviral, antitumor, antibacterial, anti‐inflammatory, antinecroptosis, antiferroptosis, and enzyme‐inhibitory activities [12, 13, 14]. In our ongoing efforts aimed at the discovery of bioactive metabolites from marine‐derived fungi [15, 16, 17, 18, 19], the fungus *Aspergillus flavus* MCCC 3A00246 was isolated from a water sample collected in the Western Pacific (−75 m). Subsequent chemical investigation of the EtOAc extract of the fermentation broth yielded two new asperfuran derivatives, named flavasperfurans A (**1**) and B (**2**), along with 23 known secondary metabolites (**3**–**25**) (Figure 1). Herein, the detailed isolation, structural elucidation, and biological activities of these compounds are reported.

## 2 Results and Discussion

**FIGURE 2:**
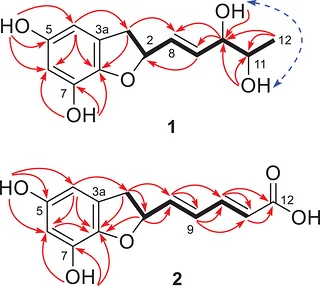
The COSY (bold), HMBC (red arrows), and NOESY (blue arrows) correlations of **1** and **2**.

Compound **1** was isolated as a brown oil. HRESIMS analysis of **1** gave a [M + Na]^+^ peak at *m*/*z* 275.0884 (calcd for 275.0890), indicating the molecular formula C_13_H_16_O_5_, with six degrees of unsaturation. The ^1^H NMR data (Table 1 and Figure S1) showed the presence of four exchangeable protons [*δ*_H_ 8.94 (1H, s, 7‐OH), 8.55 (1H, s, 5‐OH), 4.73 (1H, d, *J* = 5.3 Hz, 10‐OH), 4.42 (1H, d, *J* = 5.3 Hz, 11‐OH)], two aromatic protons [*δ*_H_ 6.05 (1H, d, *J* = 2.4 Hz, H‐4), 6.06 (1H, d, *J* = 2.4 Hz, H‐6)], two olefinic protons [*δ*_H_ 5.82 (1H, dd, *J* = 15.5, 5.3 Hz, H‐9), 5.73 (1H, dd, *J* = 15.7, 6.9 Hz, H‐8)], three oxygenated methines [*δ*_H_ 5.04 (1H, q, *J* = 8.1 Hz, H‐2), 3.71 (1H, m, H‐10), 3.42 (1H, sext, *J* = 5.8 Hz, H‐11)], one methylene [*δ*_H_ 2.75 (1H, dd, *J* = 15.4, 8.3 Hz, H‐3), 3.16 (1H, dd, *J* = 15.7, 9.0 Hz, H‐3)], and one methyl [*δ*_H_ 0.98 (1H, d, *J* = 6.3 Hz, H‐12)]. The ^13^C NMR data of **1** (Table 1 and Figure S2) in conjunction with its HSQC NMR (Figure S3) spectrum provided 13 carbons, containing four sp^2^ quaternary carbons (*δ*_C_ 151.6, 141.1, 139.3, and 127.8), four sp^2^ methines (*δ*_C_ 133.7, 129.5, 102.1, and 102.5), three oxygenated sp^3^ methines (*δ*_C_ 82.4, 74.8, and 69.7), one sp^3^ methylene (*δ*_C_ 36.7), and one methyl (*δ*_C_ 19.1). The ^1^H–^1^H COSY correlations of H_2_‐3/H‐2/H‐8/H‐9/H‐10/H‐11/H_3_‐12 connected a long chain of C‐2/C‐3/C‐8/‐9/C‐10/C‐11/C‐12 (Figures 2 and S4). In the HMBC spectrum (Figures 2 and S5), correlations from H‐3 to C‐7a/C‐8, H‐4 to C‐3/C‐6/C‐7a, 5‐OH to C‐4/C‐5/C‐6, and 7‐OH to C‐6/C‐7/C‐7a established the planar structure of **1** as 2‐(3,4‐dihydroxypent‐1‐en‐1‐yl)‐2,3‐dihydrobenzofuran‐5,7‐diol, a 10,11‐dihydroxylated derivative of the co‐isolated asperfuran (**3**).

**TABLE 1:** ^1^H and ^13^C NMR data of compounds **1** and **2** in DMSO‐*d*_6_.

No.	1		2	
	*δ*_C_, Type	*δ*_H_, mult (*J* in Hz)	*δ*_C_, Type	*δ*_H_, mult (*J* in Hz)
2	82.4 CH	5.04 q (8.1)	81.5 CH	5.19 q (7.7)
3	36.7 CH_2_	2.75 dd (15.4, 8.3)	36.5 CH_2_	2.84 dd (15.7,7.8)
		3.16 dd (15.7, 9.0)		3.27 dd (15.6, 9.2)
3a	127.8 s		127.8 C	
4	102.1 CH	6.05 d (2.4)	102.5 CH	6.07 s
5	151.6 C		152.1 C	
6	102.5 CH	6.06 d (2.4)	102.9 CH	6.07 s
7	141.1 C		141.8 C	
7a	139.3 C		139.4 C	
8	129.5 CH	5.73 dd (15.7, 6.9)	141.4 CH	6.34 dd (15.3, 6.2)
9	133.7 CH	5.82 dd (15.5, 5.3)	128.6 CH	6.47 dd (15.2, 11.0)
10	74.8 CH	3.71 m	143.7 CH	7.19 dd (15.2, 11.4)
11	69.7 CH	3.42 sext (5.8)	123.1 CH	5.91 d (15.3)
12	19.1 CH_3_	0.98 d (6.3)	167.9 C	
5‐OH		8.55 s		8.80 s
7‐OH		8.94 s		9.18 s
10‐OH		4.73 d (5.3)		
11‐OH		4.42 d (5.3)		

**FIGURE 3:**
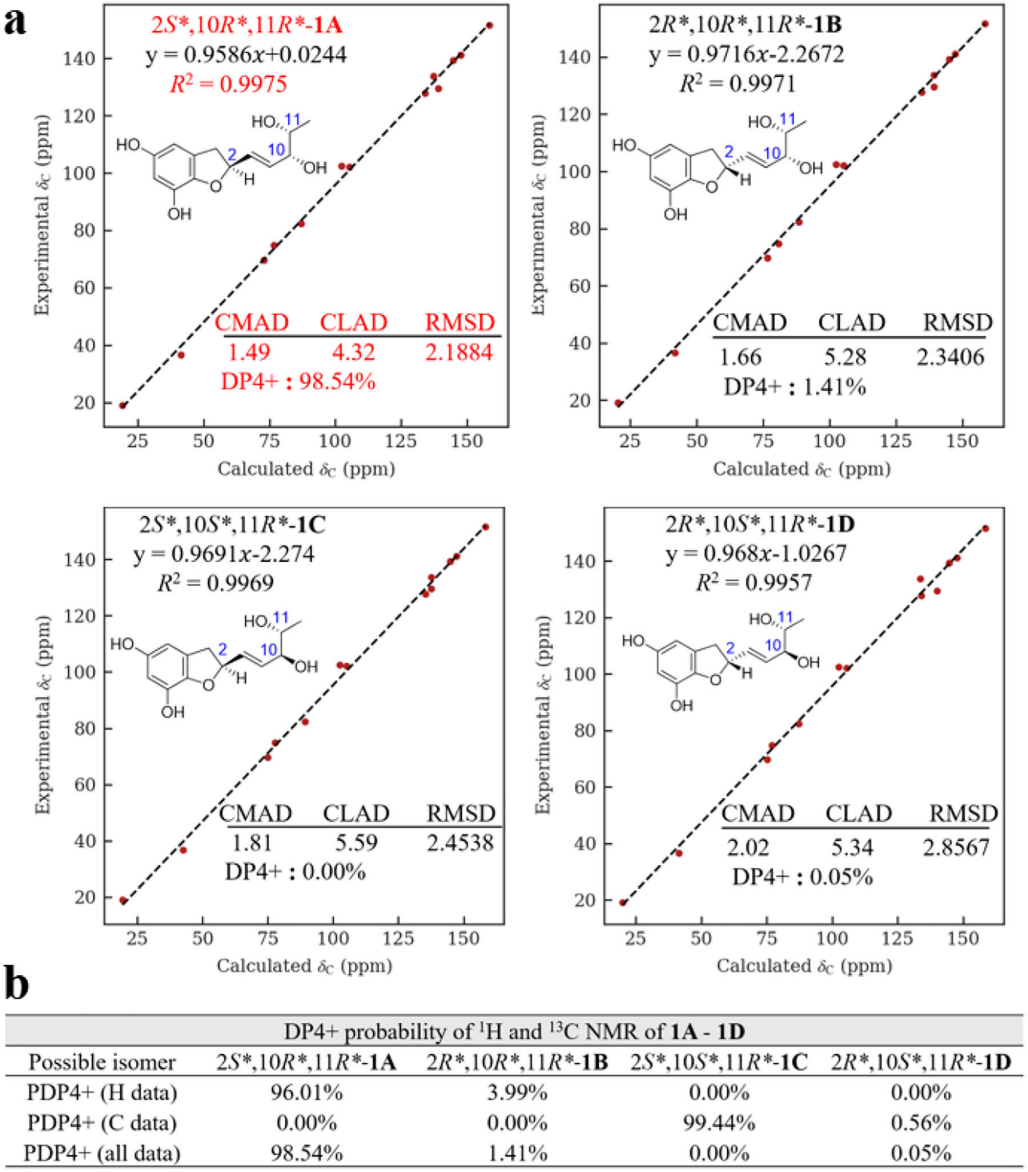
DP4+ probability results for compound **1** derived from NMR calculations: linear regression plots of experimental versus calculated ^1^ ^3^C chemical shifts for the four candidate isomers, along with the corresponding DP4+ probabilities.

The large coupling constants (*J*_H‐8,H‐9_ = 15.7 Hz) indicated *E* configuration of the Δ^8^ double bond (Table 1). To establish the stereochemistry of C‐2, C‐10, and C‐11 **1**, theoretical calculations of NMR chemical shifts for four possible isomers **1A** (2S*, 10R*, 11R*), **1B** (2R*, 10R*, 11R*), **1C** (2S*, 10S*, 11R*), and **1D** (2R*, 10S*, 11R*) were performed using the GIAO method at the mPW1PW91/6‐311G(2d,p) level in DMSO with the IEFPCM model (Figure 3 and Table S5). As a result, the calculated ^13^C NMR chemical shifts of **1A** were consistent with those of the experimental data, with good linear correlation coefficients (*R* ^2^ = 0.9975), corrected mean absolute deviations (CMAD, 1.49), corrected largest absolute deviations (CLAD, 4.32), and root‐mean‐square deviations (RMSD, 2.1184) (Figure 3a). Further DP4+ analyses based on both ^1^H and ^13^C NMR data indicated that **1A** (2S*, 10R*, 11R*) was the most reliable structure with 98.54% probability (Figure 3b and Table S2). The NOESY correlation between 10‑OH and 11‑OH provided auxiliary evidence that further supports this assignment (Figure 2).

**FIGURE 4:**
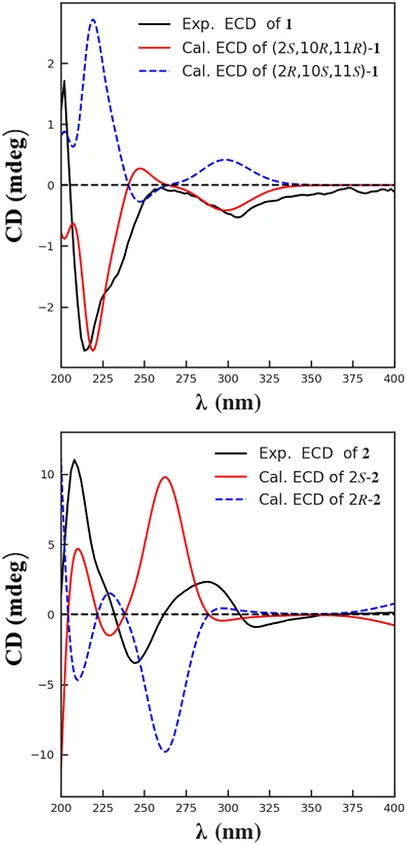
Experimental and calculated ECD spectra of compounds **1** and **2**.

To further determine the absolute configuration of **1**, a theoretical calculation of the ECD spectrum for (2*S*, 10*R*, 11*R*)‐**1** and its enantiomer (2*R*, 10*S*, 11*S*)‐**1** was performed (Figure S63 and Table S3). As shown in Figure 4, the experimental ECD spectrum matched well with the calculated one of (2*S*, 10*R*, 11*R*)‐**1**. Accordingly, compound **1** was identified as 10,11‐dihydro‐10,11‐dihydroasperfuran, and named flavasperfuran A.

Compound **2** was isolated as a brown oil. Its molecular formula was assigned as C_13_H_12_O_5_ by HRESIMS analysis at *m*/*z* 247.0618 [M − H]^−^ (calcd for C_13_H_11_O_5_, 247.0612), indicating eight degrees of unsaturation. The 1D NMR data (Table 1; Figures S9 and S10) of **2** were highly similar to those of **3** (Figures S17 and S18), except that a carbonyl unit (*δ*_C_ 167.9) was in C‐12 position in **2** instead of a methyl group (*δ*_H_ 1.72, *δ*_C_ 17.9) in **3**. This inference was substantiated by the correlations of H‐10 and H‐11 to the carbonyl carbon in the HMBC spectrum (Figures 2 and S13). Moreover, the experimental ECD spectrum of compound **2** closely aligned with the calculated spectrum for the (2*S*)‐isomer (Figures 4 and S64; Table S4). On the basis of the above evidences, compound **2** was identified as 12‐asperfuranoic acid, and named flavasperfuran B.

By comparing the NMR (Figures S17–S62), MS, and OR data with the references, 23 known compounds were identified as asperfuran (**3**) [20], speradine B (**4**) [21], cyclopiamide I (**5**) [22], speradine C (**6**) [21], speradine H (**7**) [23], speradine D (**8**) [21], pegriseofamine E (**9**) [24], cyclopiamide (**10**) [25], (3*S*,11a*S*)‐3‐[(1*H*‐indol‐3‐yl)methyl]‐7,9‐dihydroxy‐8‐methoxy‐2,3,11,11a‐tetrahydro‐6*H*‐pyrazino[1,2‐*b*]isoquinoline‐1,4‐dione (**11**) [26], hirudonucleodisulfide B (**12**) [27], *endo*‐d‐ditryptoleucine (**13**) [28], *exo*‐l‐ditryptoleucine (**14**) [28], deoxyleporin B (**15**) [29], cyclo‐(*N*‐methyl‐Trp‐Leu) (**16**) [30], uridine (**17**) [31], thymidine (**18**) [32], nortetillapyrone (**19**) [33], 1,3,9‐trimethyluric acid (**20**) [34], indazolinone (**21**) [35], 2‐(acetylamino)‐3‐hydroxy‐benzoic acid (**22**) [36], nicotinamide (**23**) [37], uracil (**24**) [37], and dihydrophaseic acid (**25**) [38].

**FIGURE 5:**
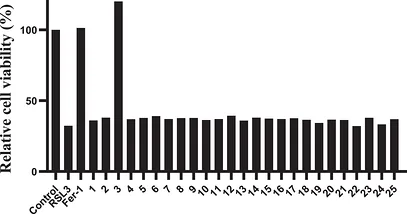
To evaluate ferroptosis, A375 cells were subjected to a 1 h pretreatment with each test compound (10 µM), followed by exposure to RSL3 (0.4 µM). The CellTiter‑Glo Luminescent assay was used to measure cell viability, and all values were normalized against the vehicle control (DMSO). The known inhibitor Fer‑1 (10 µM) was included as a positive reference.

**FIGURE 6:**
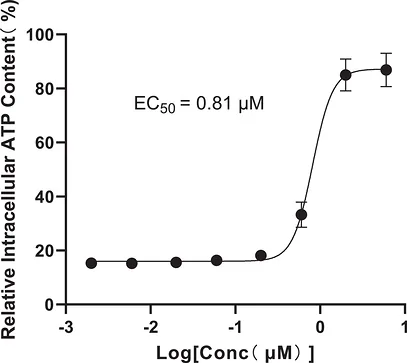
The RSL3‑triggered ferroptotic response in A375 cells was attenuated by compound **3** in a dose‑responsive fashion.

Ferroptosis is defined as a regulated, non‑apoptotic cell death modality driven by iron‑catalyzed lipid peroxidation and oxidative injury [39]. This death pathway has been implicated in diverse pathophysiological contexts, ranging from aging and neurodegenerative illnesses to stroke and organ ischemia‑reperfusion damage, implying that its pharmacological suppression might constitute a promising therapeutic approach for these conditions [40, 41]. The well‑known inducer RSL3 markedly diminishes A375 cell viability, whereas the ferroptosis antagonist ferrostatin‑1 (Fer‑1, 10 µM) completely reversed this RSL3‑induced cell death in the same cells (Figure 5). Asperfuran (**3**) exhibited the most potency with a half maximal effective concentration (EC_50_) value of 0.81 µM (Figure 6).

The secondary metabolite profile of the strain isolated in this study exhibited substantial similarity to those reported for other marine‑derived *A. flavus* strains, particularly with regard to cyclopiazonic acid (CPA) alkaloids (**4**–**10**) [21] and diketopiperazines (**11**–**16**) [26]. Nucleosides (**17** and **18**), nicotinamide (**23**), and uracil (**24**) are primary metabolites commonly recovered from both marine and terrestrial *A. flavus* [42, 43]. In contrast, compounds **19**–**22** and **25** were found exclusively in this marine isolate and have not been reported from terrestrial sources to date. Interestingly, unlike terrestrial toxigenic isolates characterized by aflatoxin production, the present strain did not yield detectable levels of these mycotoxins [44]. Moreover, asperfuran derivatives (**1**–**3**) were discovered from *A. flavus* for the first time in this work. These results further demonstrate the chemical diversity of marine‑derived *A. flavus* and highlight its potential as a source of bioactive natural products.

## 3 Conclusions

In summary, two new asperfuran derivatives (**1** and **2**) and 23 known compounds (**3**–**25**) were isolated from the marine‐derived fungus *A. flavus* MCCC 3A00246. Asperfuran (**3**) exhibited potent inhibition against RSL3‐induced ferroptosis with an EC_50_ value of 0.81 µM.

## 4 Experimental Section

### 4.1 General Experimental Procedures

The isolation workflow involved MPLC on a Buchi Sepacore system, semi‑preparative HPLC on an Agilent 1260 Infinity fitted with an ODS‑C18 column (10 mm × 250 mm, 5 µm), and CC over silica gel or Sephadex LH‑20. TLC detection was achieved under UV illumination or by spraying with 10% H_2_SO_4_. For compound identification, optical rotations were measured with an Anton Paar MCP 100 polarimeter; NMR spectra were recorded on a JEOL ECZ Luminous 600 MHz spectrometer in DMSO‑*d*_6_; and HRESIMS spectra were collected on a Sciex ZenoTOF 7600, with data analysis performed using PeakView. A375 cells (human malignant melanoma cells, RRID: CVCL_0132) were obtained from Procell Life Science & Technology Co., Ltd. (Wuhan, China; catalog no. CL‐0014).

### 4.2 Biological Material

*Aspergillus flavus* (GenBank accession no. FJ798687) was isolated from a water sample (75 m) of the Western Pacific Ocean. The reference strain was purchased from the Marine Culture Collection of China (MCCC) with the accession number MCCC 3A00246.

### 4.3 Fermentation, Extraction, and Isolation

The microbial strain was cultured on potato dextrose agar (PDA) plates at 25°C for 3 days. A single colony was inoculated into 250 mL seed medium (potato dextrose broth) in a 1 L Erlenmeyer flask and incubated on a rotary shaker (150 rpm) at 25°C for 3 days. For large‐scale fermentation, 100 × 1 L Erlenmeyer flasks were prepared, each containing 100 g oats, 1.5 g sea salt, and 120 mL distilled water. The flasks were autoclaved at 121°C for 20 min, then each was inoculated with 10 mL of the seed culture. Incubation was carried out under static conditions at 25°C for 25 days. The fermented oats substrate was extracted with EtOAc for three times to give a crude extract (48 g). The EtOAc extract was then subjected to MPLC (silica gel, MeOH−CH_2_Cl_2_, 0 → 10%, v/v) to provide four fractions (Fr.1−Fr.4) based on the analysis of the thin‐layer chromatography (TLC) spots.

Fraction Fr.1 (3.7 g) was separated into two subfractions (Fr.1‐1–Fr.1‐2) by MPLC over ODS with MeOH–H_2_O (30% → 70%). Subfraction Fr.1‐1 (83 mg) was first subjected to column chromatography on Sephadex LH‐20 (MeOH) and then to HPLC (MeOH‐H_2_O, 50%, *v* = 3 mL/min) to yield **7** (8.2 mg, *t*_R_ = 32 min) and **9** (6 mg, *t*_R_ = 30 min). Subfraction Fr.1‐2 (98 mg) was separated by CC on Sephadex LH‐20 (MeOH) and then crystallized from MeOH to provide **13** (4 mg).

Fraction Fr.2 (4.2 g) was separated into four subfractions (Fr.2‐1–Fr.2‐4) by MPLC over ODS with MeOH–H_2_O (30% → 90%). Subfraction Fr.2‐1 (309 mg) was purified by CC on Sephadex LH‐20 (MeOH) followed by HPLC (MeOH‐H_2_O, 55%, *v* = 3 mL/min) to give **4** (7 mg, *t*_R_ = 22 min), **5** (10 mg, *t*_R_ = 19 min), **6** (126 mg, *t*_R_ = 26 min), and **16** (4.2 mg, *t*_R_ = 36 min). Subfraction Fr.2‐2 (45 mg) was purified by Sephadex LH‐20 (MeOH) to give **3** (6.8 mg). Subfractions Fr.2‐3 (102 mg) and Fr.2‐4 (91 mg) were subjected to CC on Sephadex LH‐20 (MeOH) and then HPLC (MeOH‐H_2_O, 75% → 90%, *v* = 3 mL/min) to yield **14** (6.8 mg, *t*_R_ = 23 min) and **15** (17.3 mg, *t*_R_ = 20 min), respectively.

Fr.3 (4 g) was separated into four subfractions (Fr.3‐1–Fr.3‐4) by MPLC over ODS (MeOH–H_2_O, 10% → 80%). Subfraction Fr.3‐1 (80 mg) was subjected to CC on Sephadex LH‐20 (MeOH) to yield **20** (7.9 mg). Subfraction Fr.3‐2 (47 mg) was separated by CC on Sephadex LH‐20 (MeOH) and then by HPLC (MeOH‐H_2_O, 30% → 60%, *v* = 3 mL/min) to yield **1** (5 mg, *t*_R_ = 16 min). Subfraction Fr.3‐3 (42 mg) was purified by CC on Sephadex LH‐20 (MeOH) and then by CC on silica gel (CH_2_Cl_2_–MeOH, 100:1) afford **8** (2.2 mg). Subfraction Fr.3‐4 (95 mg) was separated by CC on Sephadex LH‐20 (MeOH) and HPLC (C18, MeOH–H_2_O, 60% → 80%, *v* = 3 mL/min) to yield **10** (4 mg, *t*_R_ = 17 min).

Fraction Fr.4 (6.5 g) was separated into seven subfractions (Fr.4‐1–Fr.4‐7) by MPLC over ODS (MeOH–H_2_O, 5% → 70%). Fr.4‐1 (314 mg) was subjected to CC on Sephadex LH‐20 (MeOH) to afford **22** (7 mg). Fr.4‐2 (63 mg) was subjected to CC on Sephadex LH‐20 (MeOH), followed by HPLC (MeOH‐H_2_O, 15% → 40%, *v* = 3 mL/min) to afford **18** (8.4 mg, *t*_R_ = 20 min) and **21** (1.2 mg, *t*_R_ = 16 min). Subfraction Fr.4‐3 (59 mg) was separated by CC on Sephadex LH‐20 (MeOH) and then by PTLC (EtOAc‐MeOH, 50:1) to yield **23** (22 mg) and **25** (3 mg). Fr.4‐4 (31 mg) was purified by CC on Sephadex LH‐20 (MeOH) followed by HPLC (MeOH–H_2_O, 5%, *v* = 3 mL/min) to afford **19** (2.5 mg, *t*_R_ = 15 min). Subfraction Fr.4‐5 (302.7 mg) was subjected to CC on Sephadex LH‐20 (MeOH) and then purified by HPLC (MeOH–H_2_O, 45%, *v* = 3 mL/min) to afford **17** (2 mg, *t*_R_ = 24 min) and **24** (2.4 mg, *t*_R_ = 20 min). Subfraction Fr.4‐6 (70 mg) was separated by CC on Sephadex LH‐20 (MeOH) and HPLC (MeOH‐H_2_O, 45% → 70%, *v* = 3 mL/min) to afford **2** (4 mg, *t*_R_ = 15 min) and **12** (6 mg, *t*_R_ = 20 min). Subfraction Fr.4‐7 (117 mg) was separated by CC on Sephadex LH‐20 (MeOH) and HPLC (MeOH‐H_2_O, 40% → 60%, *v* = 3 mL/min) to afford **11** (4 mg, *t*_R_ = 18 min).

Flavasperfuran A (**1**): Brown oil; ${\left[\alpha \right]}_{D}^{25}$ − 33 (*c* 0.10, MeOH); UV (MeOH) *λ*_max_ (log *ε*) 278 (1.79), 290 (1.80) nm; ECD (MeOH) *λ*_max_ (Δ*ε*) 214 (−0.42), 260 (−0.01), 306 (−0.08) nm; ^1^H (600 MHz) and ^13^C (150 MHz) NMR data, see Table 1; HRESIMS *m*/*z* 275.0884 [M + Na]^+^ (calcd for C_13_H_16_O_5_Na, 275.0890).

Flavasperfuran B (**2**): Brown oil; ${\left[\alpha \right]}_{D}^{25}$ − 38 (*c* 0.10, MeOH); UV (MeOH) *λ*_max_ (log *ε*) 222 (2.13), 252 (2.47) nm; ECD (MeOH) *λ*_max_ (Δ*ε*) 208 (+1.66), 244 (−0.52), 288 (+0.35), 316 (−0.13) nm; ^1^H (600 MHz) and ^13^C (150 MHz) NMR data, see Table 1; HRESIMS *m*/*z* 247.0618 [M − H]^−^ (calcd for C_13_H_11_O_5_, 247.0612).

### 4.4 ECD Calculations

The conformational searches were performed with Gaussian 09. Conformers lying within an energy window of 7 kcal/mol were retained, using an RMSD cutoff of 0.2 Å and a maximum count of 100. These initial structures were first optimized via the semiempirical PM6 method in Gaussian 09, and any redundant conformations after this optimization were identified and excluded. The remaining conformers then underwent optimization at the HF/6‐31G(d) level, followed by a further refinement at the B3LYP/6‐31G(d) level. Harmonic vibrational frequencies were computed to verify that the optimized conformers were energy minima. For each conformer, the ECD spectrum was calculated using TDDFT at the B3LYP/6‐311G(d,p) level, with MeOH as the solvent and the IEFPCM solvation model implemented in Gaussian 09. The calculated and experimental ECD curves were plotted with a Gaussian broadening of *σ* = 0.3 eV and a UV shift of 10 nm, respectively.

### 4.5 NMR Calculation

The geometries required for NMR calculations were taken from the ECD results described above. Using the GIAO (Gauge‐Including Atomic Orbitals) method at the MPW1PW91/6‑311+G(2d,p) level, with DMSO solvation treated by the IEFPCM model, the magnetic shielding tensors were computed. The resulting chemical shifts, after referencing to TMS, were averaged according to Boltzmann population weights. Subsequently, the final set of ^1^ ^3^C NMR data was extracted through a linear regression analysis of these averaged values.

### 4.6 DP4+ Probability Analysis

The DP4+ probability analysis was performed on the basis of the computed shielding tensors and the experimental chemical shifts, utilizing the excel spreadsheet supplied by the original developers [45]. Within that template, the calculations were run with the mPW1PW91 functional, the 6‑311+G(d,p) basis set, and the PCM solvation model.

### 4.7 Ferroptosis Inhibitory Activity

The human melanoma A375 line was routinely cultured in Dulbecco's modified Eagle's medium (DMEM) containing 10% fetal bovine serum, 4 mM l‑glutamine, 100 IU penicillin, and 100 mg/mL streptomycin, and kept at 37°C in a humidified atmosphere with 5% CO_2_ [46]. The bioassay was carried out according to the published protocol [47]. Briefly, cells were placed into 96‑well plates, subjected to a 1‑h pre‑exposure to the test agents, and then challenged with RSL3 to evoke ferroptosis. Viability was monitored via the CCK‑8 assay, with absorbance recorded on a MULTISKAN GO microplate reader (Thermo Scientific); alternatively, the CellTiter‑Glo Luminescent kit was used following the manufacturer's instructions.

## Author Contributions

**Chun‐Xiu Wang**: investigation. **Zheng‐Biao Zou**: validation, writing – original draft, data curation. **Li‐Sheng Li**: investigation. **Cheng‐Hai Gao**: formal analysis. **Xian‐Wen Yang**: supervision, project administration, methodology.

## Conflicts of Interest

The authors declare no conflicts of interest.

## Supporting information




**Supporting File 1**: cbdv71713‐sup‐0001‐SuppMat.pdf.

## Data Availability

The data that supports the findings of this study are available in the Supporting Information of this article.
