# Supplementary material for: Flavasperfurans A and B, Two New Asperfuran Derivatives From the Marine‐Derived Fungus Aspergillus flavus
Source: Chem Biodivers. 2026 Sep 15;23(9):e71713. doi: 10.1002/cbdv.71713 (PMC13576079; doi:10.1002/cbdv.71713)
Supplement: Supplementary file 1 [file CBDV-23-e71713-s001.pdf]

# Content

**Figure S1.** $^{1}H$ NMR (600 MHz) spectrum of compound **1** in DMSO-$d_6$.
**Figure S2.** $^{13}C$ NMR (151 MHz) spectrum of compound **1** in DMSO-$d_6$.
**Figure S3.** HSQC spectrum of compound **1** in DMSO-$d_6$.
**Figure S4.** $^{1}H$-$^{1}H$ COSY spectrum of compound **1** in DMSO-$d_6$.
**Figure S5.** HMBC spectrum of compound **1** in DMSO-$d_6$.
**Figure S6.** NOESY spectrum of compound **1** in DMSO-$d_6$.
**Figure S7.** The negative HR-ESI-MS spectrum of compound **1**.
**Figure S8.** Optical rotation spectrum of compound **1**.
**Figure S9.** $^{1}H$ NMR (600 MHz) spectrum of compound **2** in DMSO-$d_6$.
**Figure S10.** $^{13}C$ NMR (151 MHz) spectrum of compound **2** in DMSO-$d_6$.
**Figure S11.** HSQC spectrum of compound **2** in DMSO-$d_6$.
**Figure S12.** $^{1}H$-$^{1}H$ COSY spectrum of compound **2** in DMSO-$d_6$.
**Figure S13.** HMBC spectrum of compound **2** in DMSO-$d_6$.
**Figure S14.** NOESY spectrum of compound **2** in DMSO-$d_6$.
**Figure S15.** The positive HR-ESI-MS spectrum of compound **2**.
**Figure S16.** Optical rotation spectrum of compound **2**.
**Figure S17.** $^{1}H$ NMR (600 MHz) spectrum of compound **3** in DMSO-$d_6$.
**Figure S18.** $^{13}C$ NMR (151 MHz) spectrum of compound **3** in DMSO-$d_6$.
**Figure S19.** $^{1}H$ NMR (600 MHz) spectrum of compound **4** in DMSO-$d_6$.
**Figure S20.** $^{13}C$ NMR (151 MHz) spectrum of compound **4** in DMSO-$d_6$.
**Figure S21.** $^{1}H$ NMR (600 MHz) spectrum of compound **5** in DMSO-$d_6$.
**Figure S22.** $^{13}C$ NMR (151 MHz) spectrum of compound **5** in DMSO-$d_6$.
**Figure S23.** $^{1}H$ NMR (600 MHz) spectrum of compound **6** in DMSO-$d_6$.
**Figure S24.** $^{13}C$ NMR (151 MHz) spectrum of compound **6** in DMSO-$d_6$.
**Figure S25.** $^{1}H$ NMR (600 MHz) spectrum of compound **7** in DMSO-$d_6$.
**Figure S26.** $^{13}C$ NMR (151 MHz) spectrum of compound **7** in DMSO-$d_6$.
**Figure S27.** $^{1}H$ NMR (600 MHz) spectrum of compound **8** in DMSO-$d_6$.
**Figure S28.** $^{13}C$ NMR (151 MHz) spectrum of compound **8** in DMSO-$d_6$.
**Figure S29.** $^{1}H$ NMR (600 MHz) spectrum of compound **9** in DMSO-$d_6$.
**Figure S30.** $^{13}C$ NMR (151 MHz) spectrum of compound **9** in DMSO-$d_6$.
**Figure S31.** $^{1}H$ NMR (600 MHz) spectrum of compound **10** in DMSO-$d_6$.
**Figure S32.** $^{13}C$ NMR (151 MHz) spectrum of compound **10** in DMSO-$d_6$.
**Figure S33.** $^{1}H$ NMR (600 MHz) spectrum of compound **11** in DMSO-$d_6$.
**Figure S34.** $^{13}C$ NMR (151 MHz) spectrum of compound **11** in DMSO-$d_6$.
**Figure S35.** $^{1}H$ NMR (600 MHz) spectrum of compound **12** in DMSO-$d_6$.
**Figure S36.** $^{13}C$ NMR (151 MHz) spectrum of compound **12** in DMSO-$d_6$.
**Figure S37.** $^{1}H$ NMR (600 MHz) spectrum of compound **13** in $CDCl_3$.
**Figure S38.** $^{13}C$ NMR (151 MHz) spectrum of compound **13** in $CDCl_3$.
**Figure S39.** $^{1}H$ NMR (600 MHz) spectrum of compound **14** in DMSO-$d_6$.
**Figure S40.** $^{13}C$ NMR (151 MHz) spectrum of compound **14** in DMSO-$d_6$.
**Figure S41.** $^{1}H$ NMR (600 MHz) spectrum of compound **15** in DMSO-$d_6$.
**Figure S42.** $^{13}C$ NMR (151 MHz) spectrum of compound **15** in DMSO-$d_6$.

**Figure S43.** $^{1}$H NMR (600 MHz) spectrum of compound **16** in DMSO-$d_6$.
**Figure S44.** $^{13}$C NMR (151 MHz) spectrum of compound **16** in DMSO-$d_6$.
**Figure S45.** $^{1}$H NMR (600 MHz) spectrum of compound **17** in DMSO-$d_6$.
**Figure S46.** $^{13}$C NMR (151 MHz) spectrum of compound **17** in DMSO-$d_6$.
**Figure S47.** $^{1}$H NMR (600 MHz) spectrum of compound **18** in DMSO-$d_6$.
**Figure S48.** $^{13}$C NMR (151 MHz) spectrum of compound **18** in DMSO-$d_6$.
**Figure S49.** $^{1}$H NMR (600 MHz) spectrum of compound **19** in DMSO-$d_6$.
**Figure S50.** $^{13}$C NMR (151 MHz) spectrum of compound **19** in DMSO-$d_6$.
**Figure S51.** $^{1}$H NMR (600 MHz) spectrum of compound **20** in DMSO-$d_6$.
**Figure S52.** $^{13}$C NMR (151 MHz) spectrum of compound **20** in DMSO-$d_6$.
**Figure S53.** $^{1}$H NMR (600 MHz) spectrum of compound **21** in DMSO-$d_6$.
**Figure S54.** $^{13}$C NMR (151 MHz) spectrum of compound **21** in DMSO-$d_6$.
**Figure S55.** $^{1}$H NMR (600 MHz) spectrum of compound **22** in DMSO-$d_6$.
**Figure S56.** $^{13}$C NMR (151 MHz) spectrum of compound **22** in DMSO-$d_6$.
**Figure S57.** $^{1}$H NMR (600 MHz) spectrum of compound **23** in DMSO-$d_6$.
**Figure S58.** $^{13}$C NMR (151 MHz) spectrum of compound **23** in DMSO-$d_6$.
**Figure S59.** $^{1}$H NMR (600 MHz) spectrum of compound **24** in DMSO-$d_6$.
**Figure S60.** $^{13}$C NMR (151 MHz) spectrum of compound **24** in DMSO-$d_6$.
**Figure S61.** $^{1}$H NMR (600 MHz) spectrum of compound **25** in DMSO-$d_6$.
**Figure S62.** $^{13}$C NMR (151 MHz) spectrum of compound **25** in DMSO-$d_6$.
**Figure S63.** Gibbs free energy and equilibrium populations of low-energy conformers of **1** in ECD calculations.
**Figure S64.** Gibbs free energy and equilibrium populations of low-energy conformers of **2** in ECD calculations.
**Table S1.** Relative free energies and Boltzmann populations of low-energy conformers of compounds **1** and **2** within the 7 kcal/mol energy window
**Table S2.** Experimental and calculated chemical shifts of **1** for DP4+ probability statistical analysis.
**Table S3.** Cartesian coordinates for the low-energy reoptimized MMFF conformers of **1** at B3LYP/6-31G(d,p) level of theory in methanol.
**Table S4.** Cartesian coordinates for the low-energy reoptimized MMFF conformers of **2** at B3LYP/6-31G(d,p) level of theory in methanol.
**Table S5.** Gibbs free energies and boltzmann populations of all conformers of compound **1** included in the NMR calculationsa.

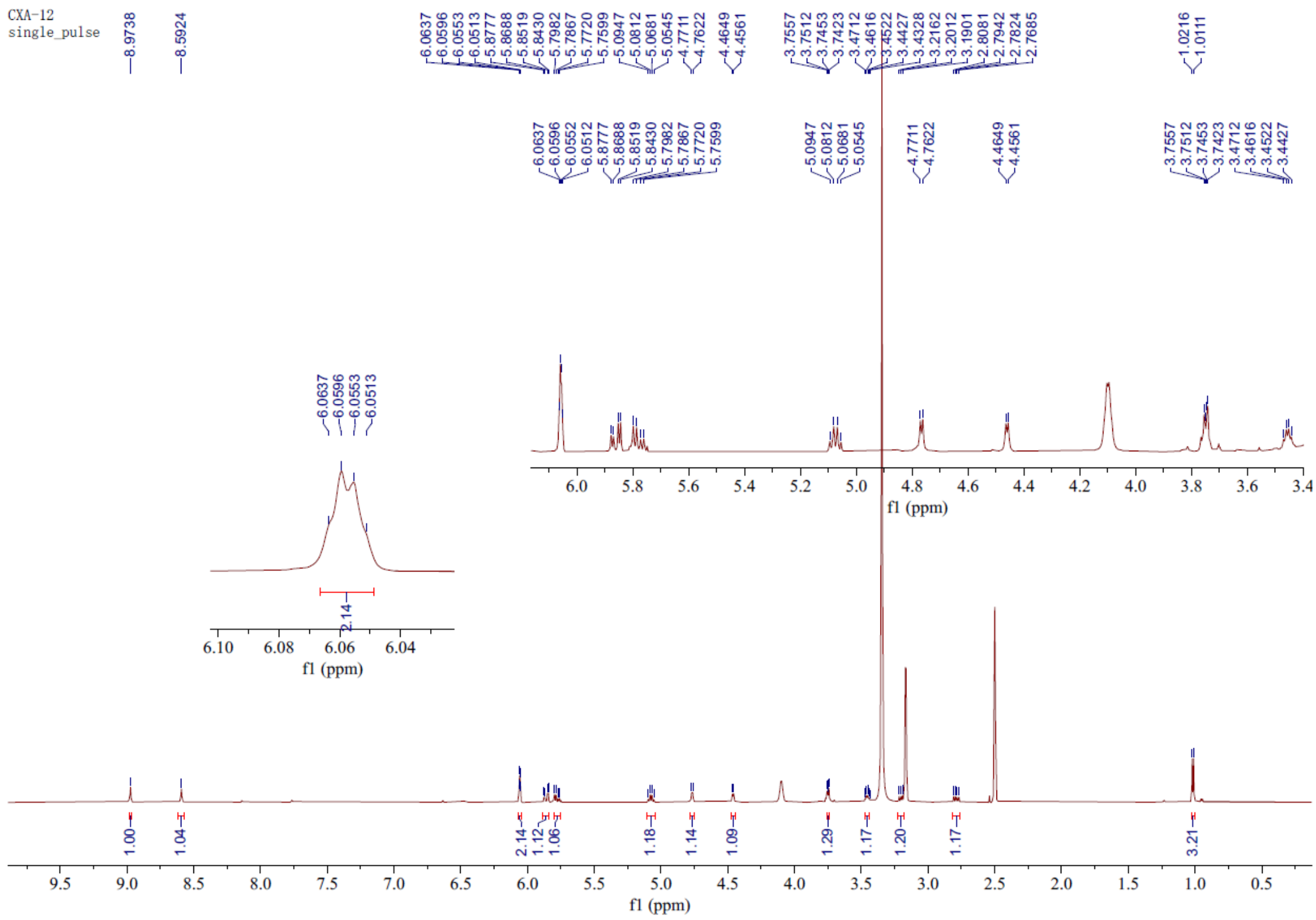


**Figure S1.** $^{1}H$ NMR (600 MHz) spectrum of compound **1** in DMSO-$d_6$.

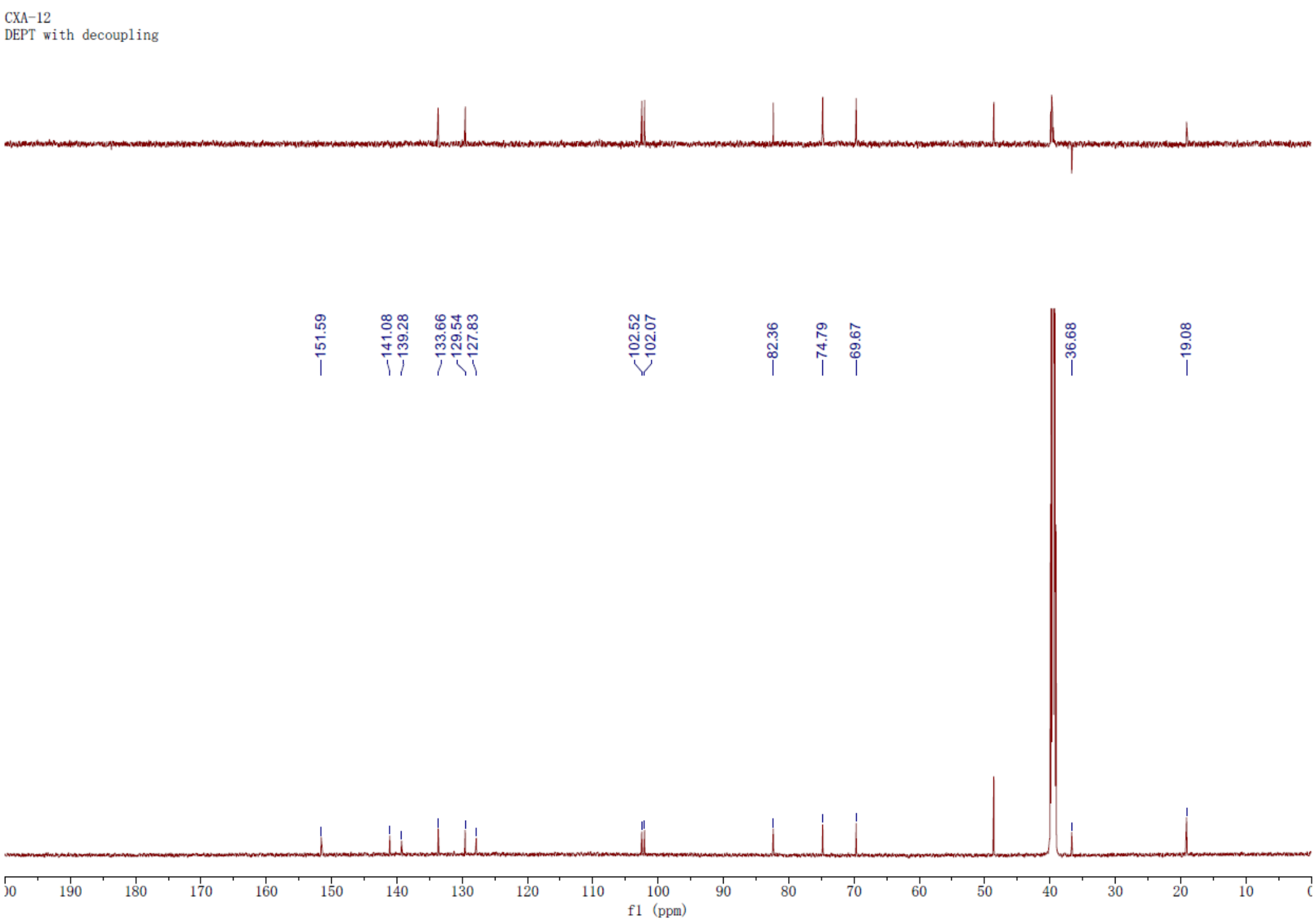


**Figure S2.** $^{13}C$ NMR (151 MHz) spectrum of compound **1** in DMSO-$d_6$.

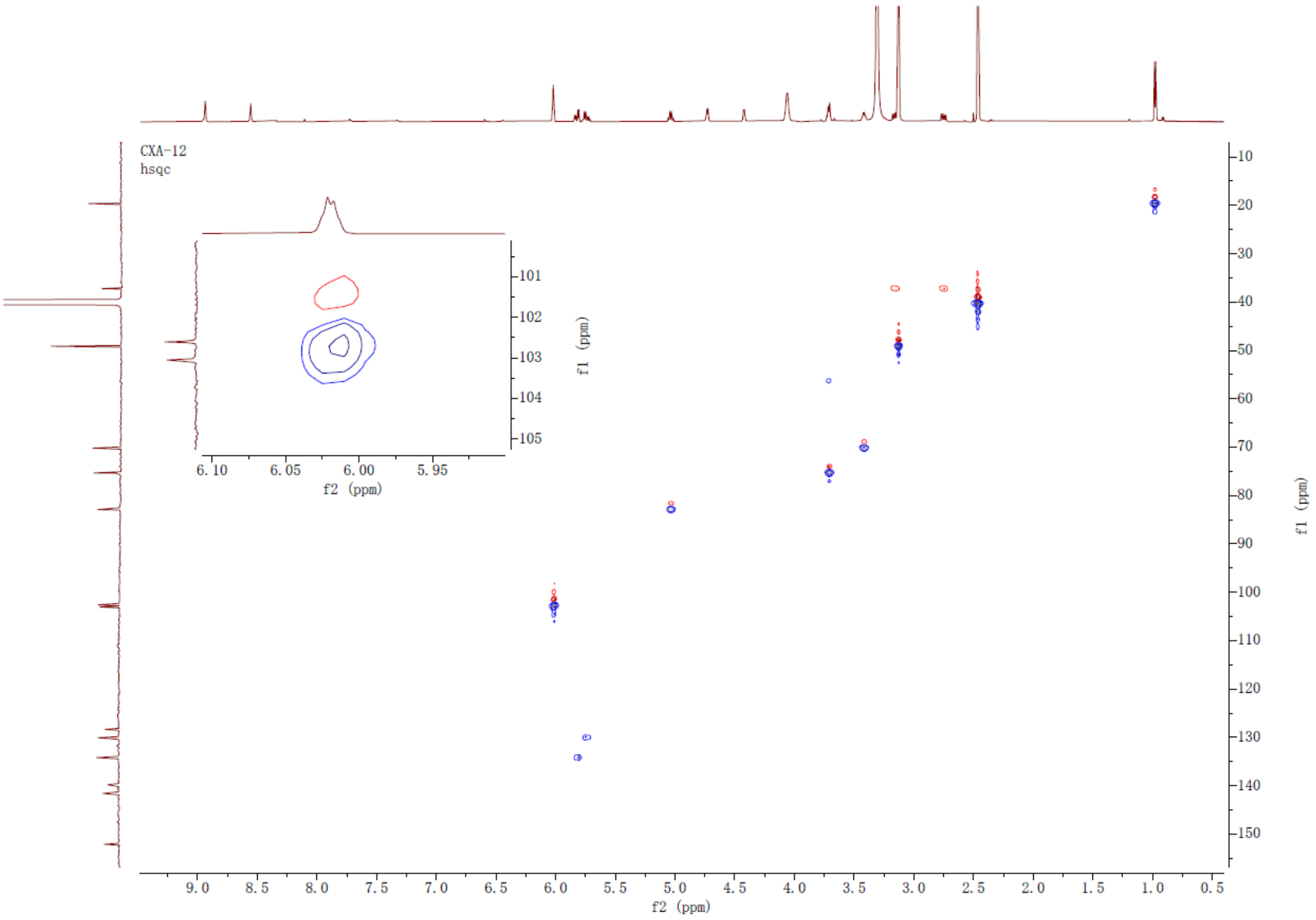


**Figure S3.** HSQC spectrum of compound **1** in DMSO-*d*6.

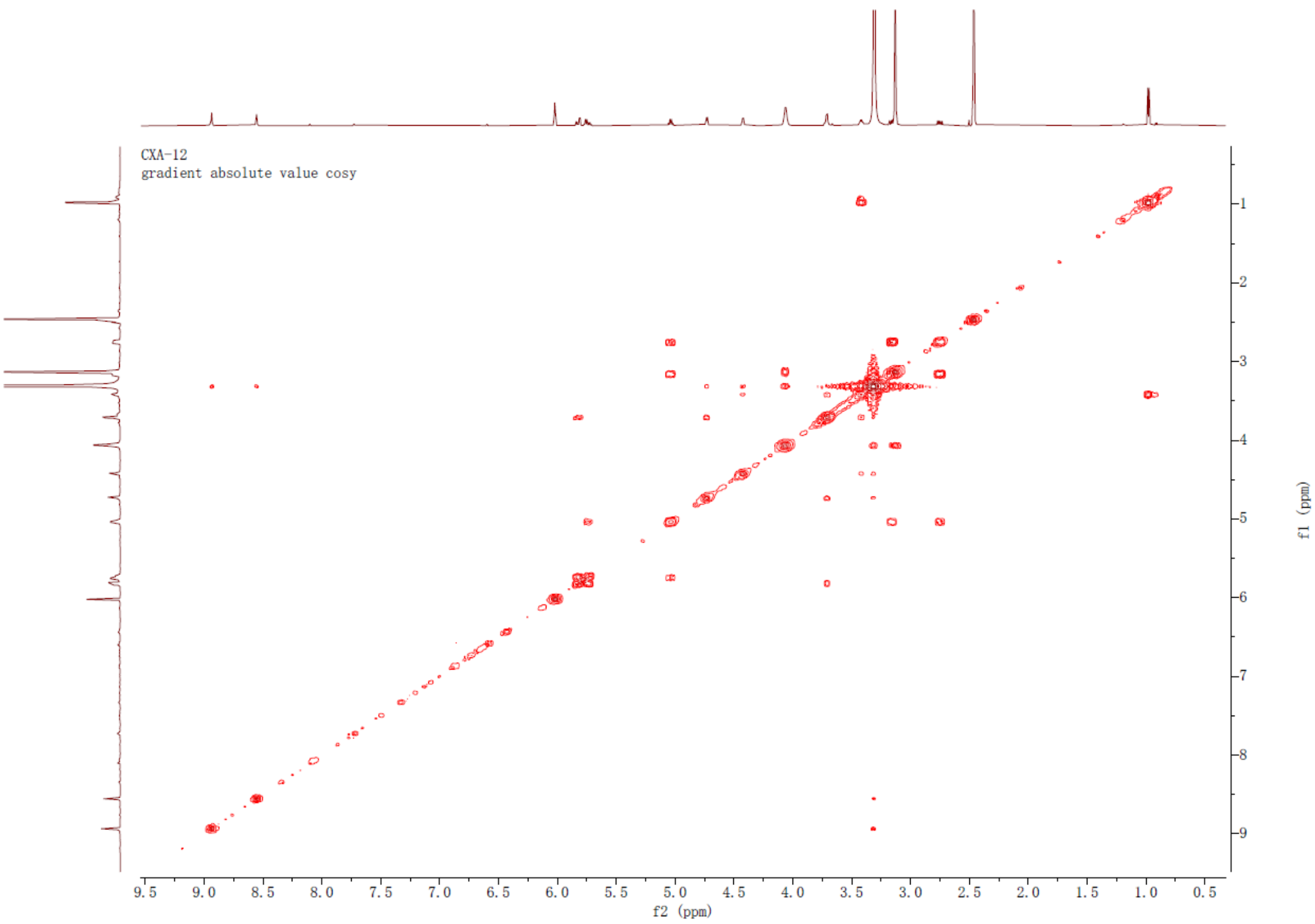


**Figure S4.** $^{1}$H-$^{1}$H COSY spectrum of compound **1** in DMSO-*d*6.

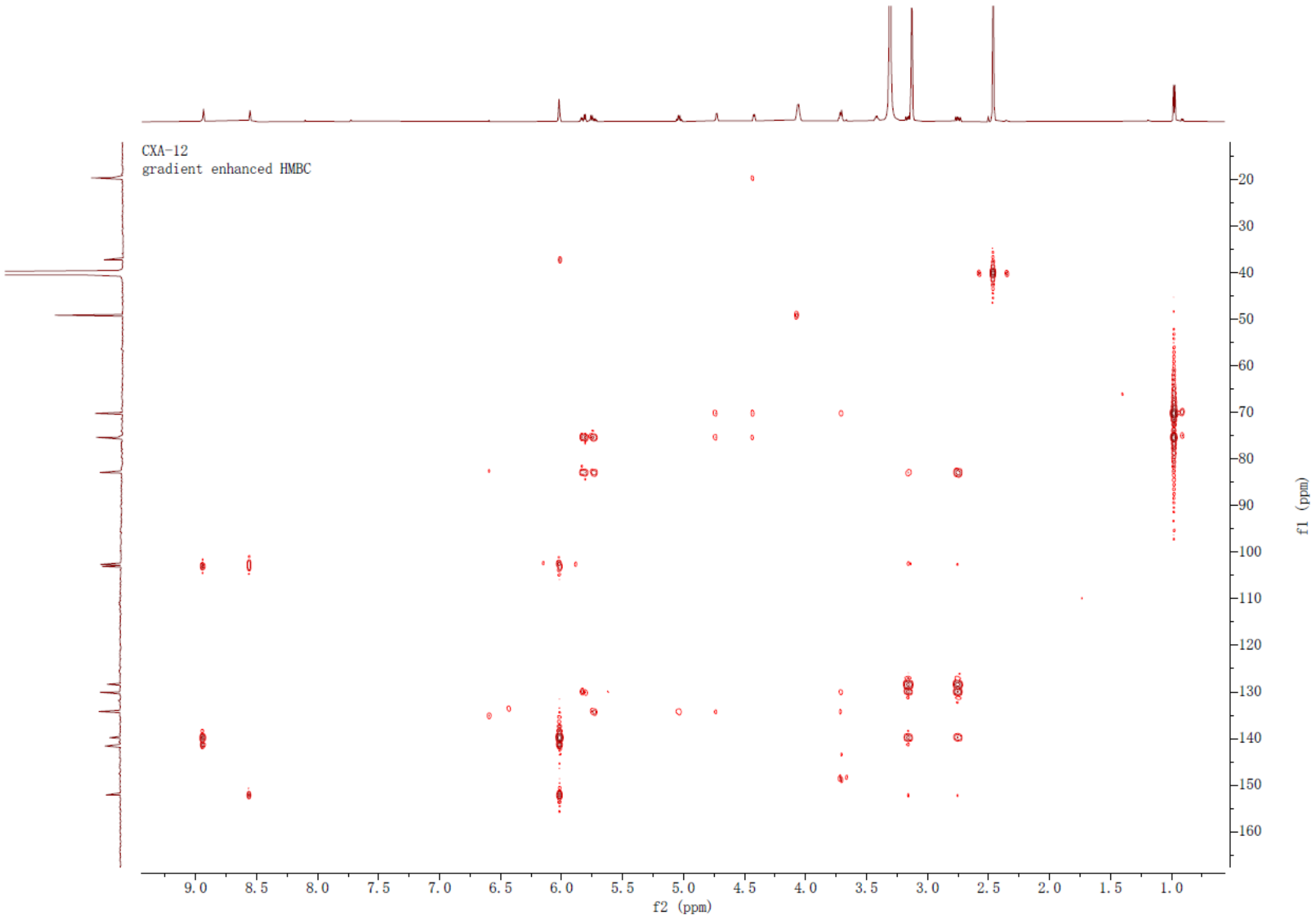


**Figure S5.** HMBC spectrum of compound **1** in DMSO-*d*6.

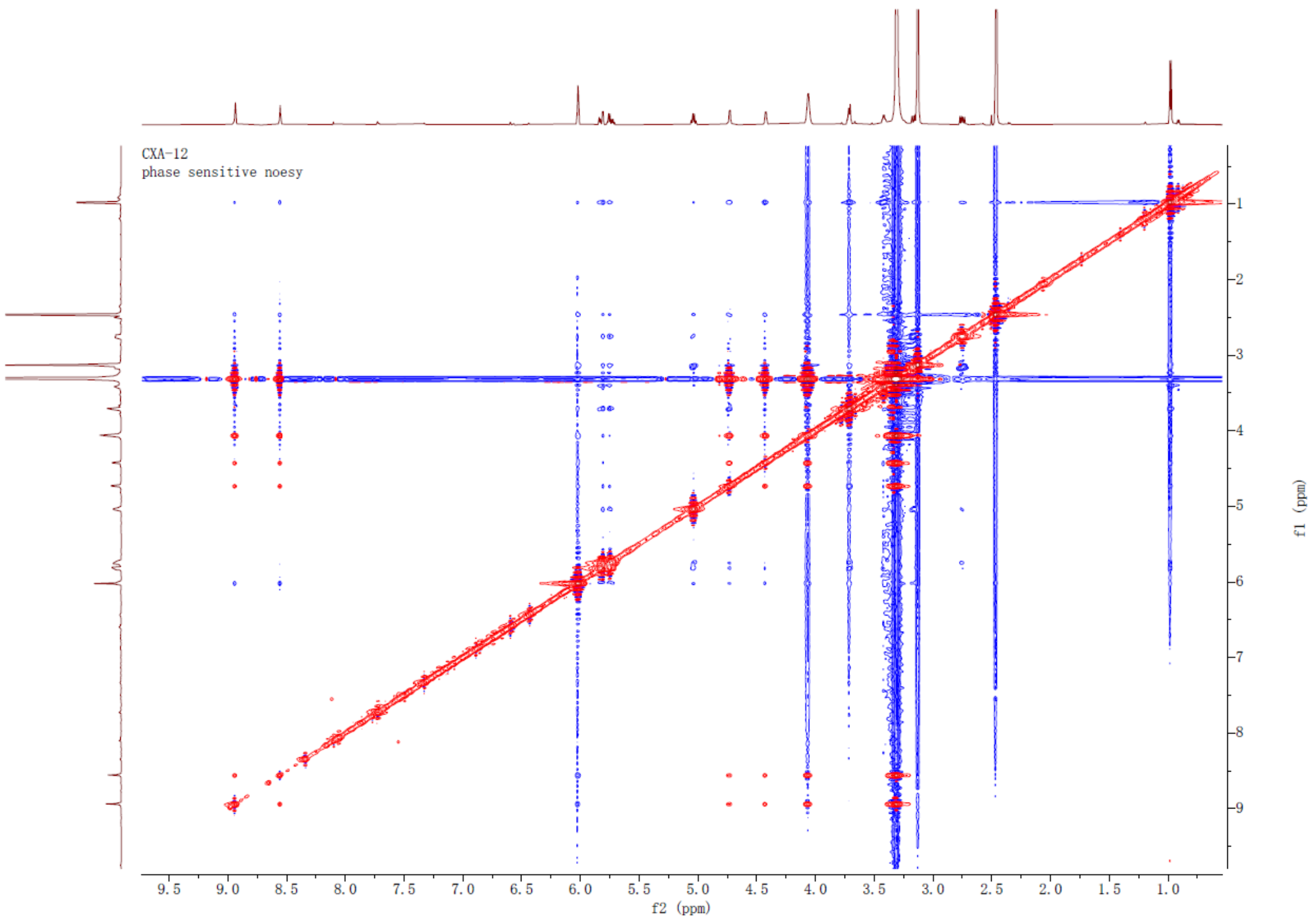


**Figure S6.** NOESY spectrum of compound **1** in DMSO-*d*6.

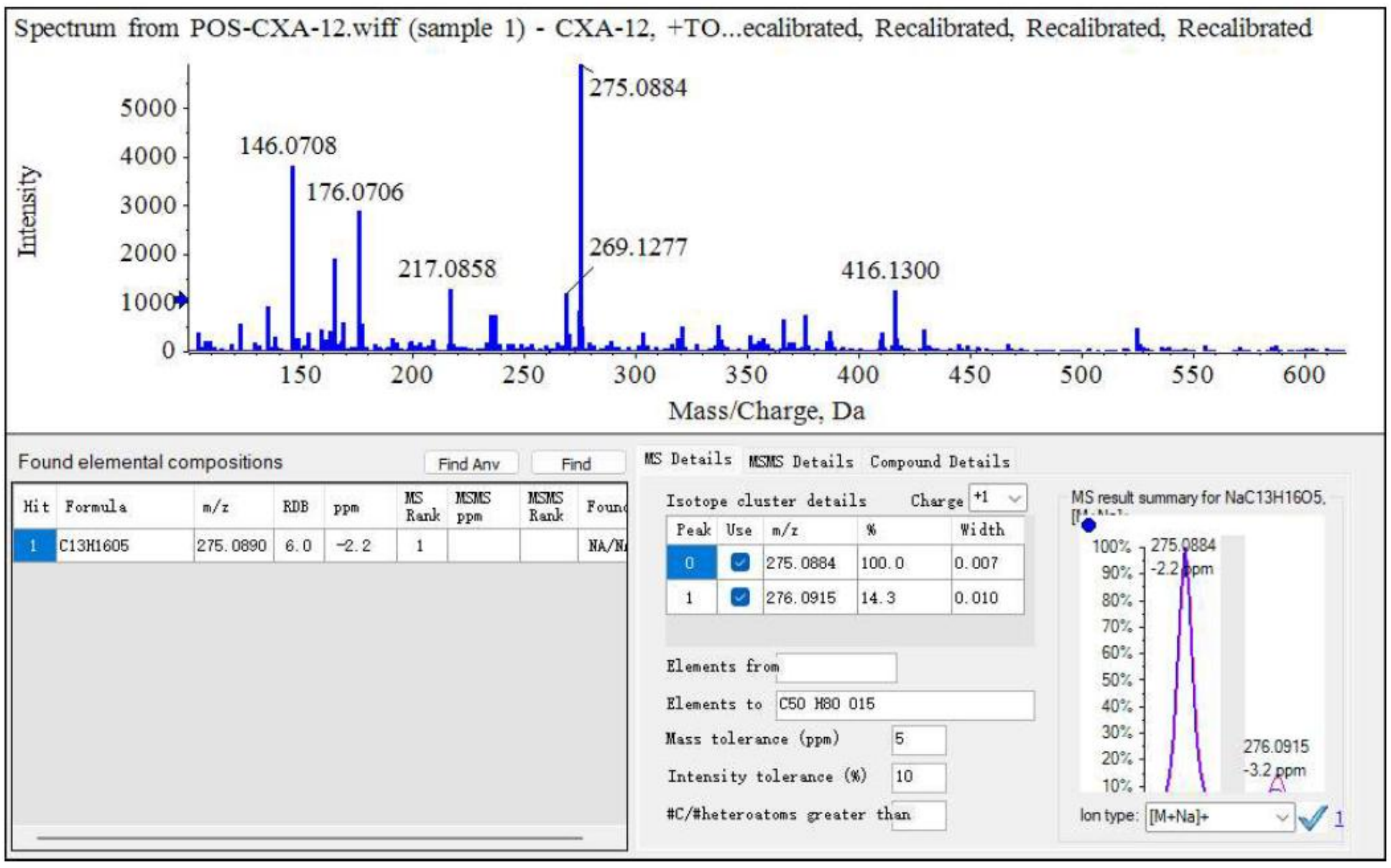


2026/6/1 9:15:30

**Figure S7.** The negative HR-ESI-MS spectrum of compound **1**.

Anton Paar GmbH
Anton Paar Strasse 10
8054 Graz
Austria

Anton Paar

## Anton Paar Polarimeter - Measurement(s)

MCP 100
Software Version: 1.50.4366.160
Serial Number: 99154608

### Unique Id 1568

- Sample Name: CXA-12
- Date: 04/23/2026 - 08:03 AM
- Username: Administrator
- Sample State: Ok

- Measurement Mode: Specific Rotation
- Measurement Result: -33.000 °
- Concentration: 0.100 g/100ml
- Optical Rotation: -0.033 °
- Set Temperature: 25.0 °C
- Temperature: 25.0 °C

**Figure S8.** Optical rotation spectrum of compound **1**.

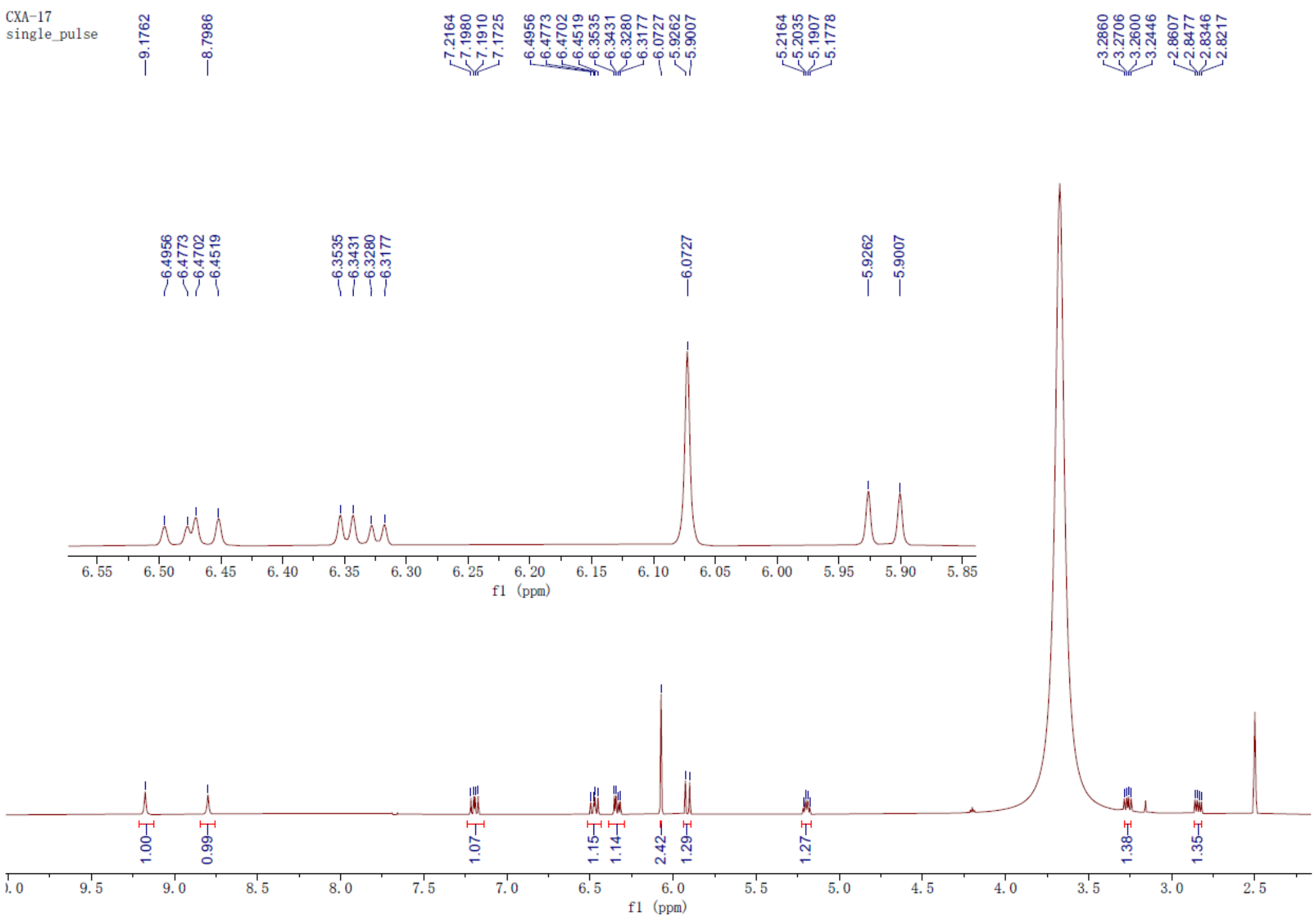


**Figure S9.** $^{1}$H NMR (600 MHz) spectrum of compound **2** in DMSO-*d*6.

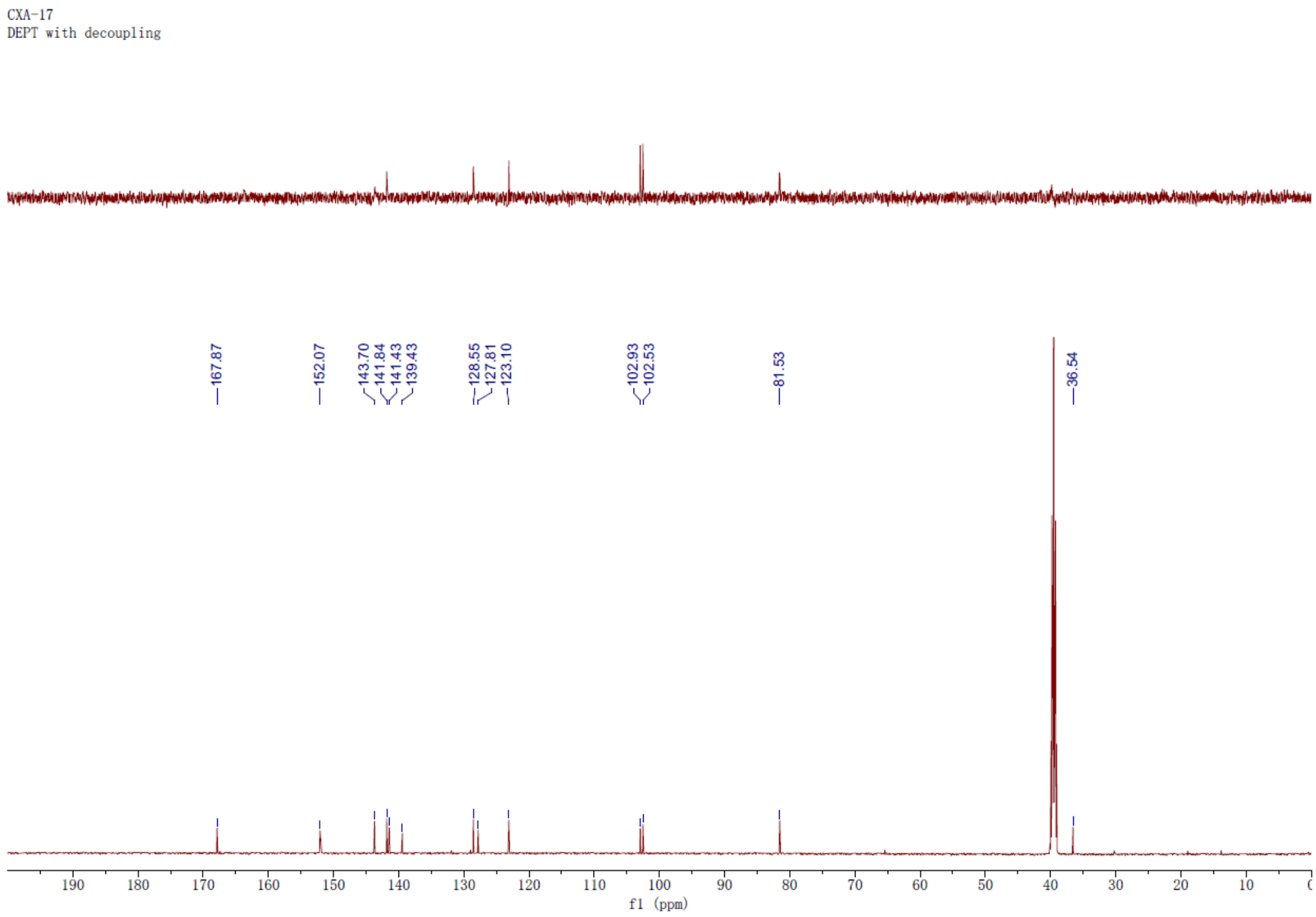


**Figure S10.** $^{13}$C NMR (151 MHz) spectrum of compound **2** in DMSO-*d*6.

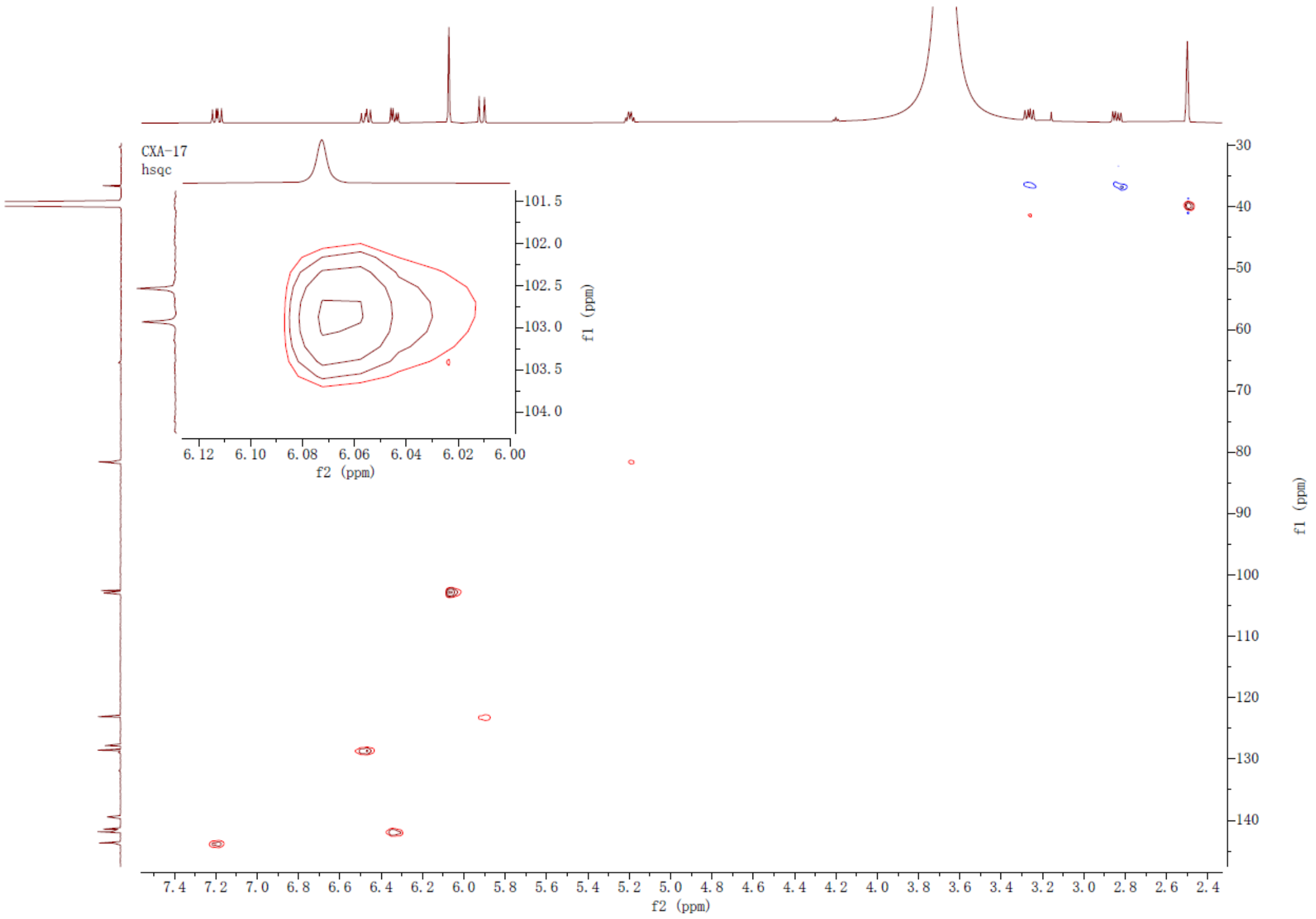


**Figure S11.** HSQC spectrum of compound **2** in DMSO-*d*6.

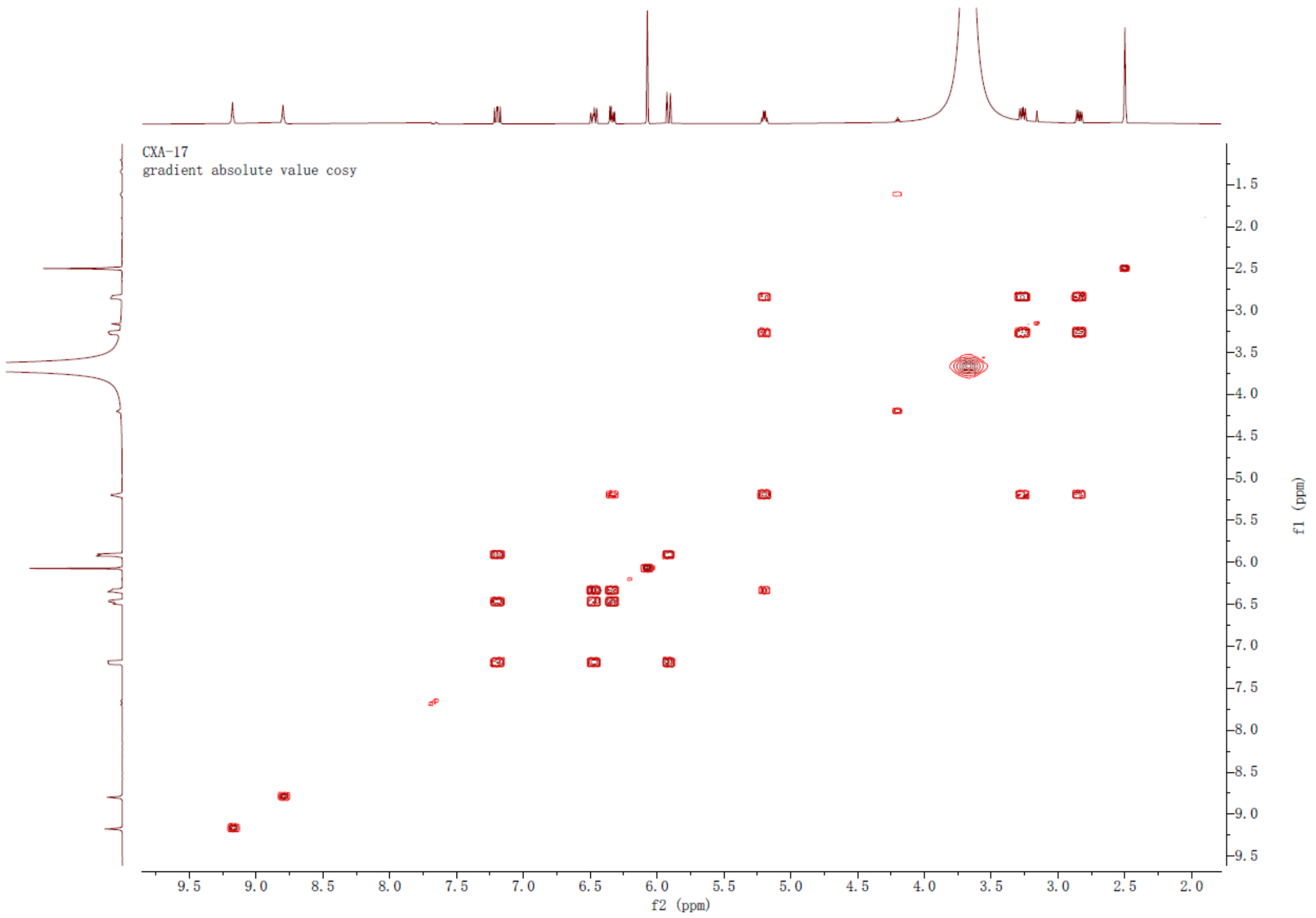


**Figure S12.** $^{1}$H-$^{1}$H COSY spectrum of compound **2** in DMSO-*d*6.

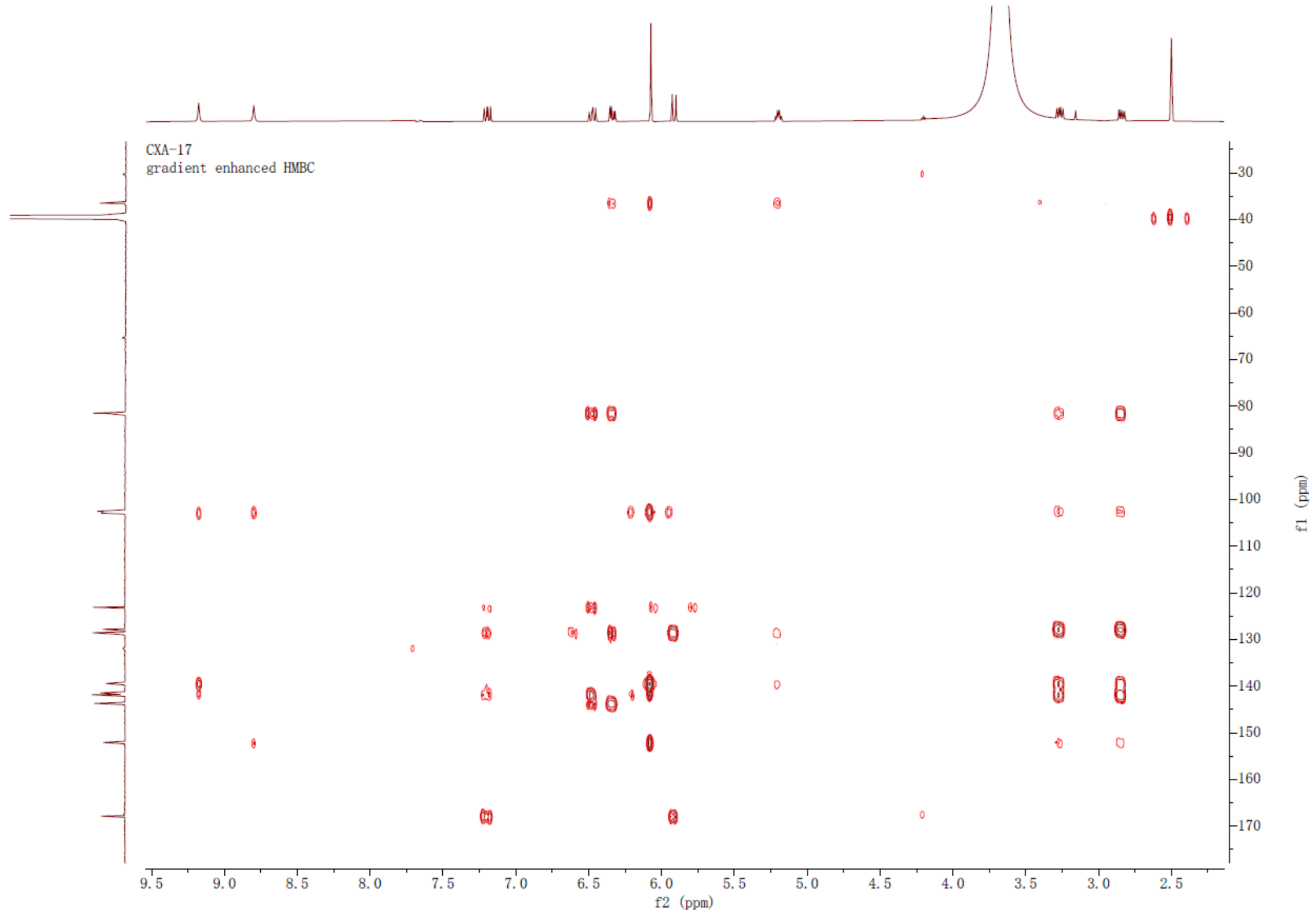


**Figure S13.** HMBC spectrum of compound **2** in DMSO-$d_6$.

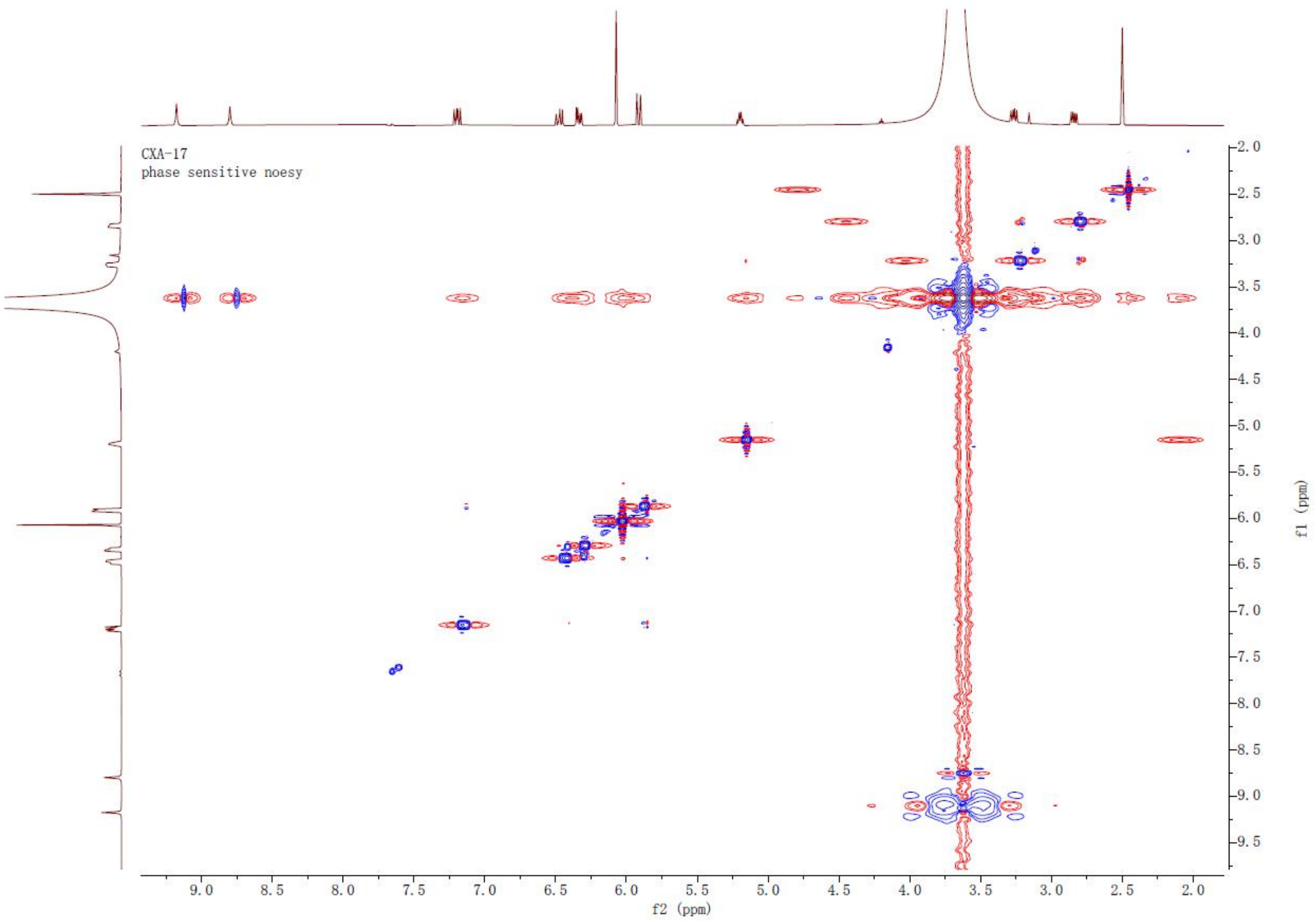


**Figure S14.** NOESY spectrum of compound **2** in DMSO-$d_6$.

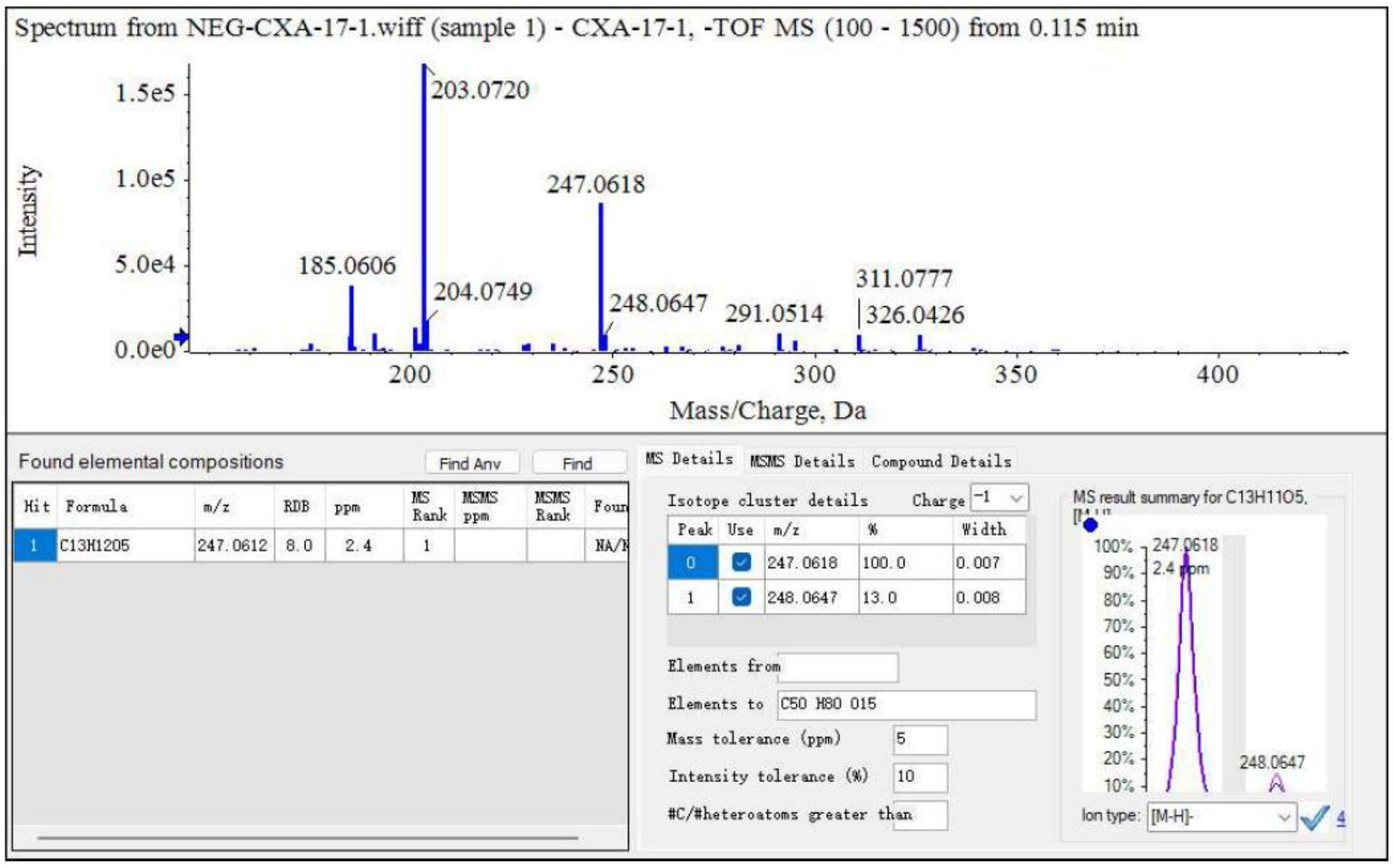


2026/6/1 9:26:24

**Figure S15.** The positive HR-ESI-MS spectrum of compound **2**.

Anton Paar GmbH
Anton Paar Strasse 10
8054 Graz
Austria

# Anton Paar Polarimeter - Measurement(s)

MCP 100
Software Version: 1.50.4366.160
Serial Number: 99154608

## Unique Id 1570

| | |
|---|---|
| ► Sample Name: | CXA-17 |
| ► Date: | 04/23/2026 - 08:09 AM |
| ► Username: | Administrator |
| ► Sample State: | Ok |

| | |
|---|---|
| ► Measurement Mode: | Specific Rotation |
| ► Measurement Result: | -38.000 ° |
| ► Concentration: | 0.100 g/100ml |
| ► Optical Rotation: | -0.038 ° |
| ► Set Temperature: | 25.0 °C |
| ► Temperature: | 25.0 °C |

**Figure S16.** Optical rotation spectrum of compound **2**.

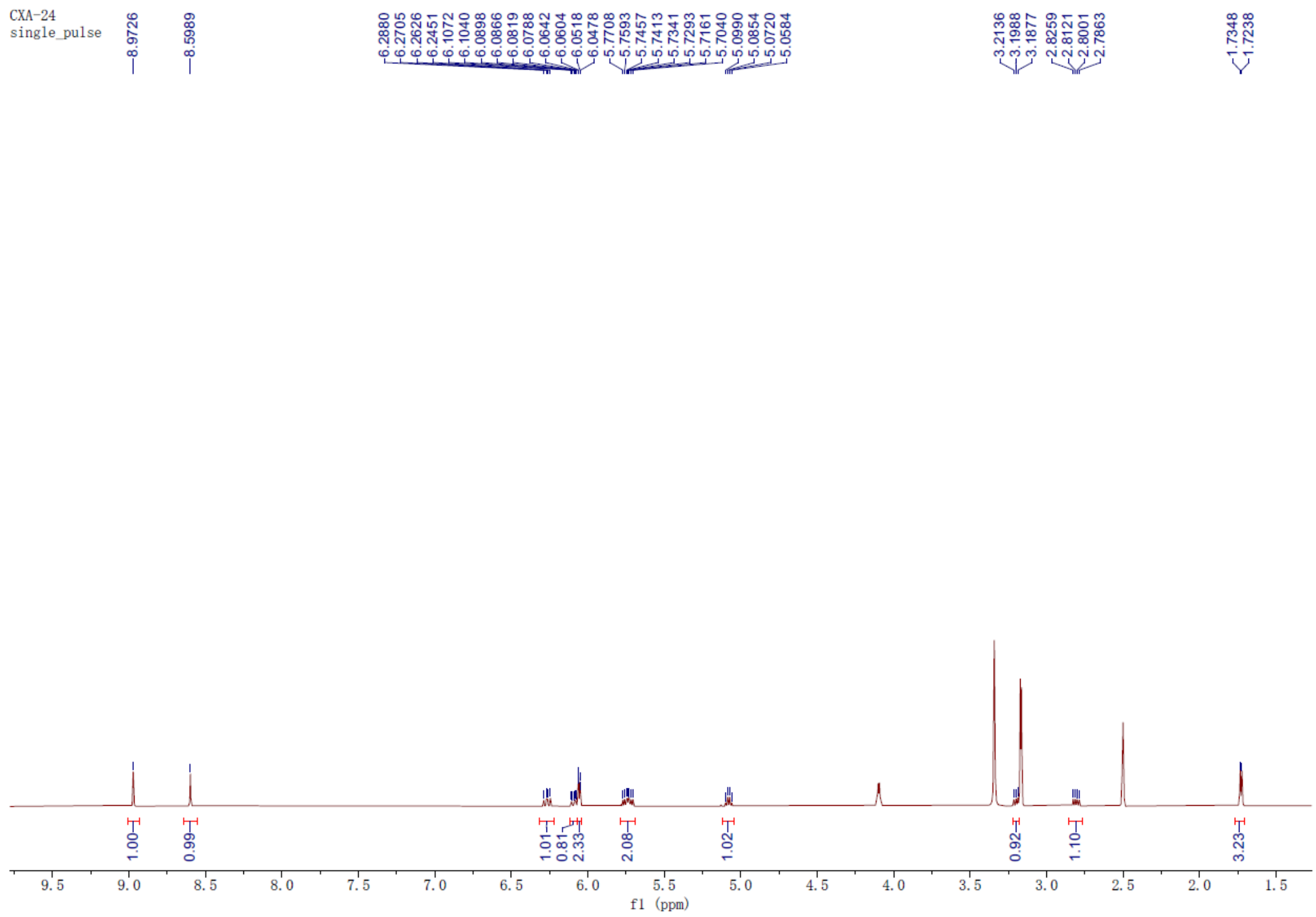

**Figure S17.** $^{1}H$ NMR (600 MHz) spectrum of compound **3** in DMSO-$d_6$.

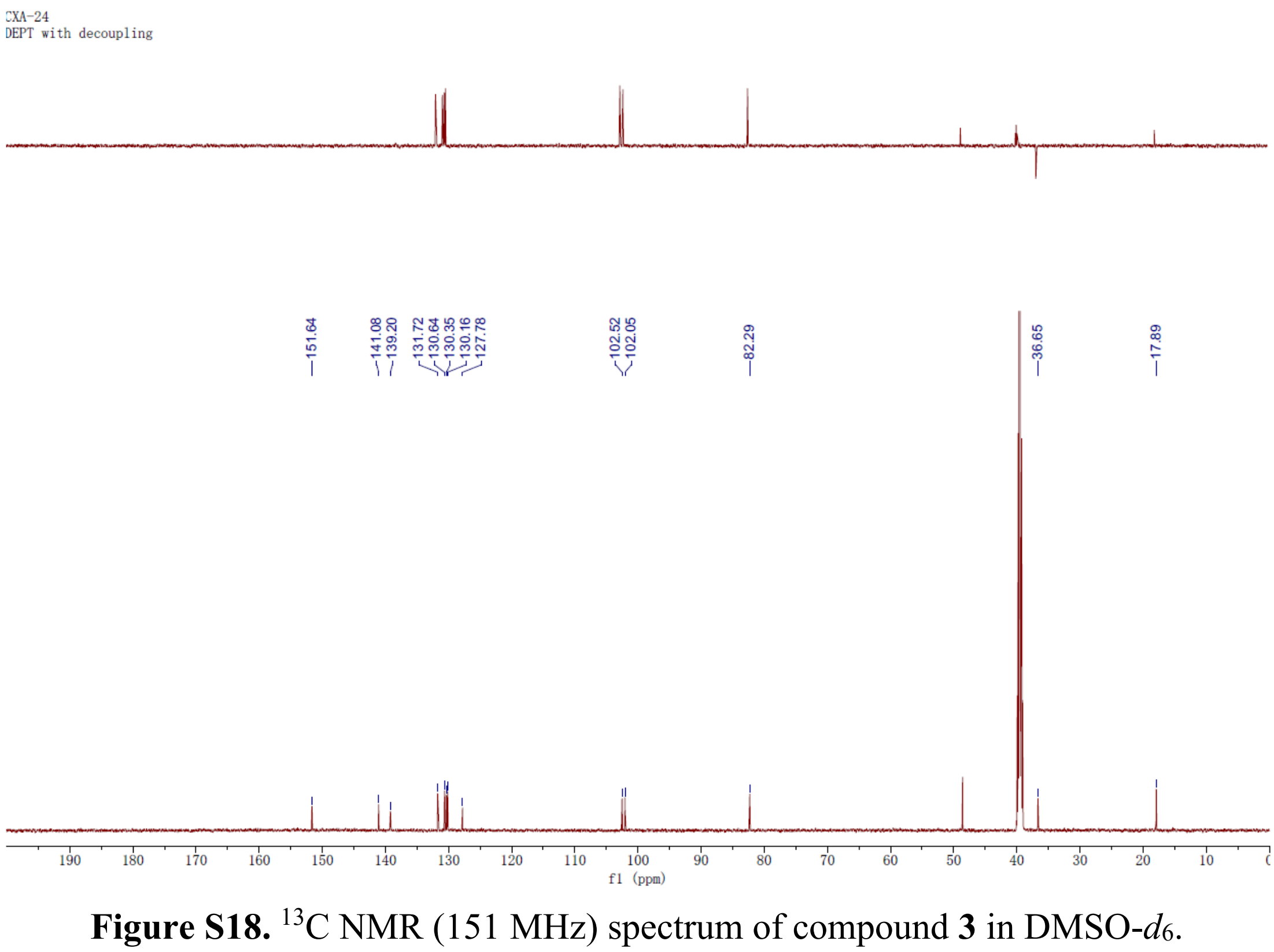

**Figure S18.** $^{13}C$ NMR (151 MHz) spectrum of compound **3** in DMSO-$d_6$.

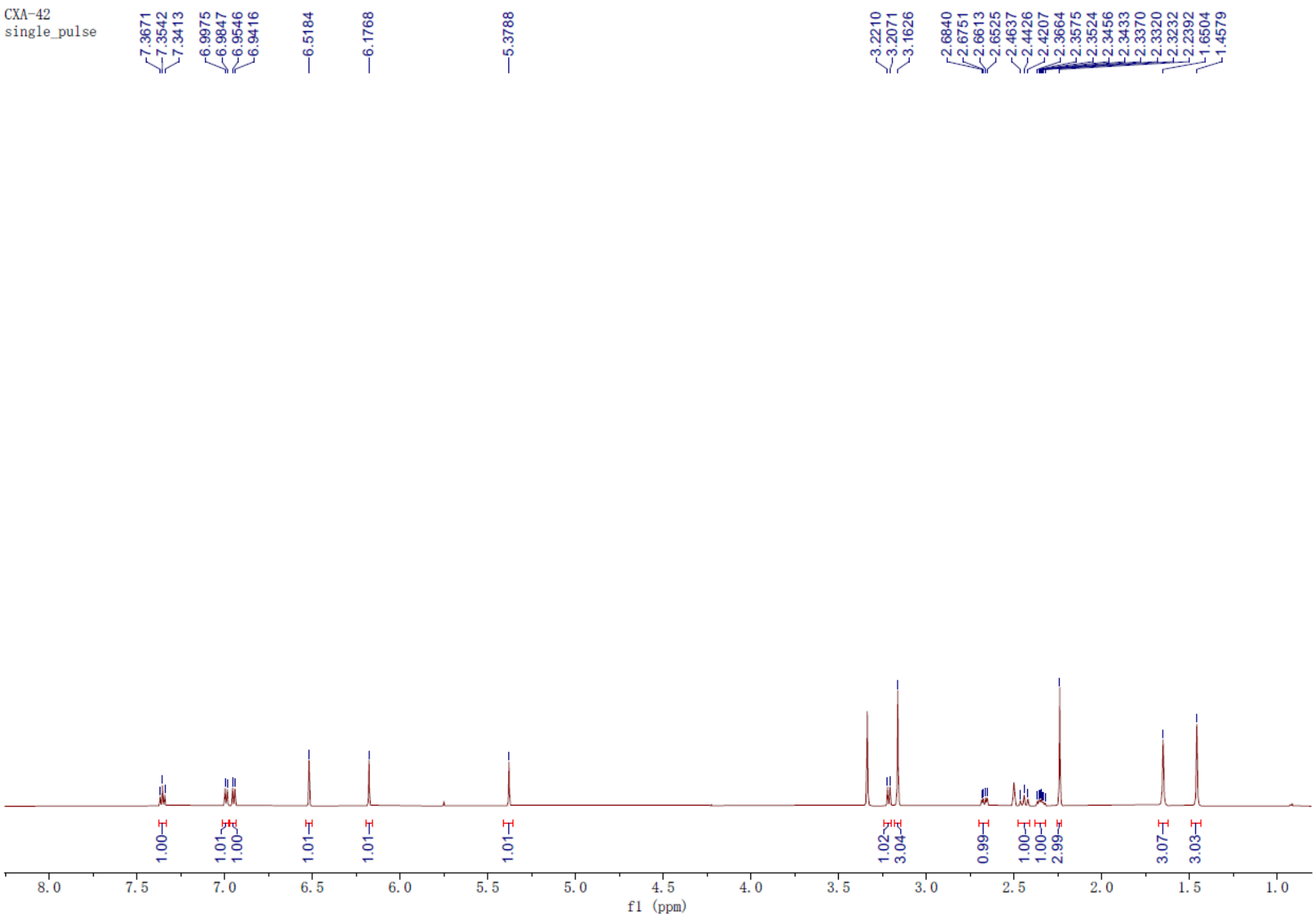


**Figure S19.** $^{1}$H NMR (600 MHz) spectrum of compound **4** in DMSO-$d_6$.

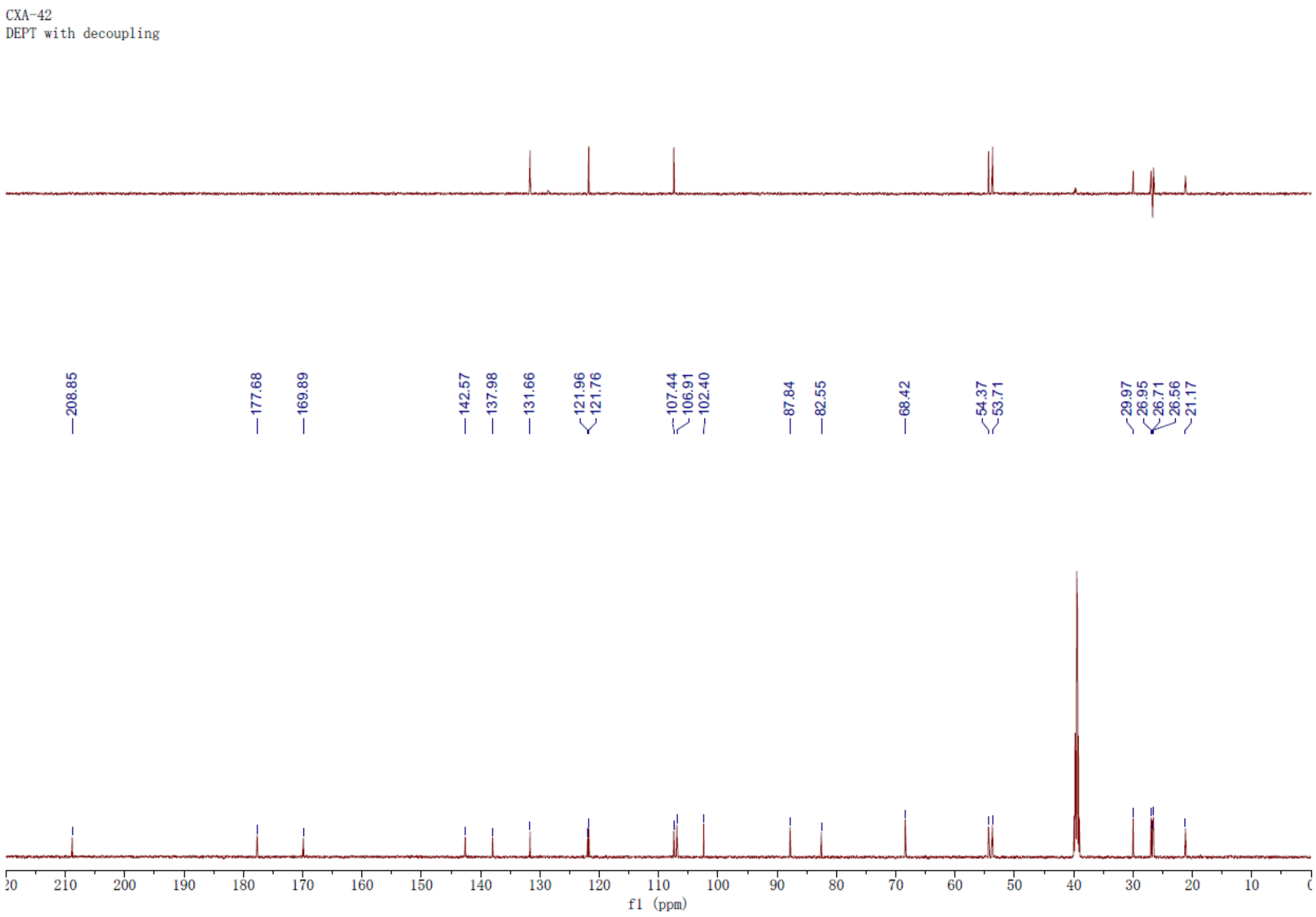


**Figure S20.** $^{13}$C NMR (151 MHz) spectrum of compound **4** in DMSO-$d_6$.

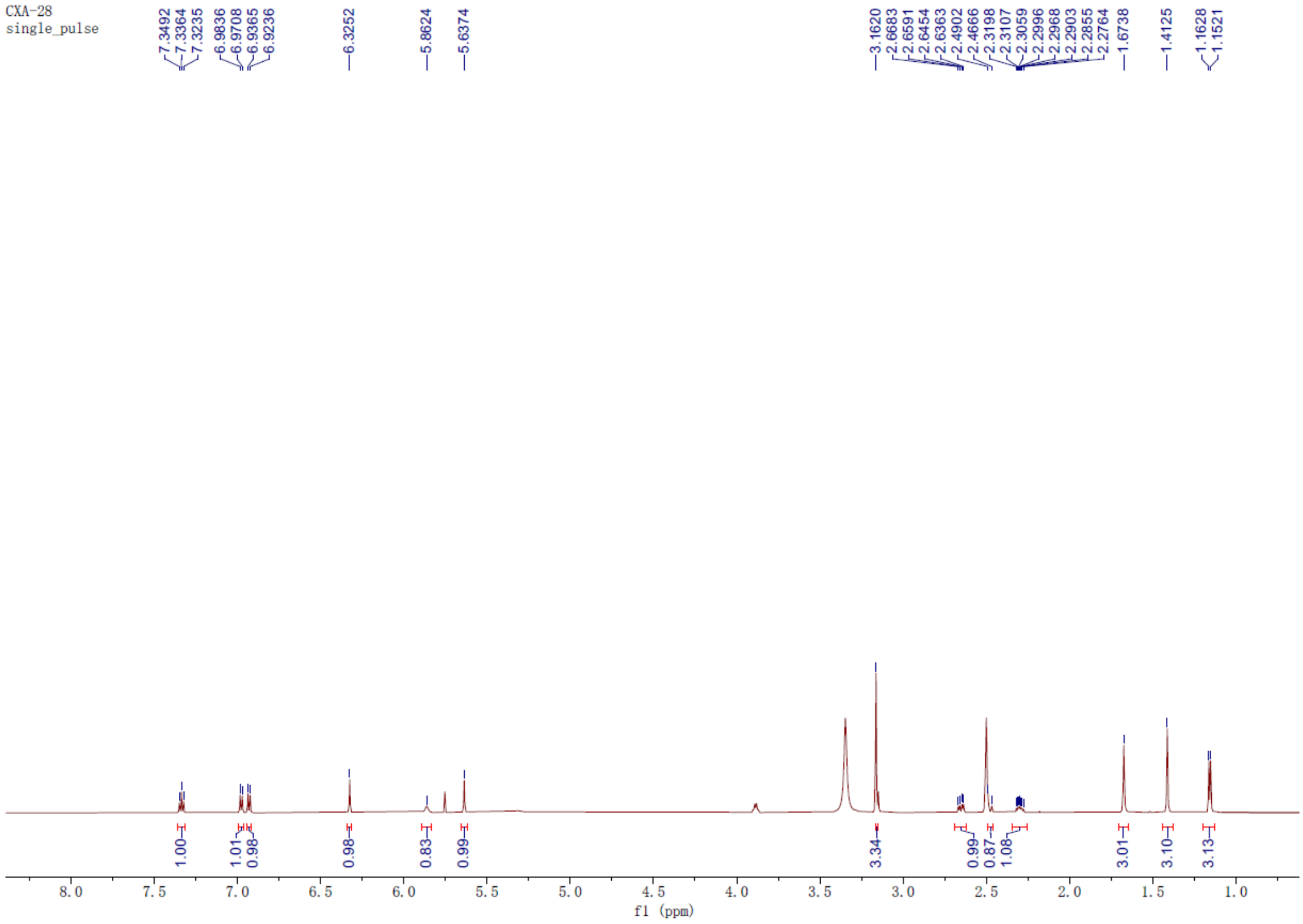


**Figure S21.** $^{1}H$ NMR (600 MHz) spectrum of compound **5** in DMSO-$d_6$.

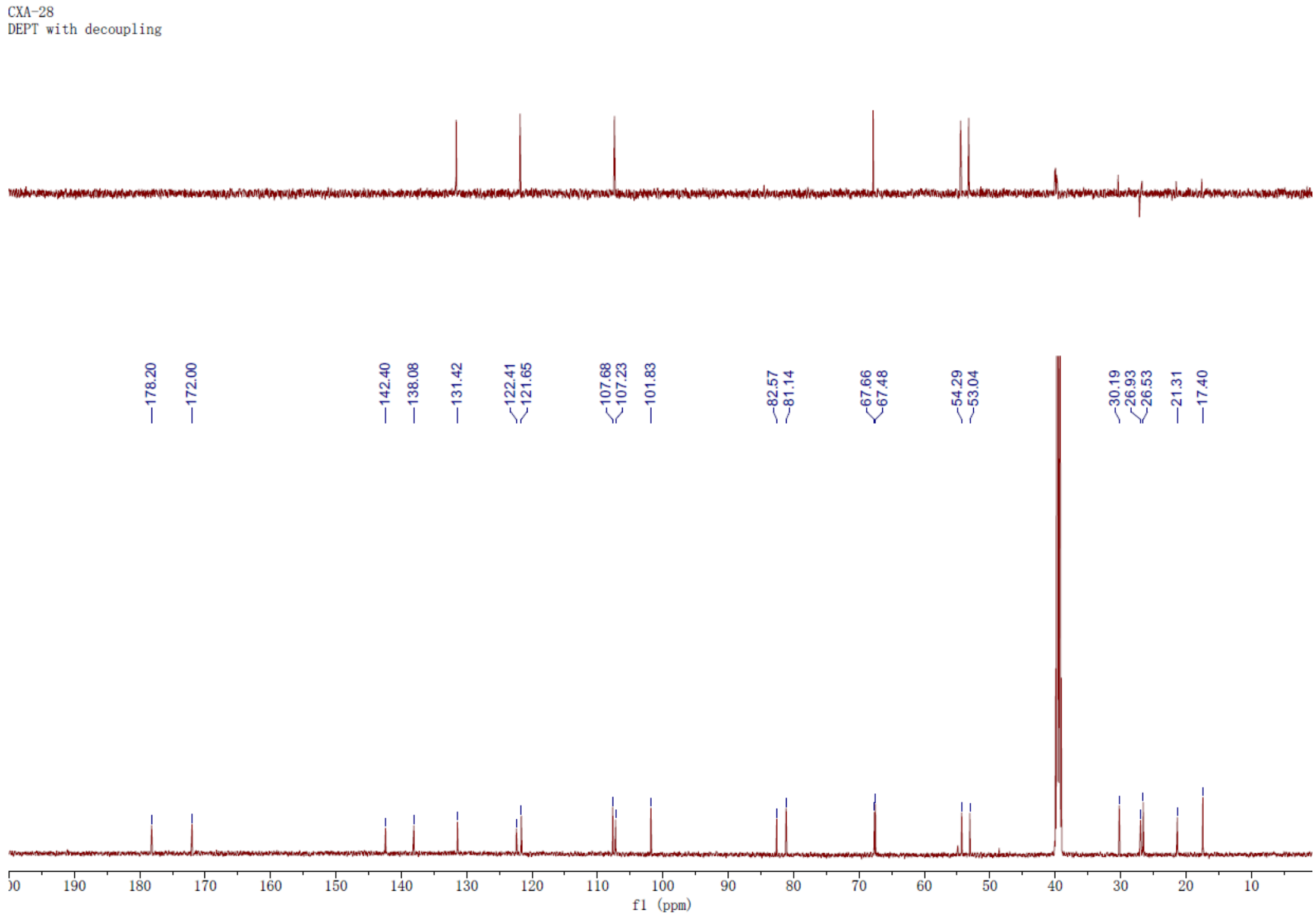


**Figure S22.** $^{13}C$ NMR (151 MHz) spectrum of compound **5** in DMSO-$d_6$.

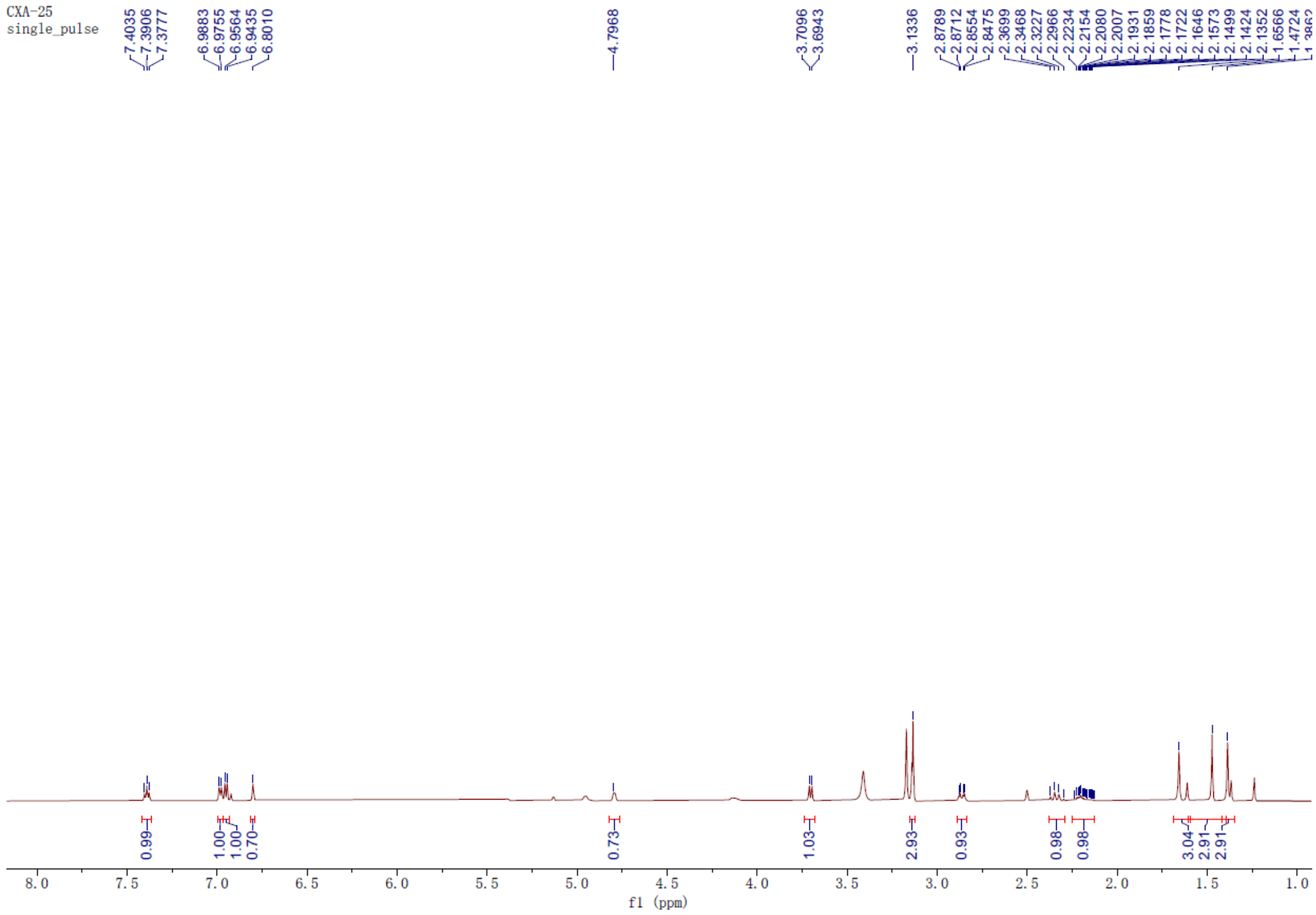

**Figure S23.** $^{1}H$ NMR (600 MHz) spectrum of compound **6** in DMSO-$d_6$.

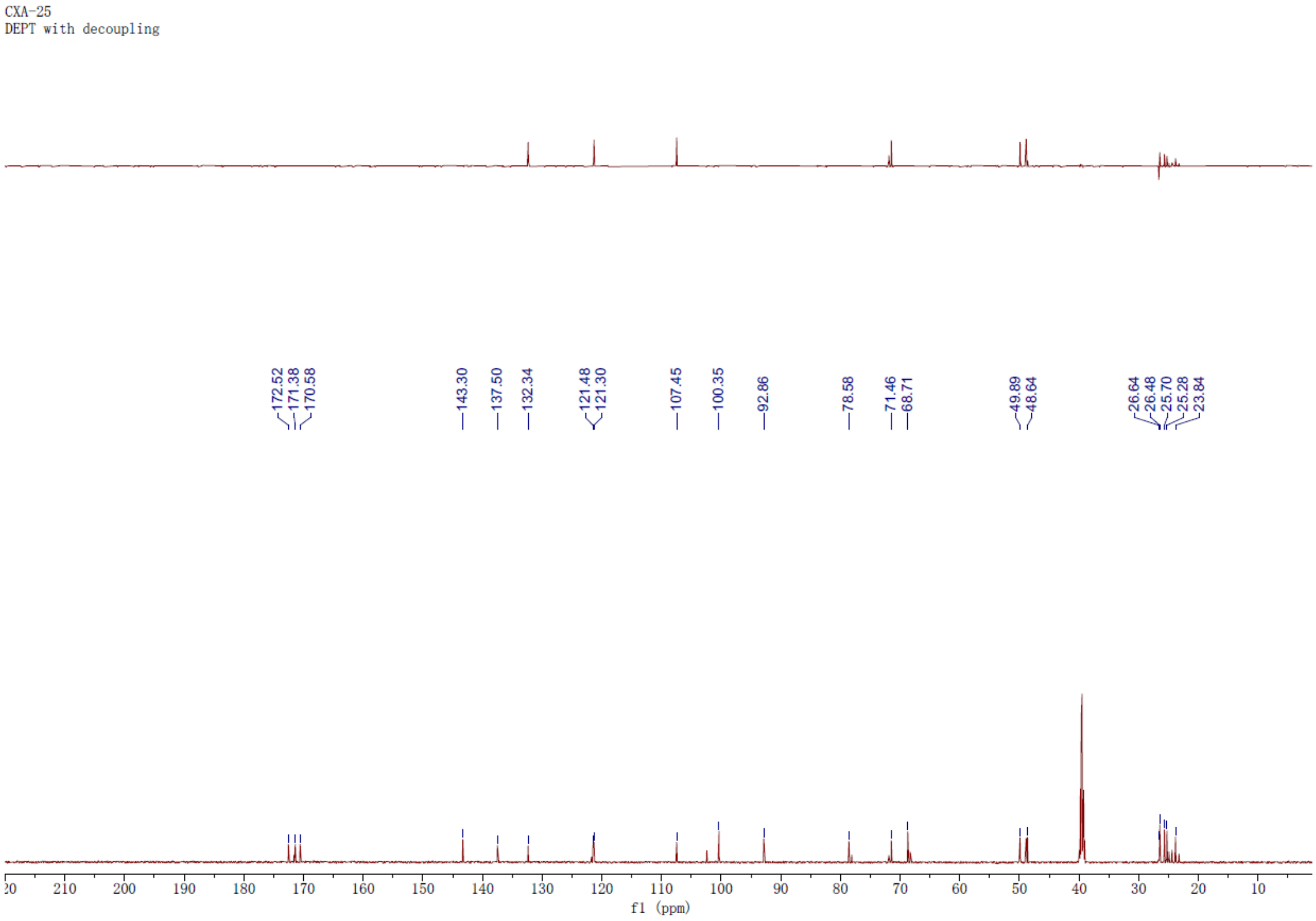

**Figure S24.** $^{13}C$ NMR (151 MHz) spectrum of compound **6** in DMSO-$d_6$.

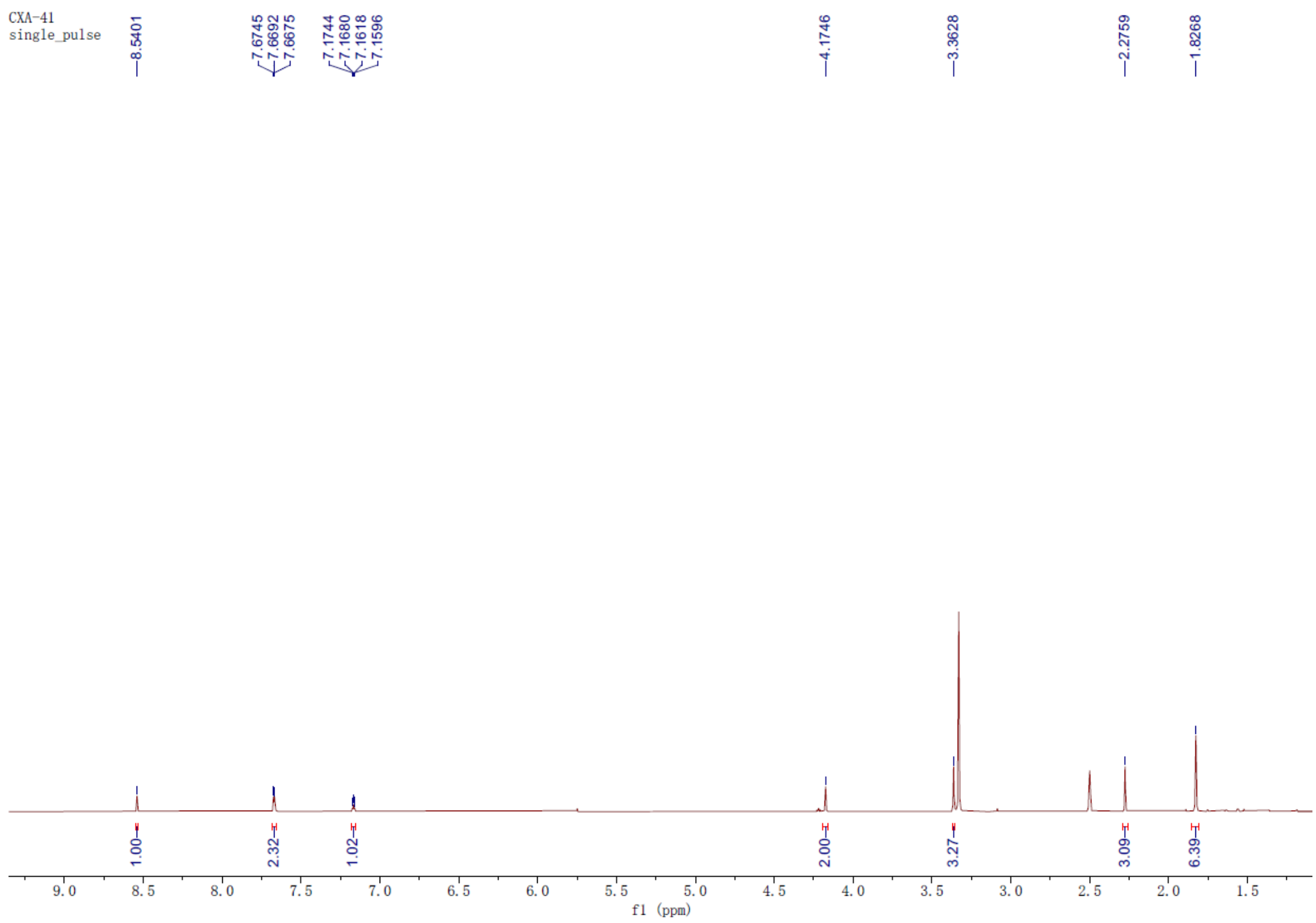


**Figure S25.** $^{1}H$ NMR (600 MHz) spectrum of compound **7** in DMSO-*d*6.

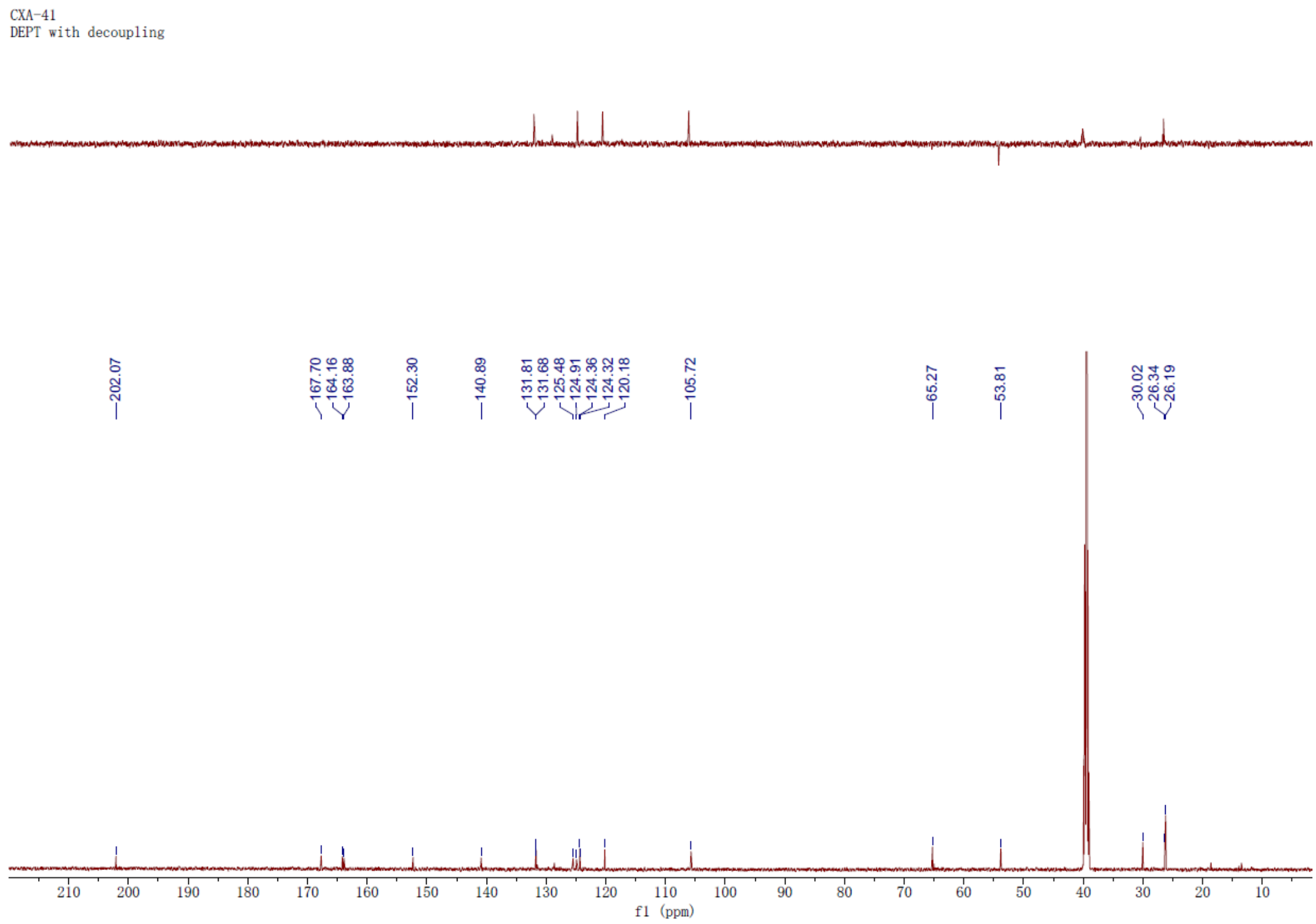


**Figure S26.** $^{13}C$ NMR (151 MHz) spectrum of compound **7** in DMSO-*d*6.

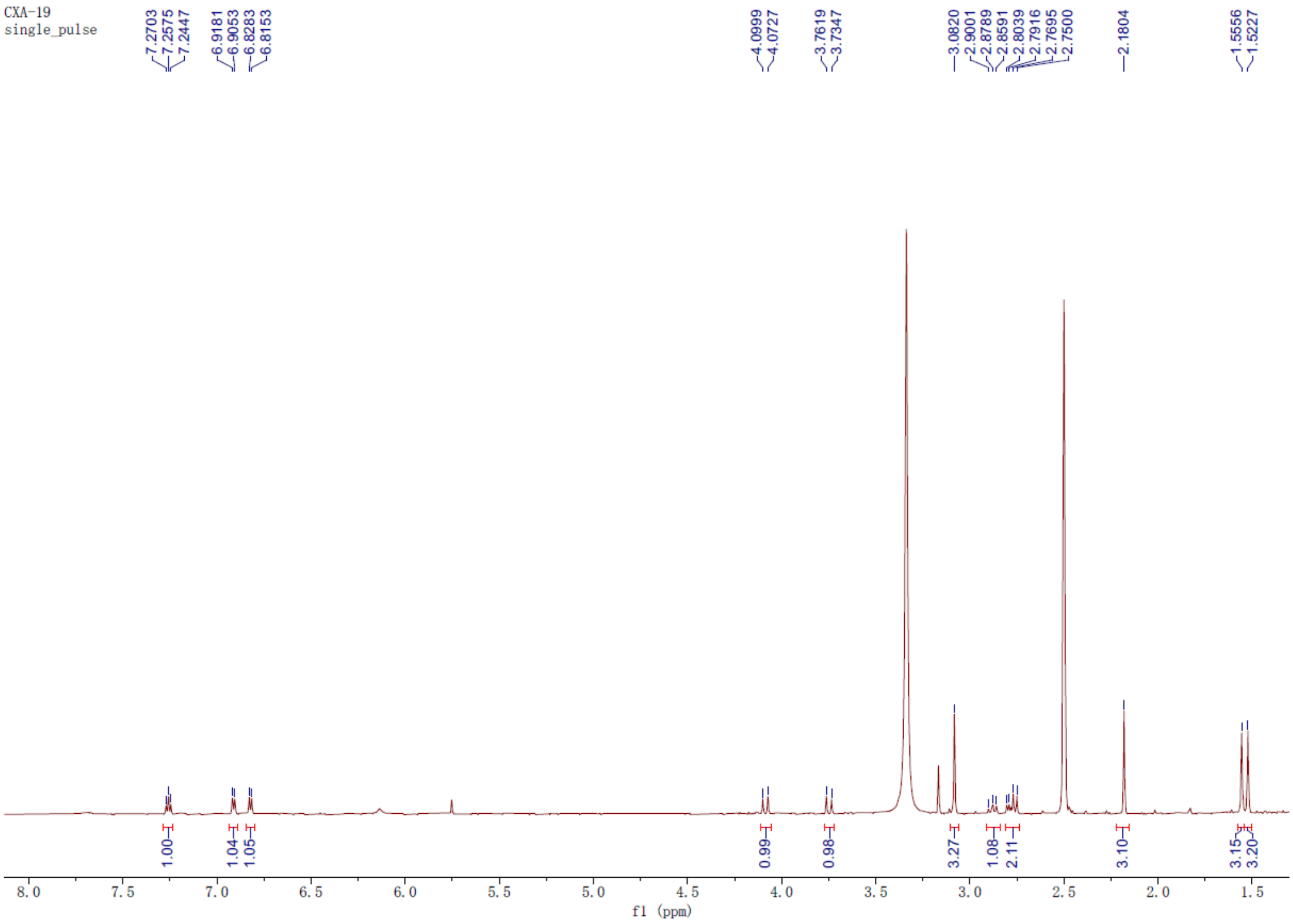


**Figure S27.** $^{1}$H NMR (600 MHz) spectrum of compound **8** in DMSO-*d*$_6$.

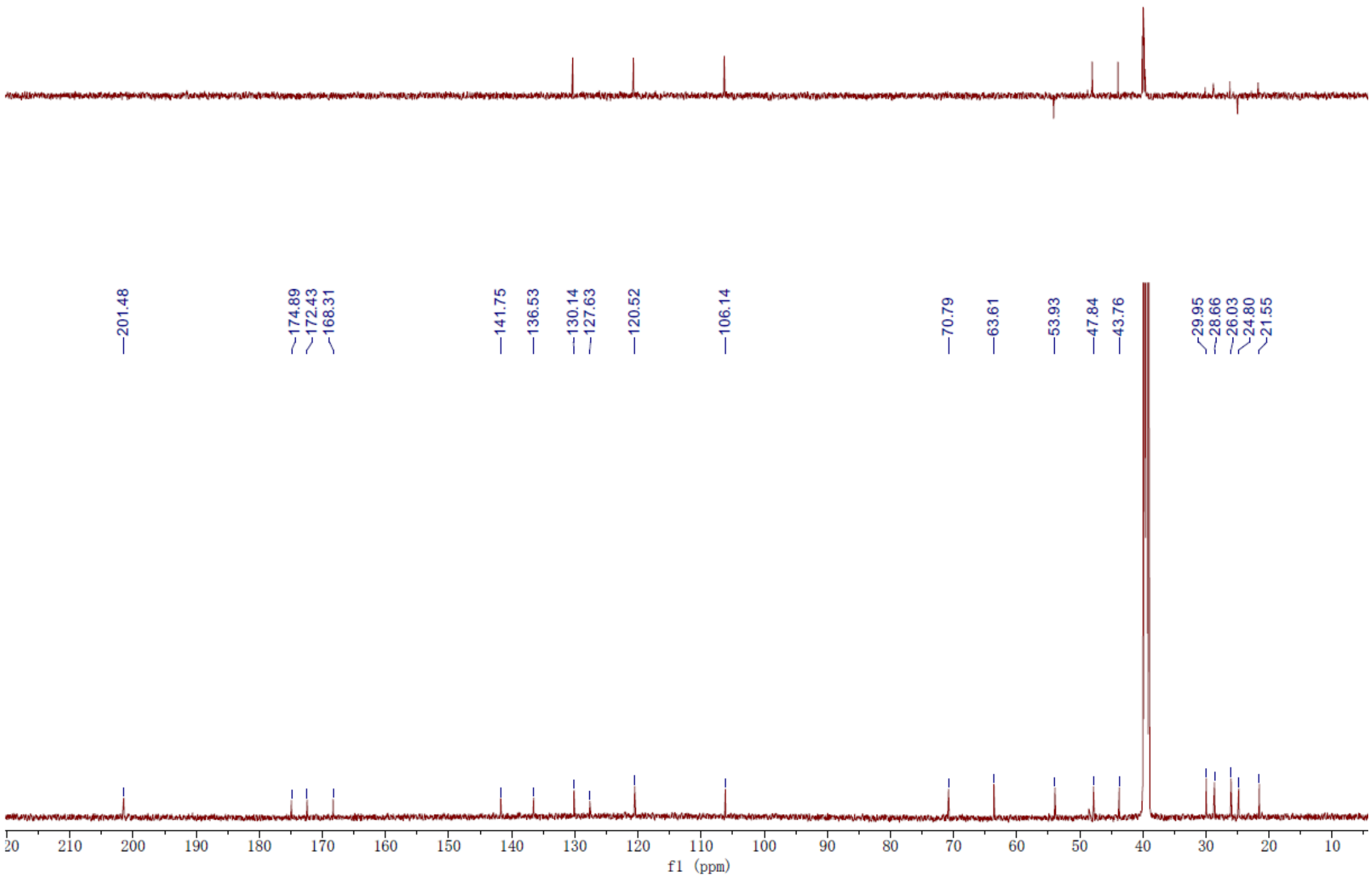


**Figure S28.** $^{13}$C NMR (151 MHz) spectrum of compound **8** in DMSO-*d*$_6$.

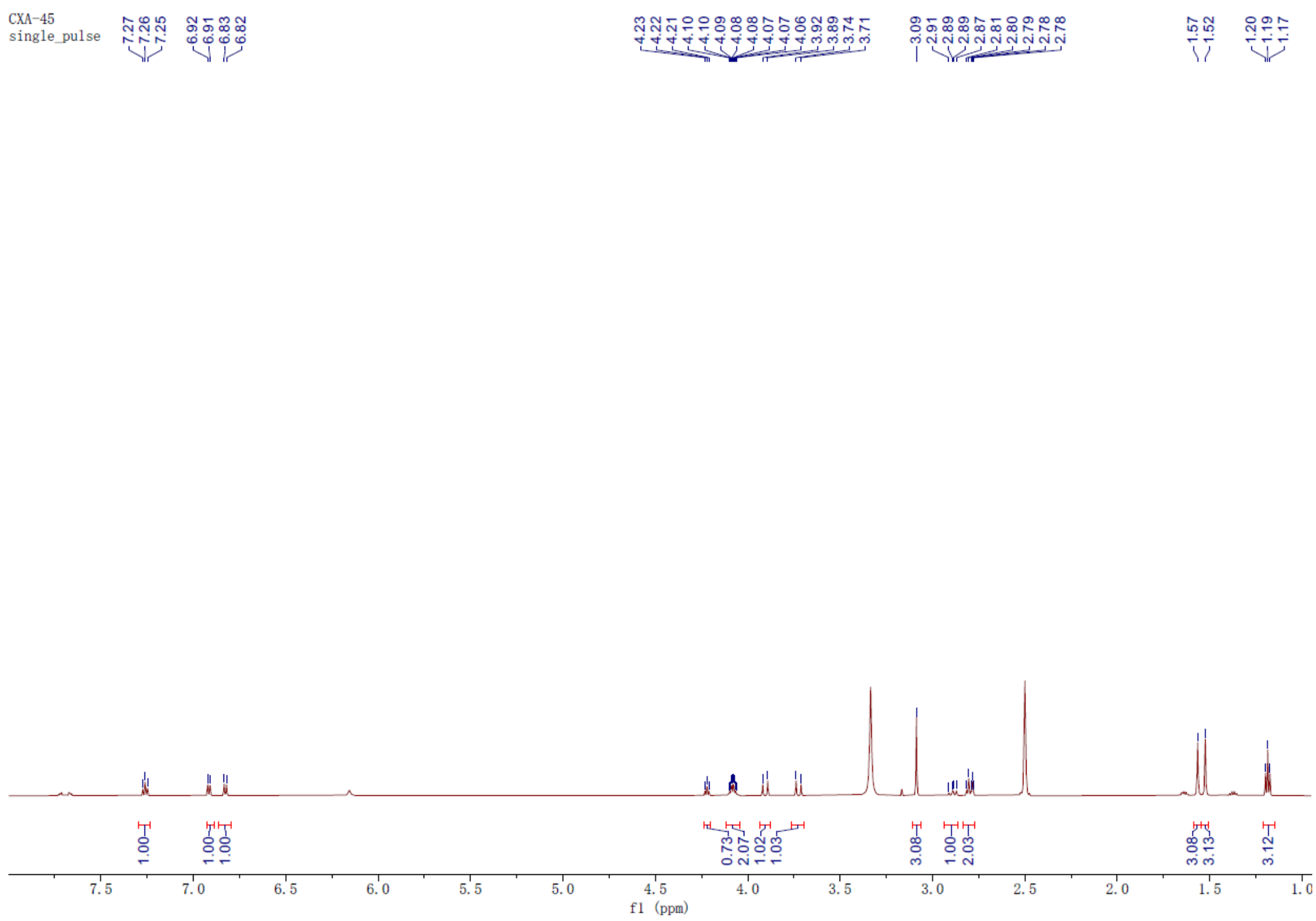

**Figure S29.** $^{1}$H NMR (600 MHz) spectrum of compound **9** in DMSO-$d_6$.

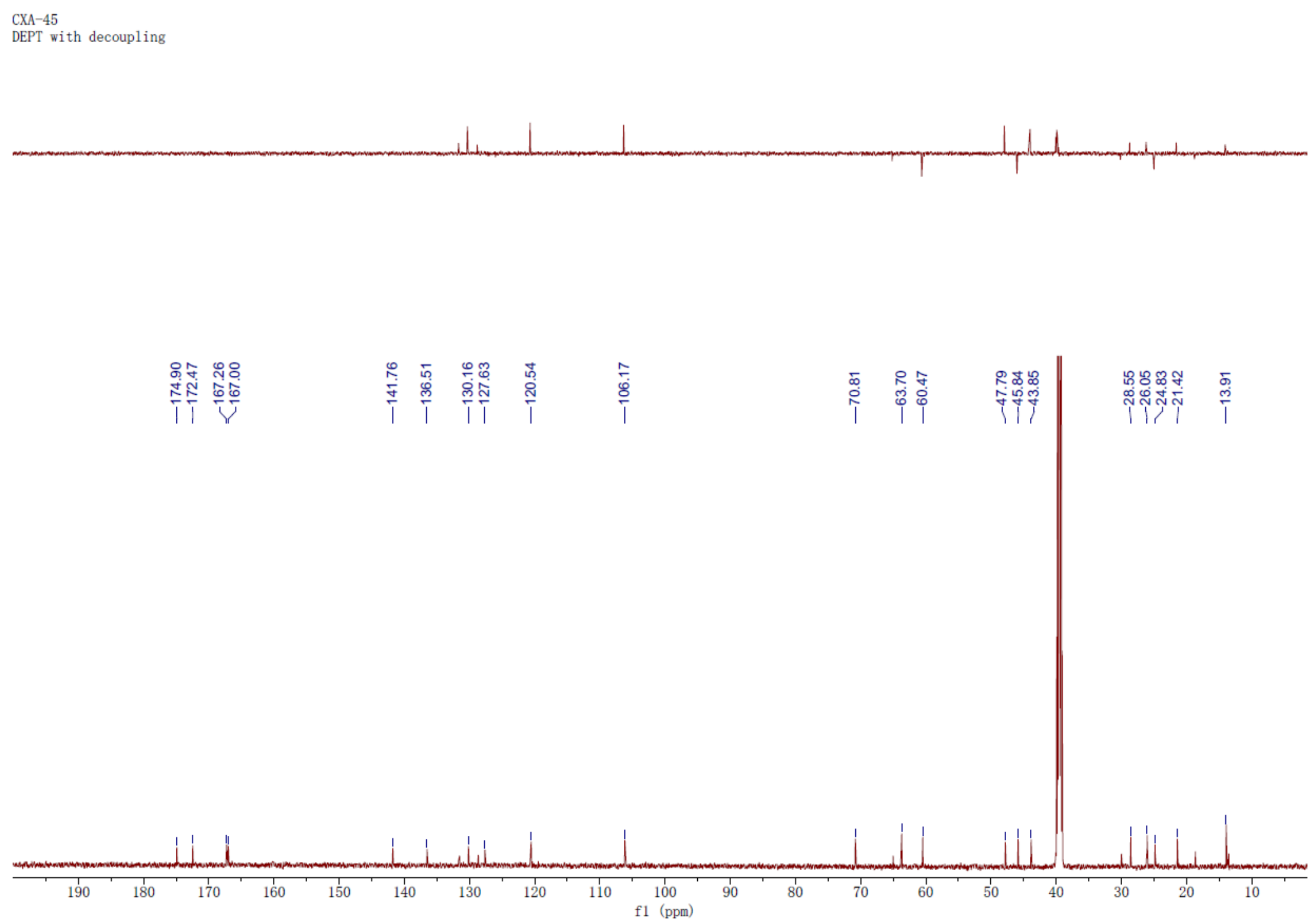

**Figure S30.** $^{13}$C NMR (151 MHz) spectrum of compound **9** in DMSO-$d_6$.

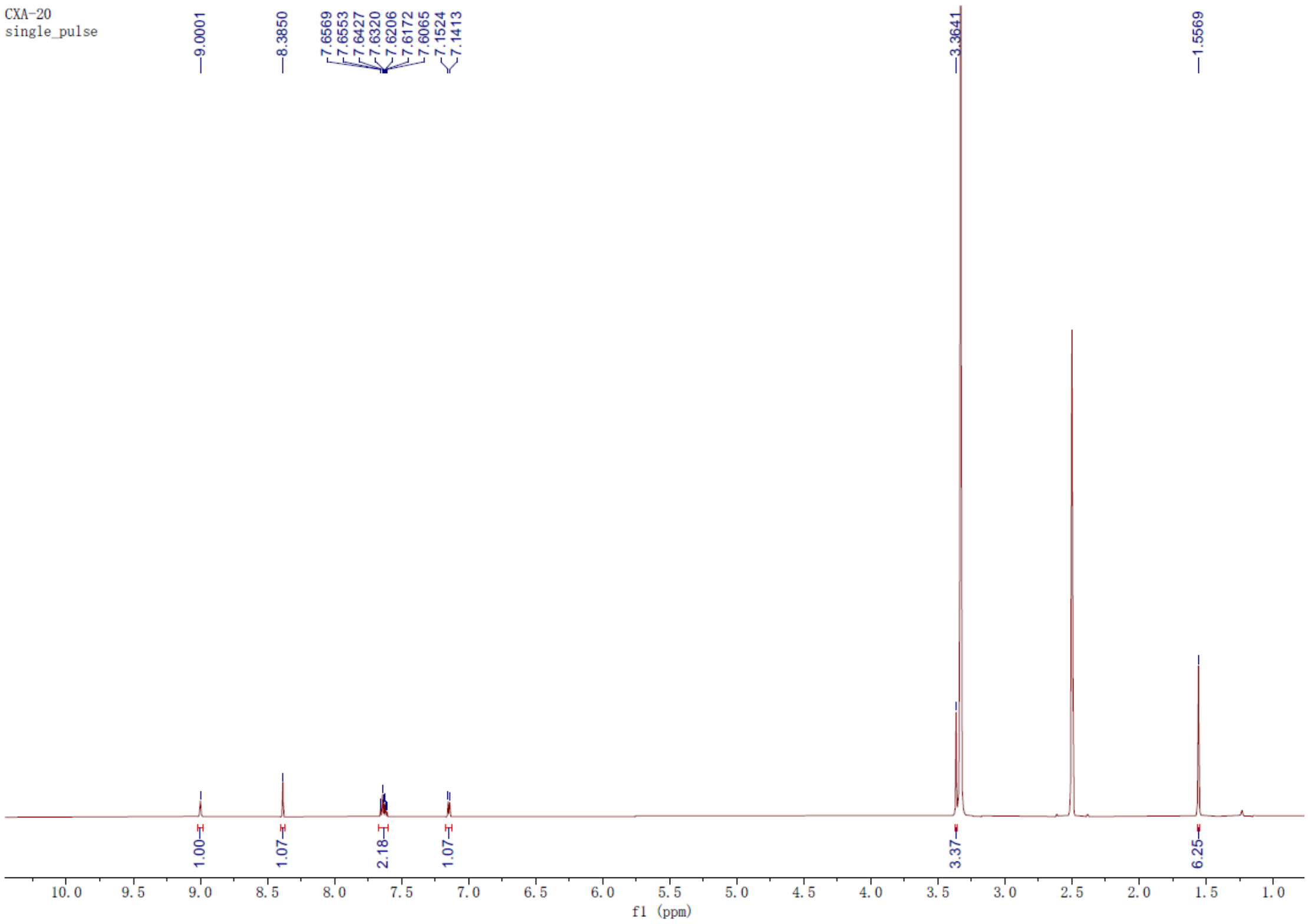


**Figure S31.** $^{1}$H NMR (600 MHz) spectrum of compound **10** in DMSO-$d_6$.

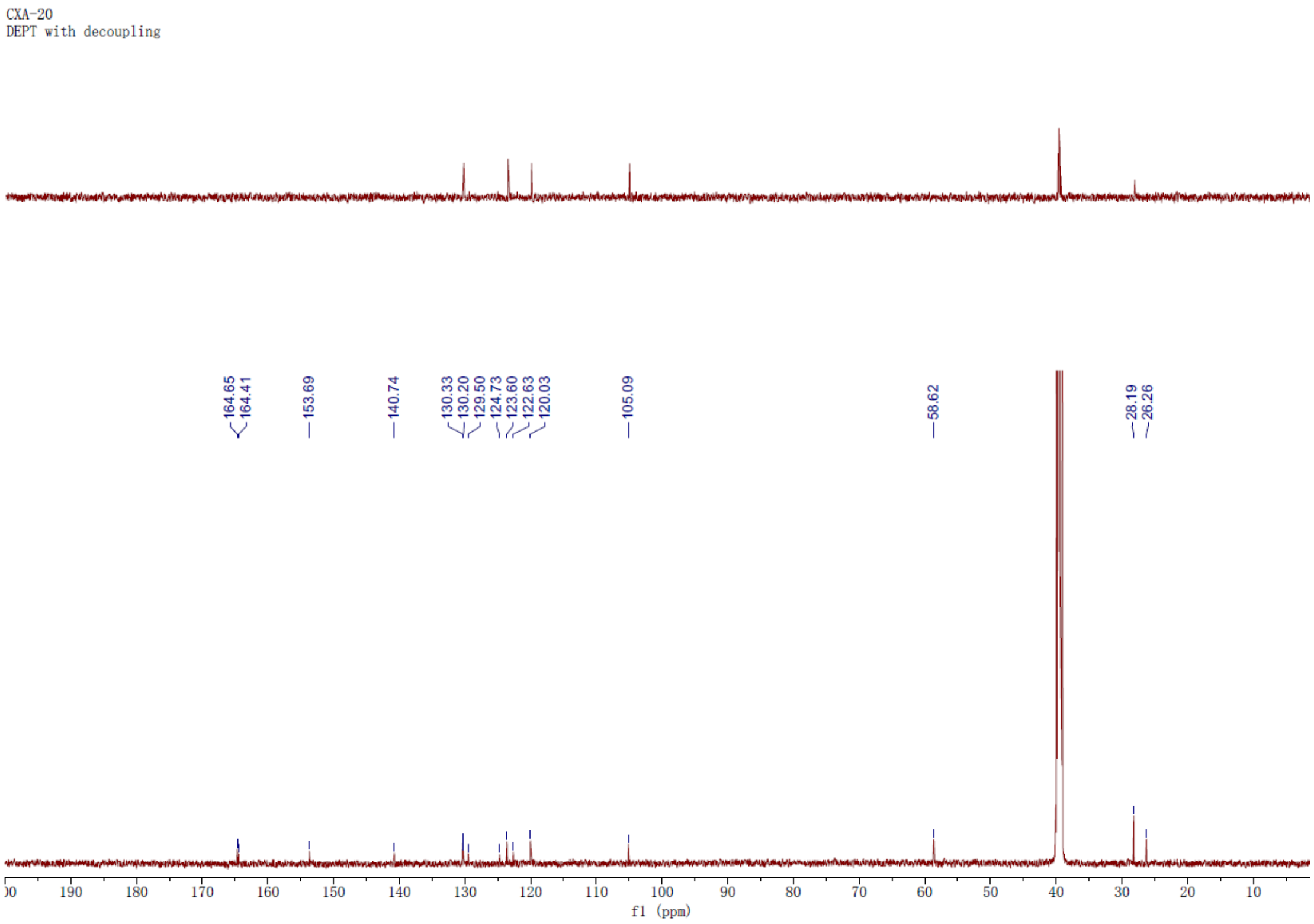


**Figure S32.** $^{13}$C NMR (151 MHz) spectrum of compound **10** in DMSO-$d_6$.

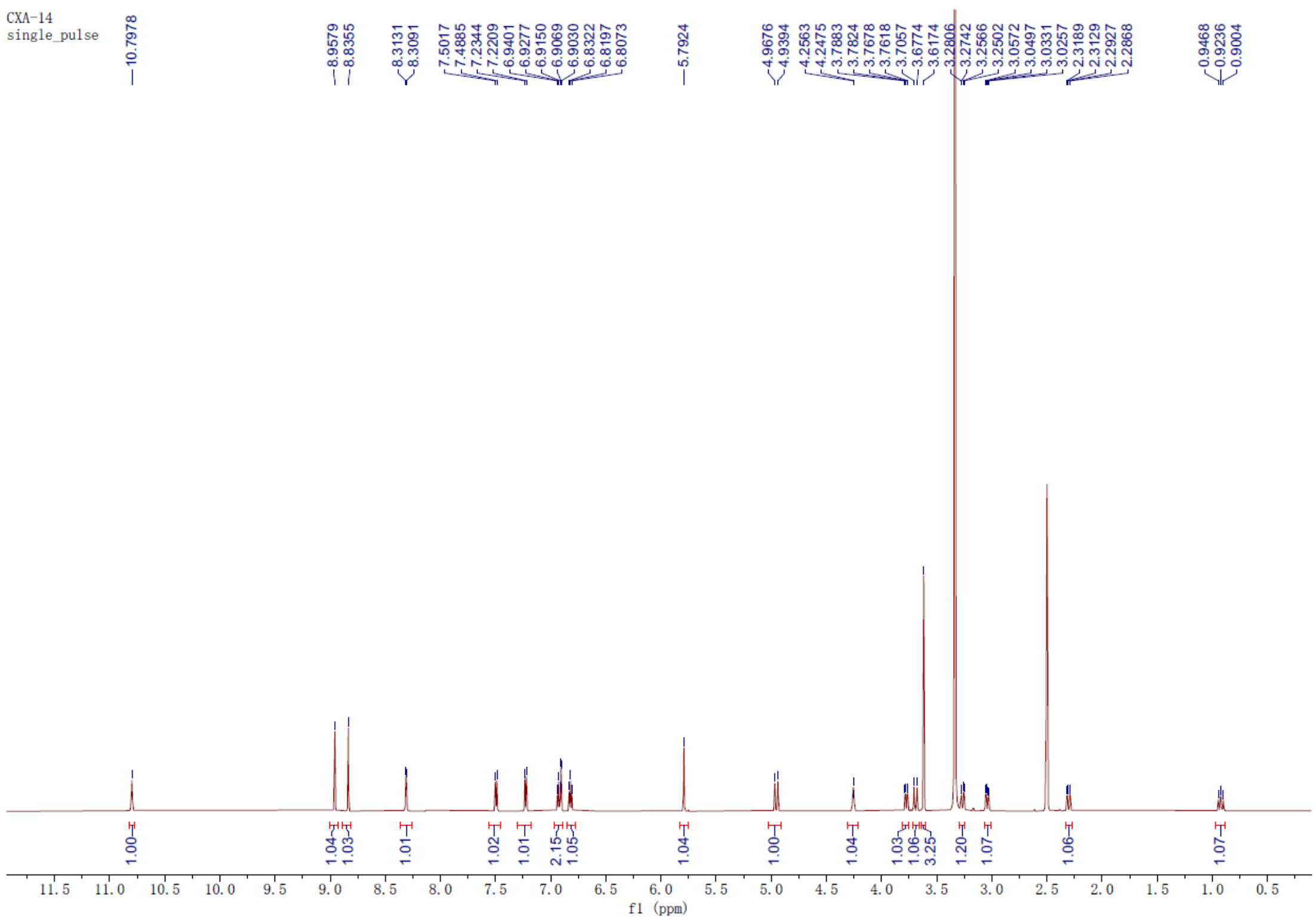


**Figure S33.** $^{1}H$ NMR (600 MHz) spectrum of compound **11** in DMSO-$d_6$.

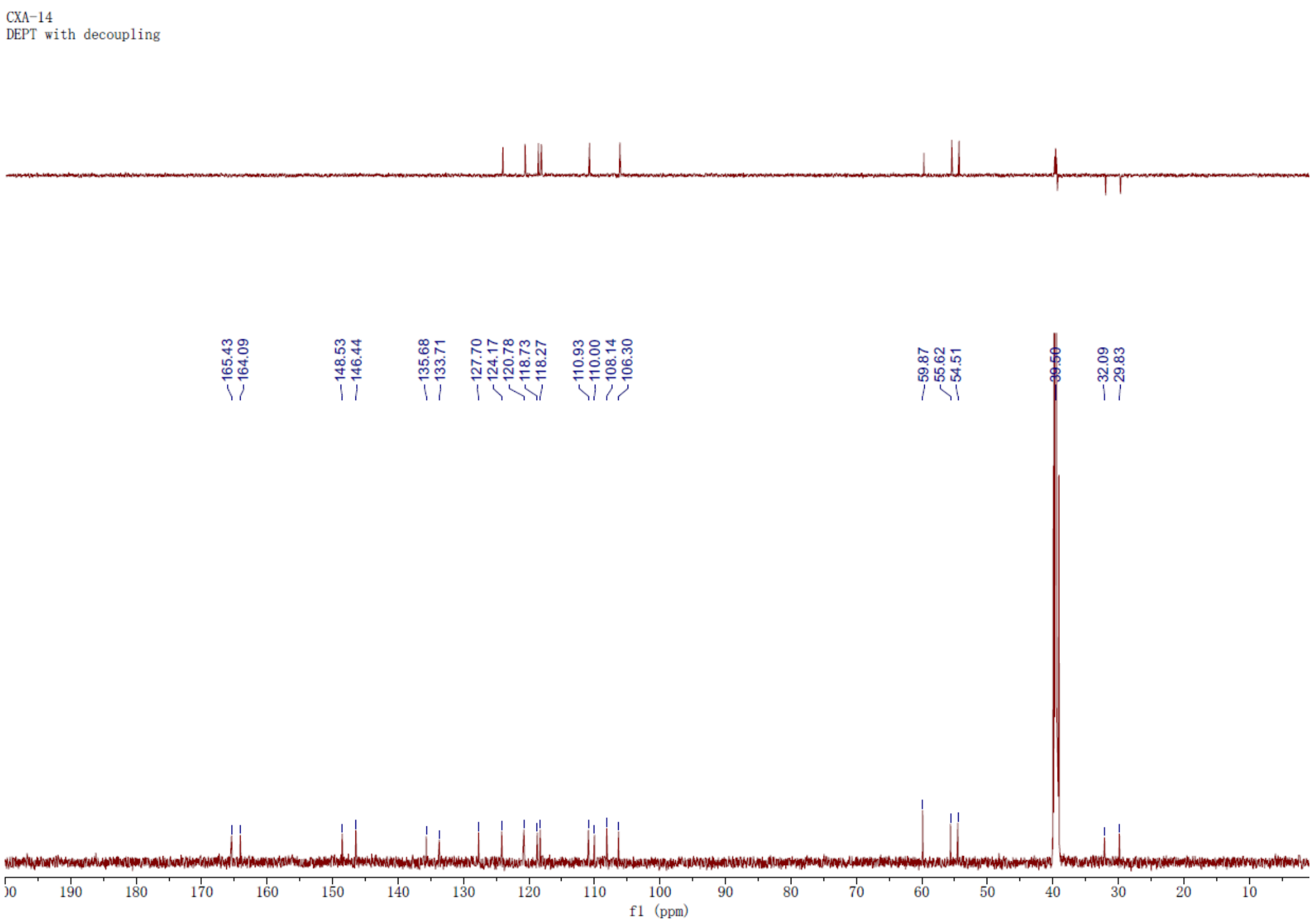


**Figure S34.** $^{13}C$ NMR (151 MHz) spectrum of compound **11** in DMSO-$d_6$.

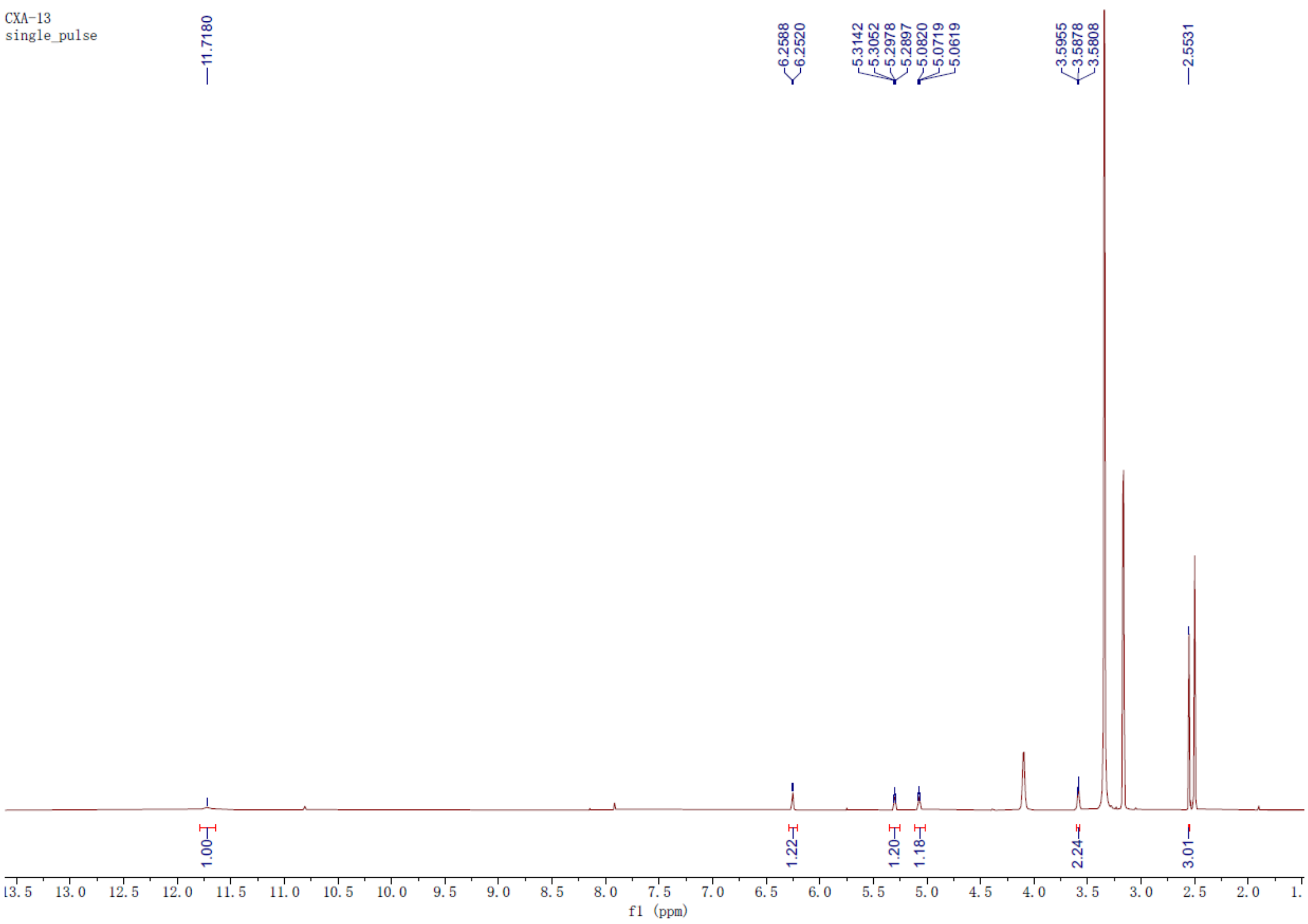


**Figure S35.** $^{1}H$ NMR (600 MHz) spectrum of compound **12** in DMSO-$d_6$.

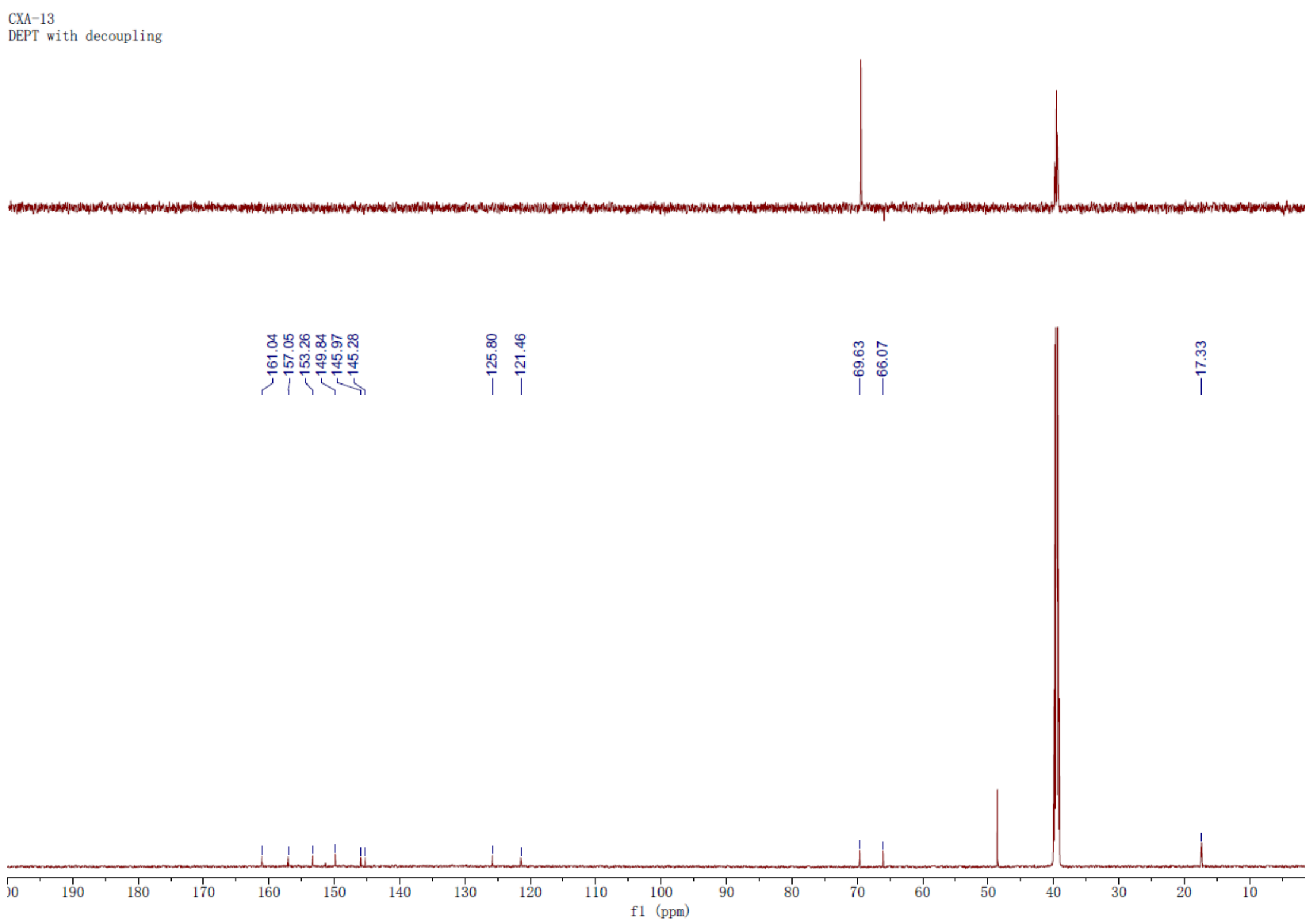


**Figure S36.** $^{13}C$ NMR (151 MHz) spectrum of compound **12** in DMSO-$d_6$.

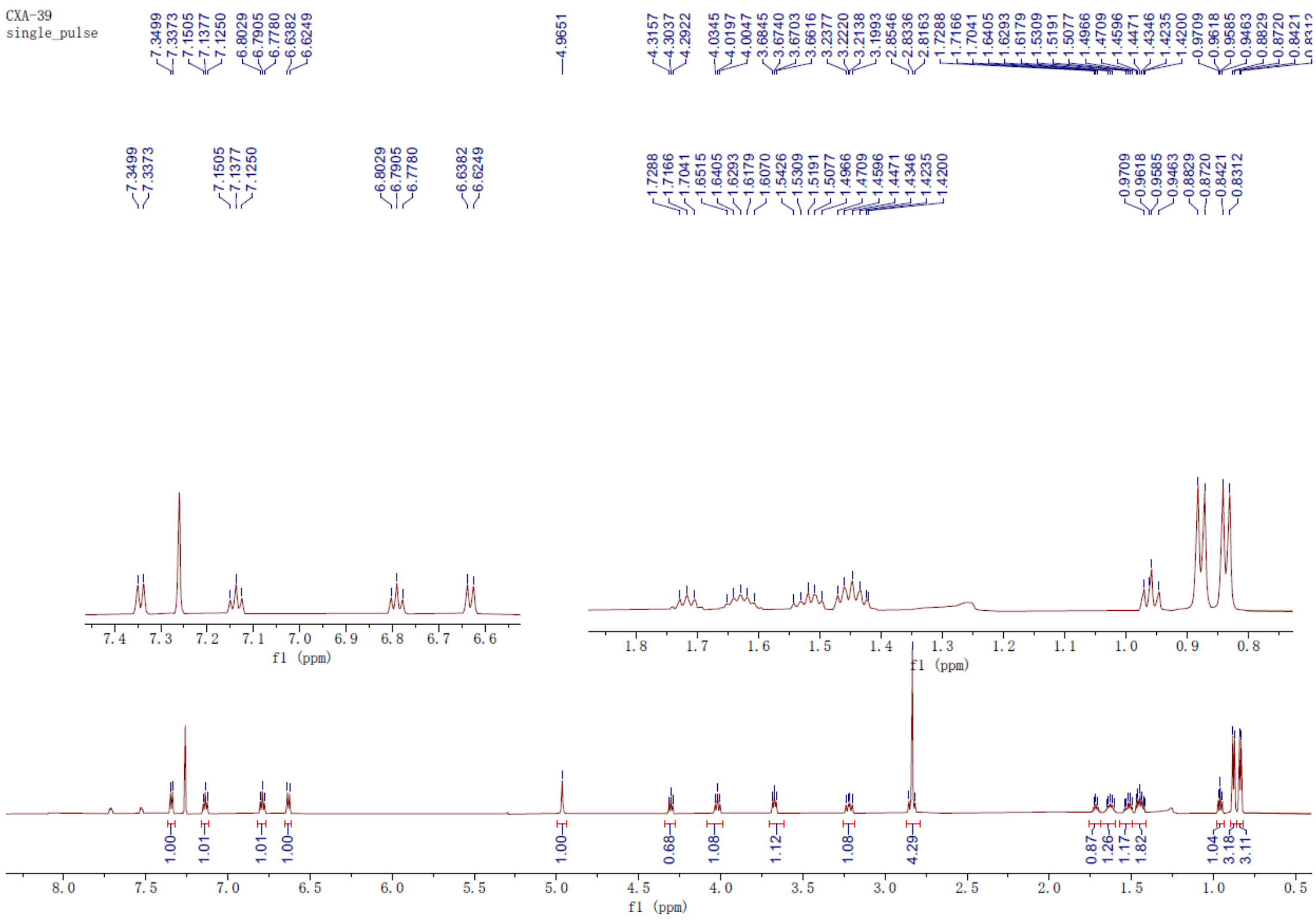

**Figure S37.** $^{1}H$ NMR (600 MHz) spectrum of compound **13** in $CDCl_3$.

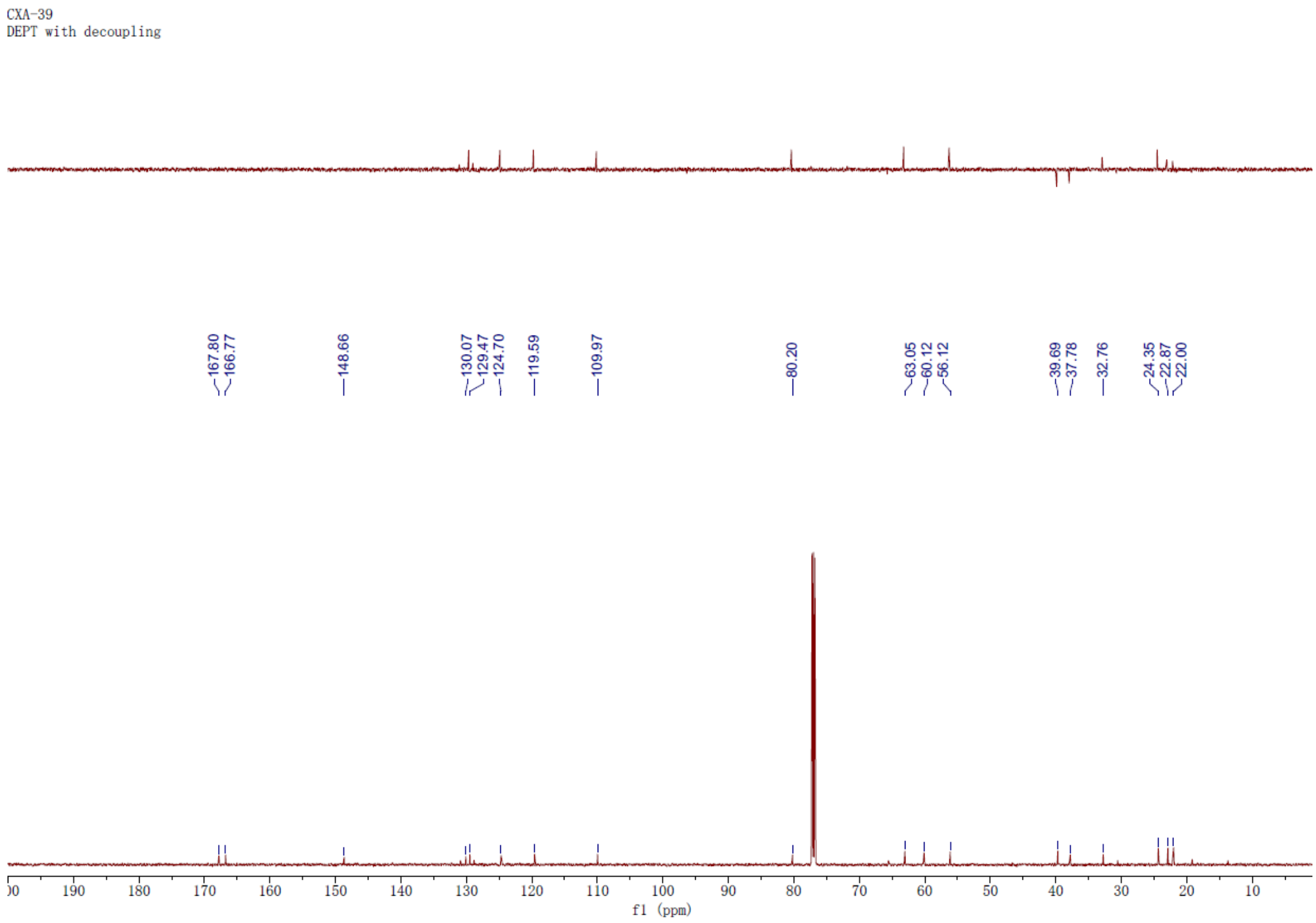

**Figure S38.** $^{13}C$ NMR (151 MHz) spectrum of compound **13** in $CDCl_3$.

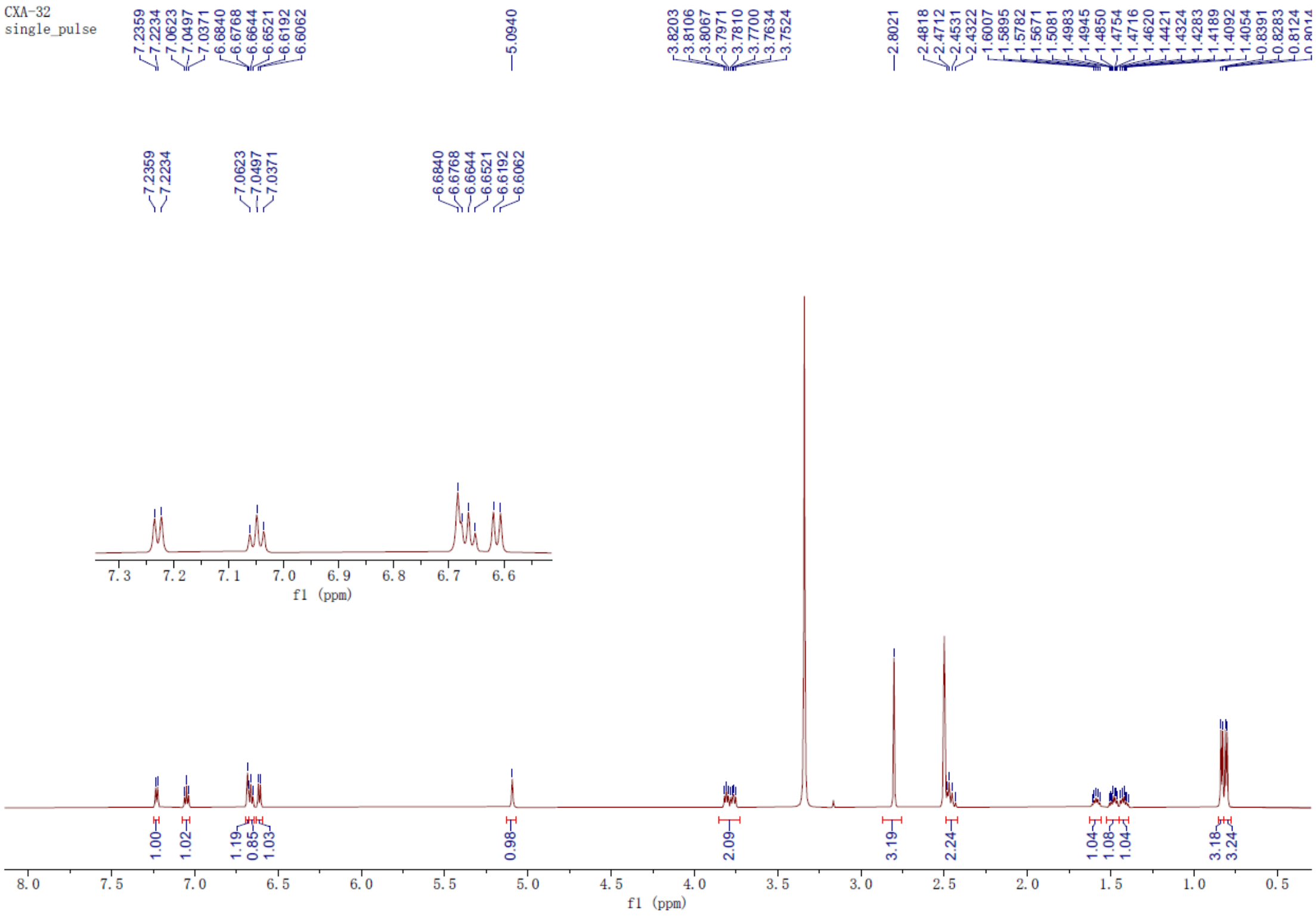


**Figure S39.** $^1$H NMR (600 MHz) spectrum of compound **14** in DMSO-*d*$_6$.

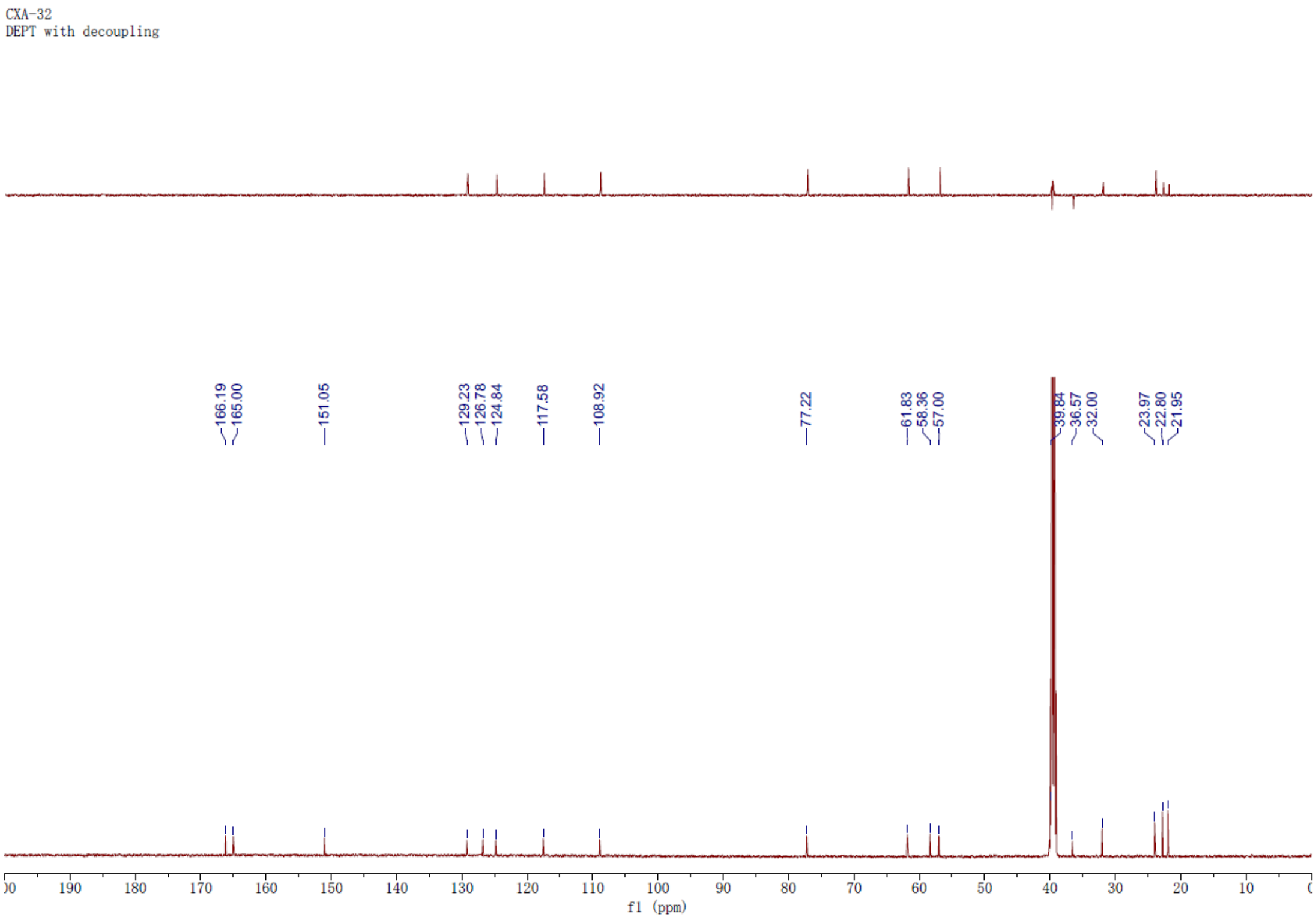


**Figure S40.** $^{13}$C NMR (151 MHz) spectrum of compound **14** in DMSO-*d*$_6$.

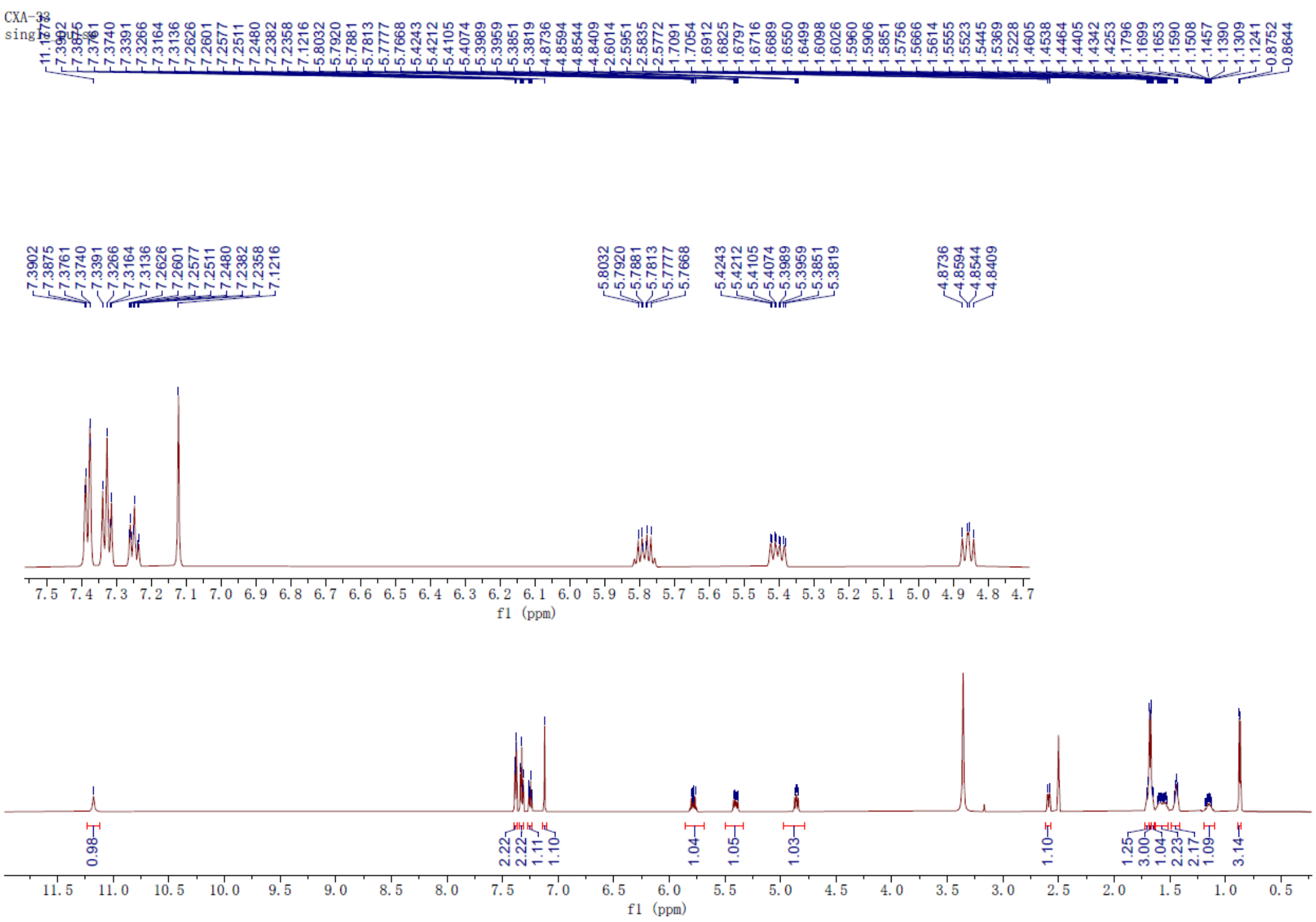

**Figure S41.** $^{1}$H NMR (600 MHz) spectrum of compound **15** in DMSO-$d_6$.

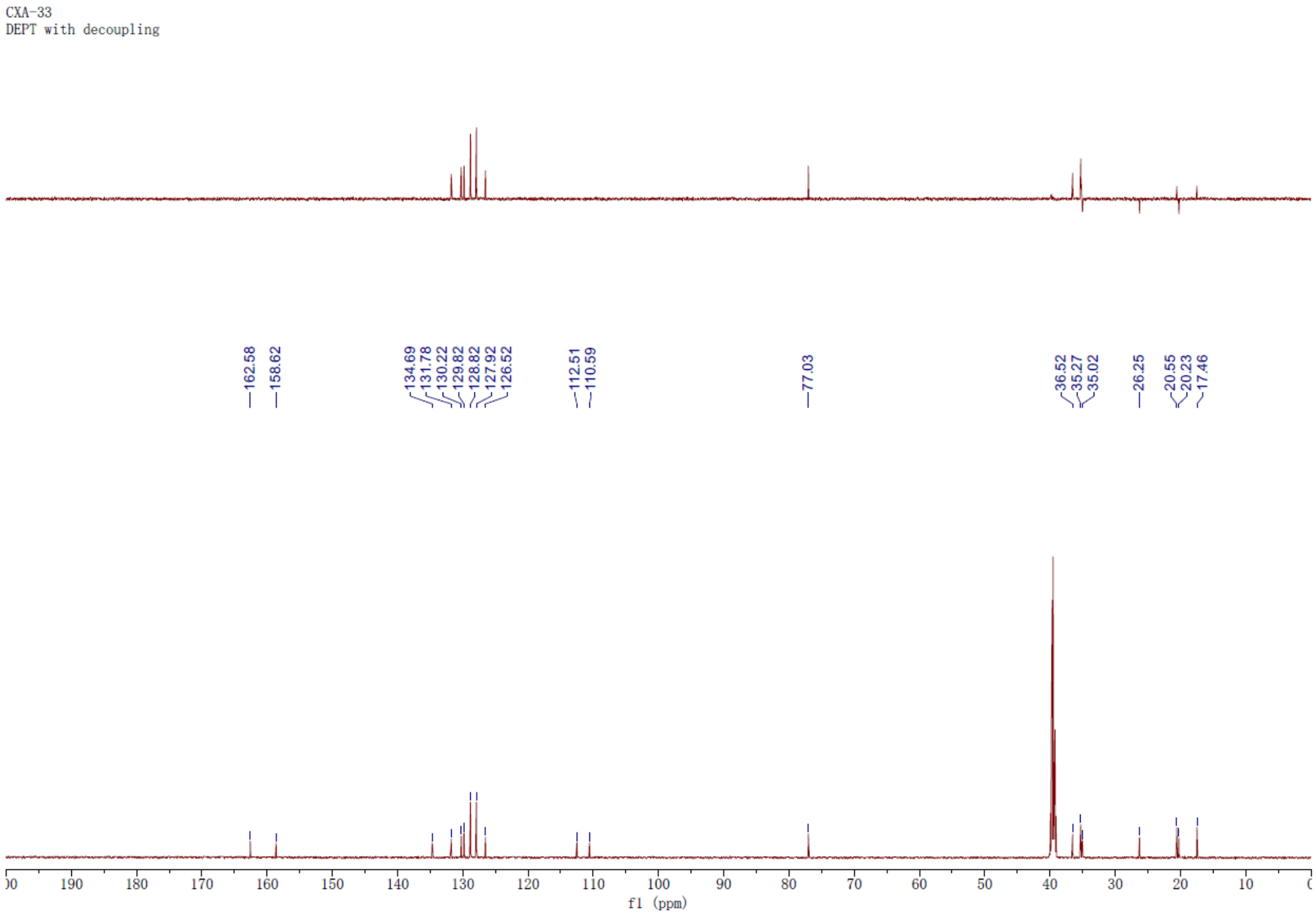

**Figure S42.** $^{13}$C NMR (151 MHz) spectrum of compound **15** in DMSO-$d_6$.

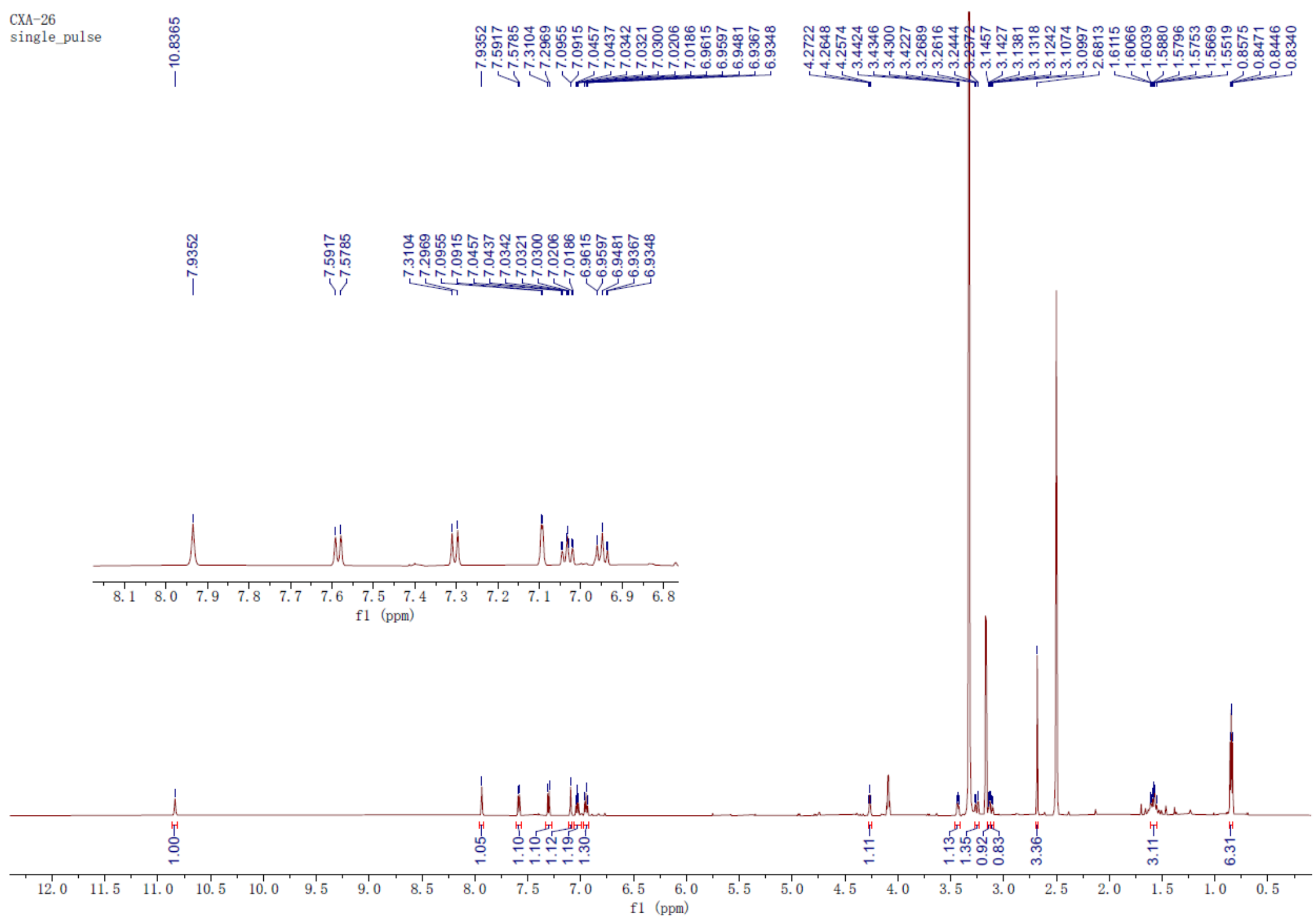

**Figure S43.** $^{1}$H NMR (600 MHz) spectrum of compound **16** in DMSO-$d_6$.

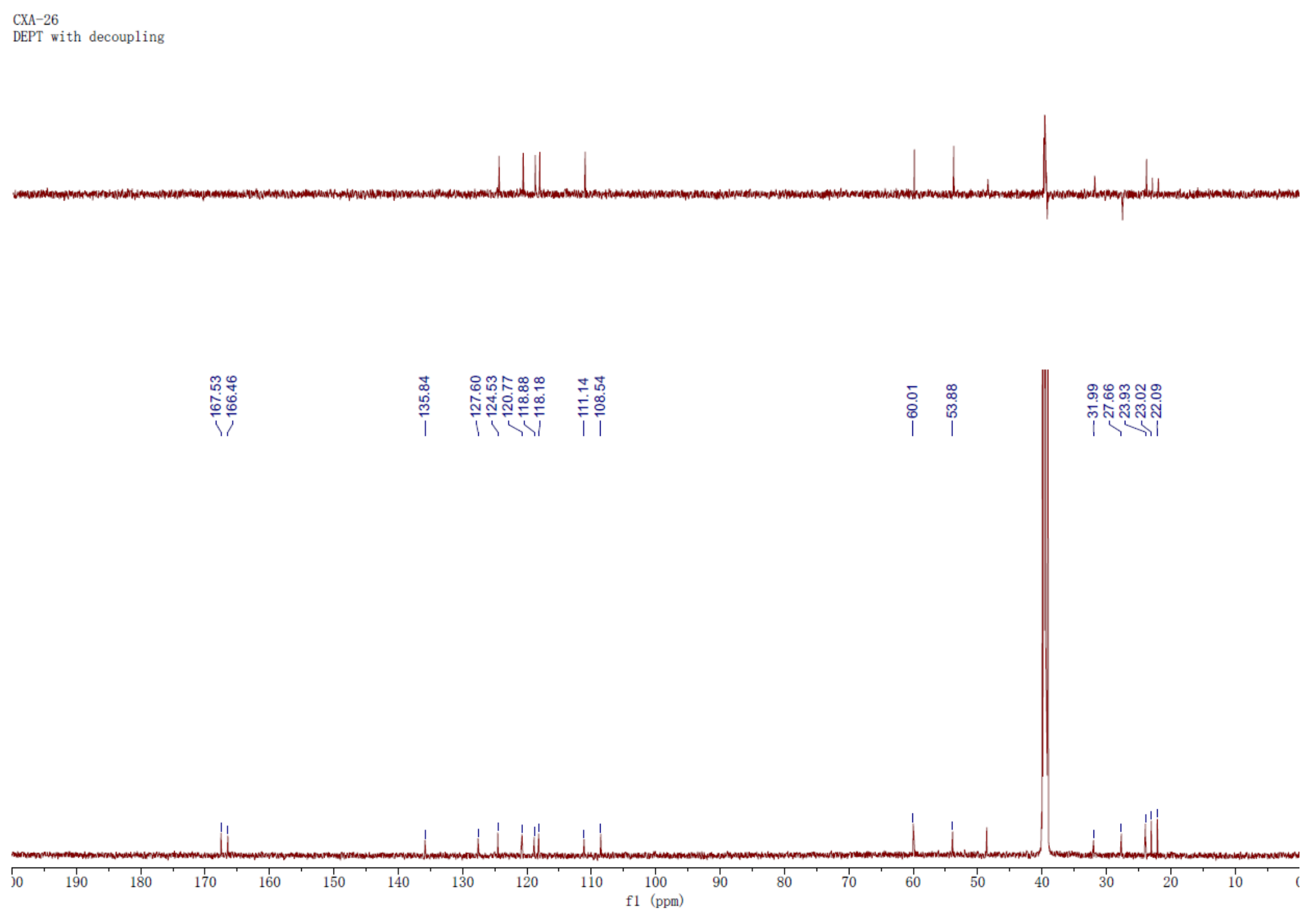

**Figure S44.** $^{13}$C NMR (151 MHz) spectrum of compound **16** in DMSO-$d_6$.

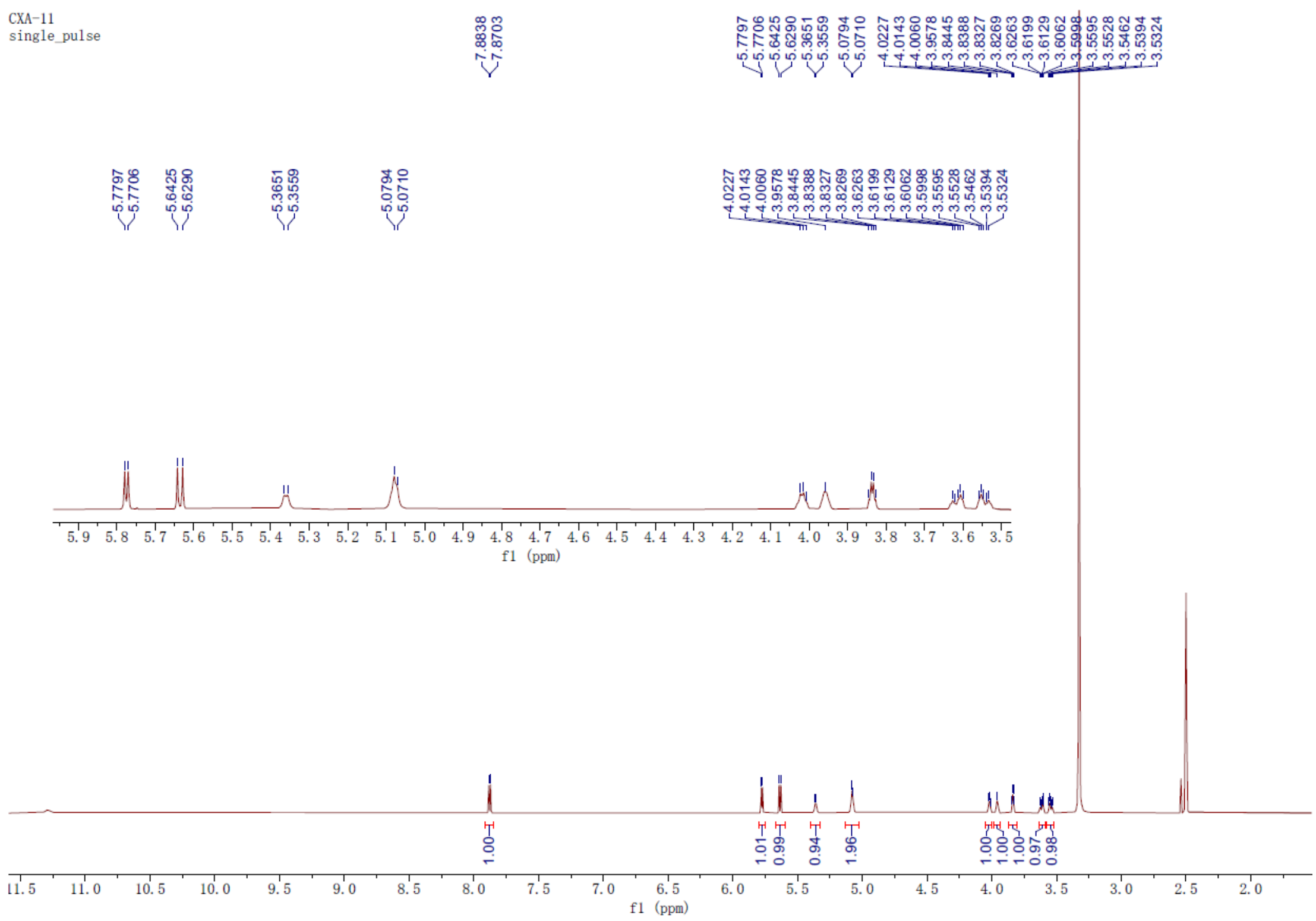

**Figure S45.** $^{1}$H NMR (600 MHz) spectrum of compound **17** in DMSO-$d_6$.

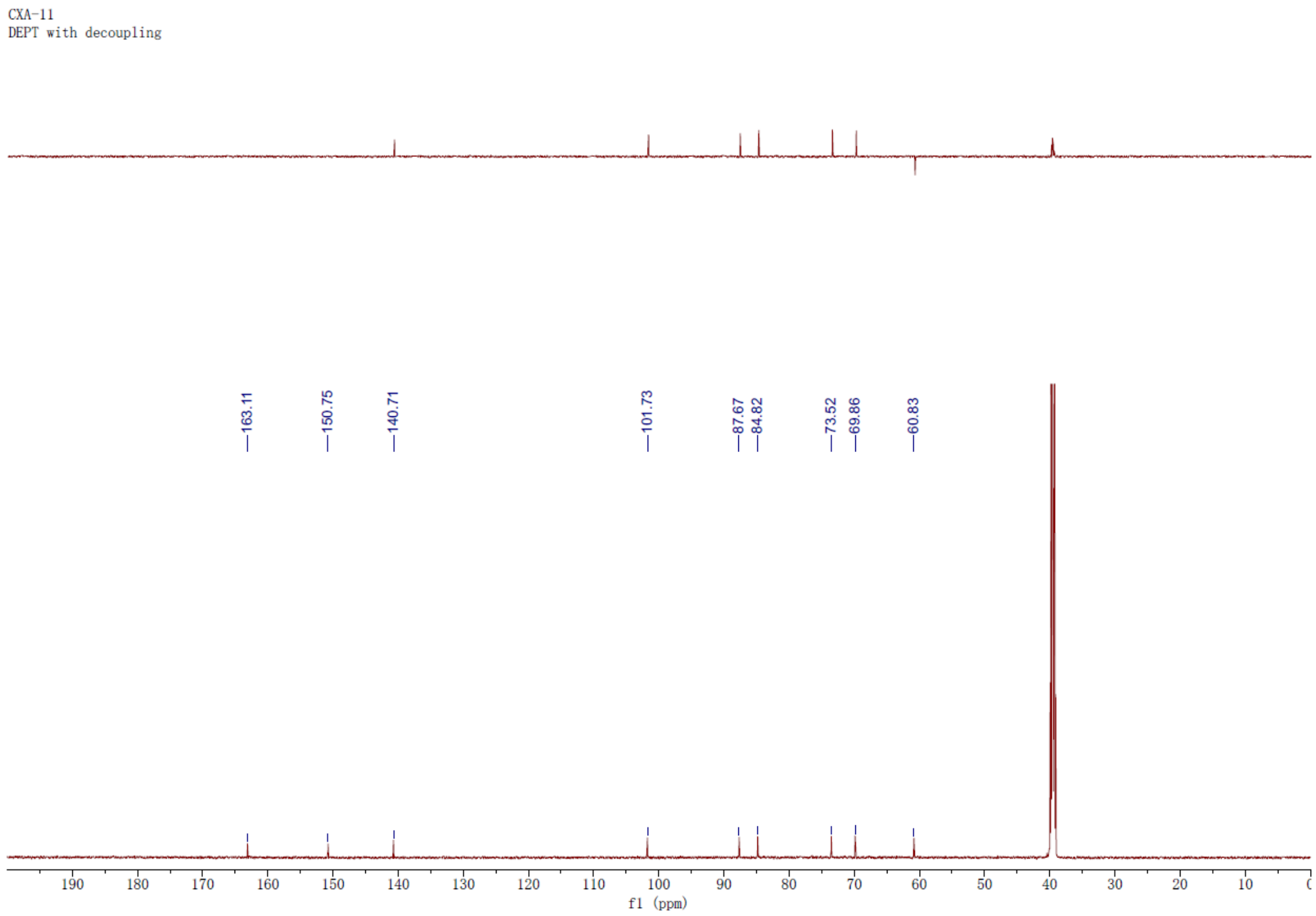

**Figure S46.** $^{13}$C NMR (151 MHz) spectrum of compound **17** in DMSO-$d_6$.

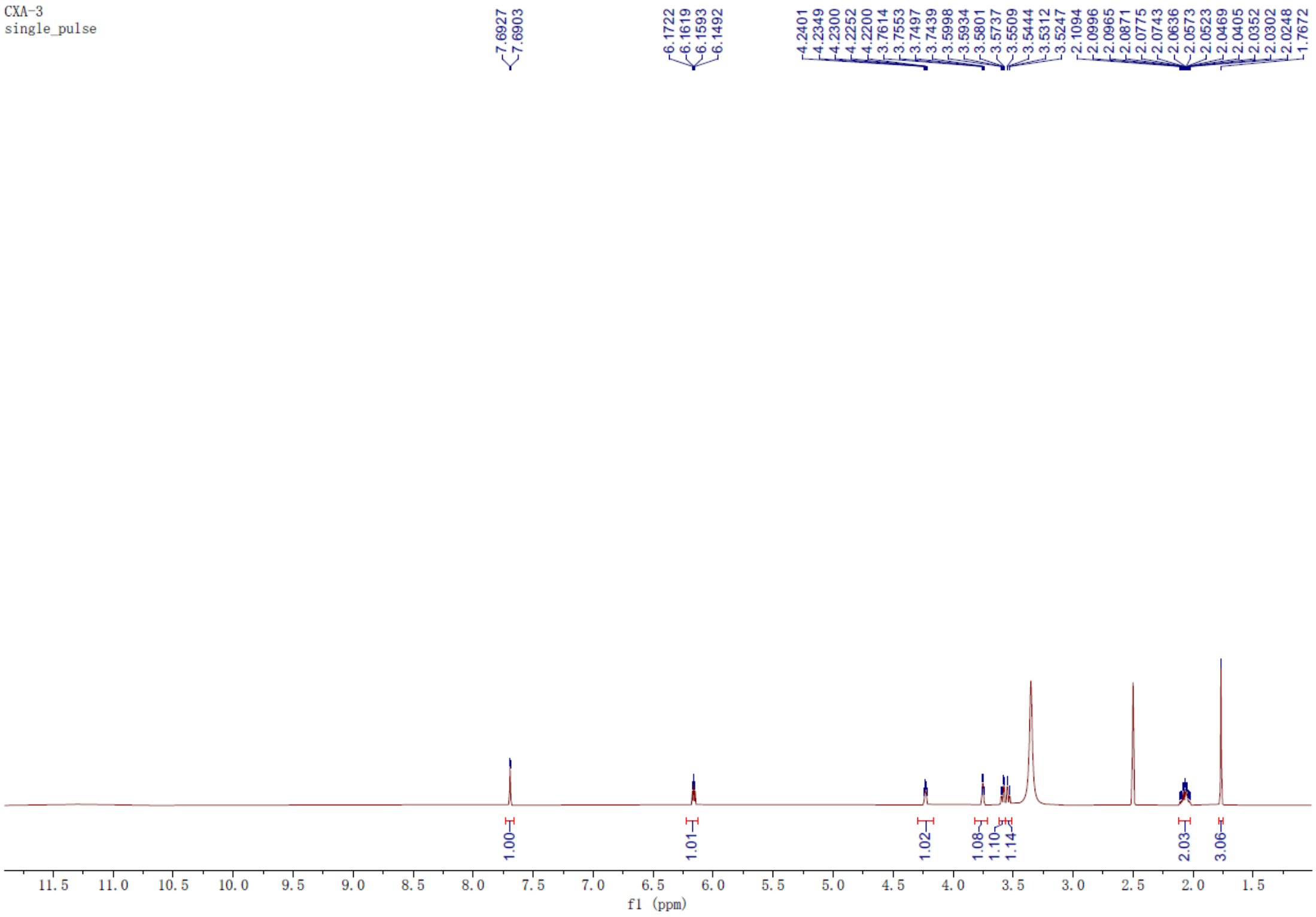

**Figure S47.** $^{1}H$ NMR (600 MHz) spectrum of compound **18** in DMSO-$d_6$.

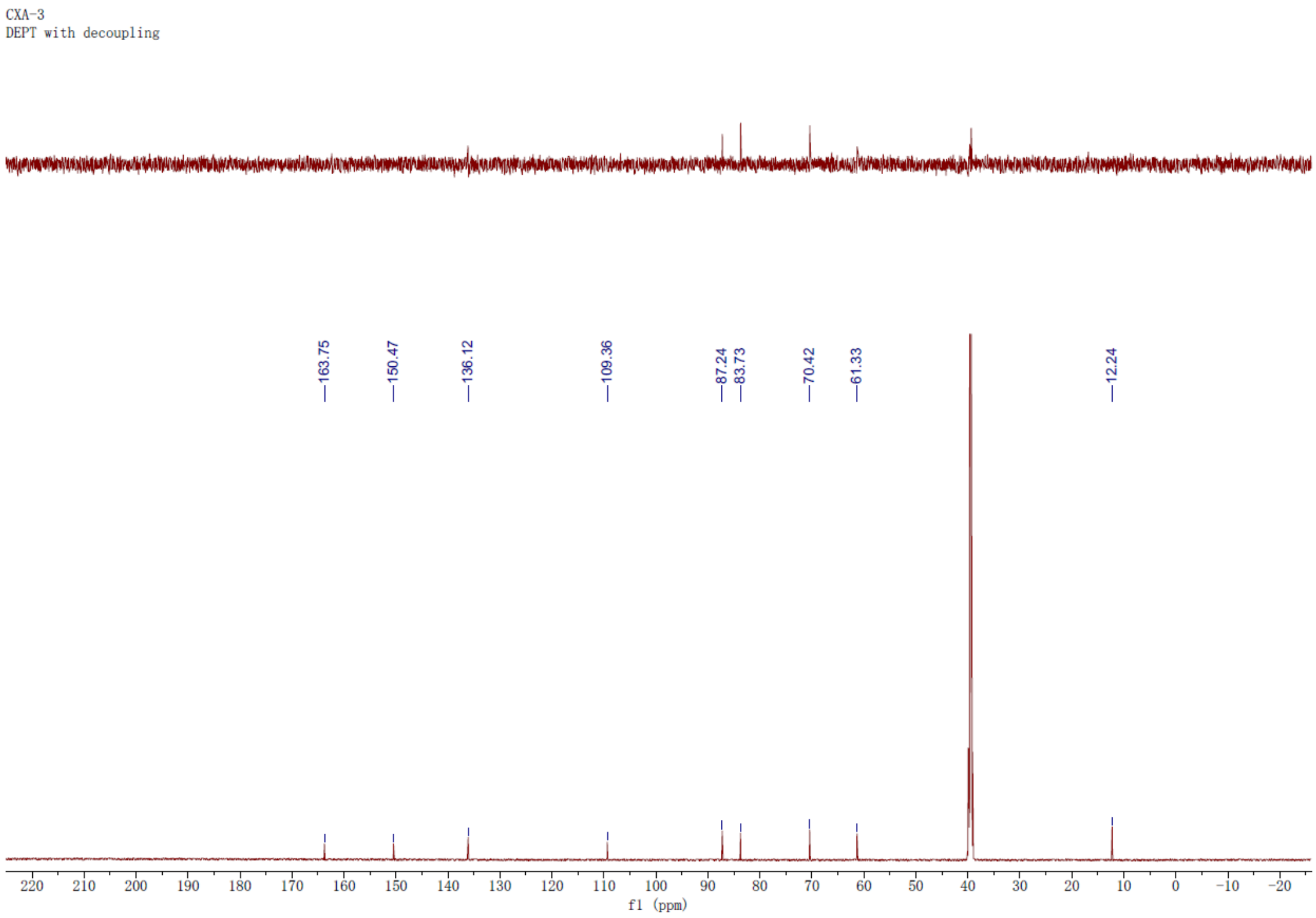

**Figure S48.** $^{13}C$ NMR (151 MHz) spectrum of compound **18** in DMSO-$d_6$.

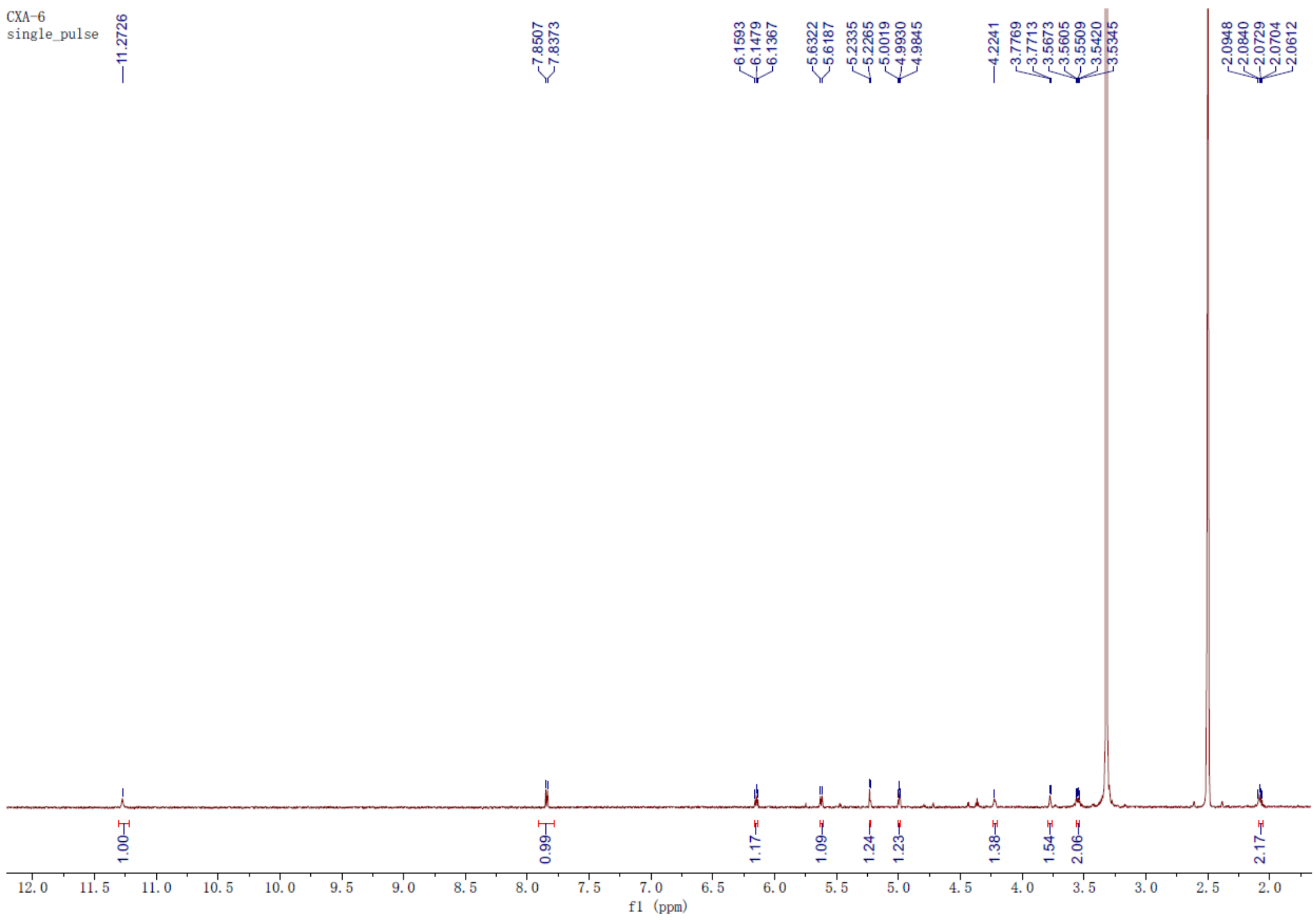


**Figure S49.** $^{1}H$ NMR (600 MHz) spectrum of compound **19** in DMSO-*d*6.

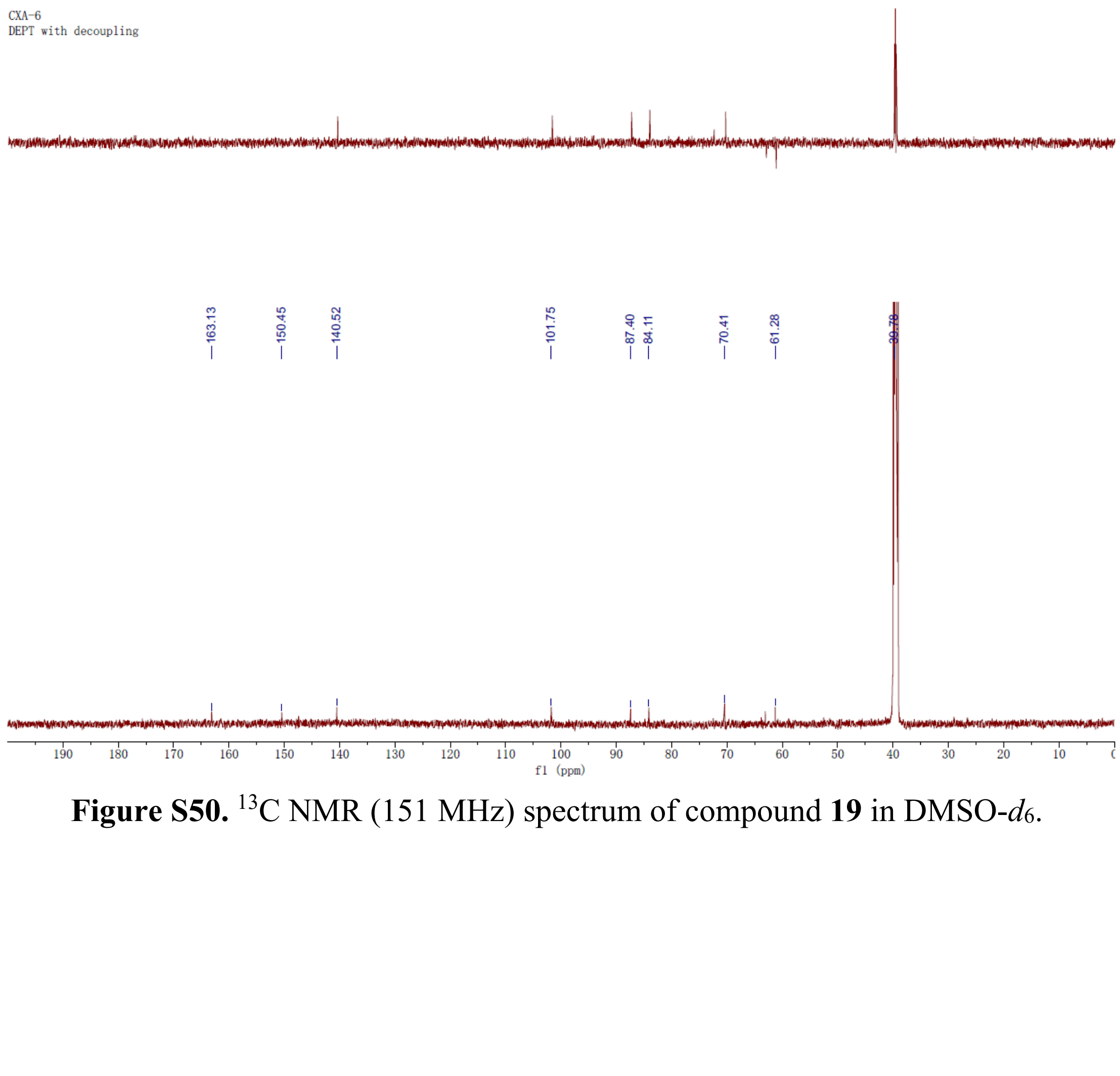


**Figure S50.** $^{13}C$ NMR (151 MHz) spectrum of compound **19** in DMSO-*d*6.

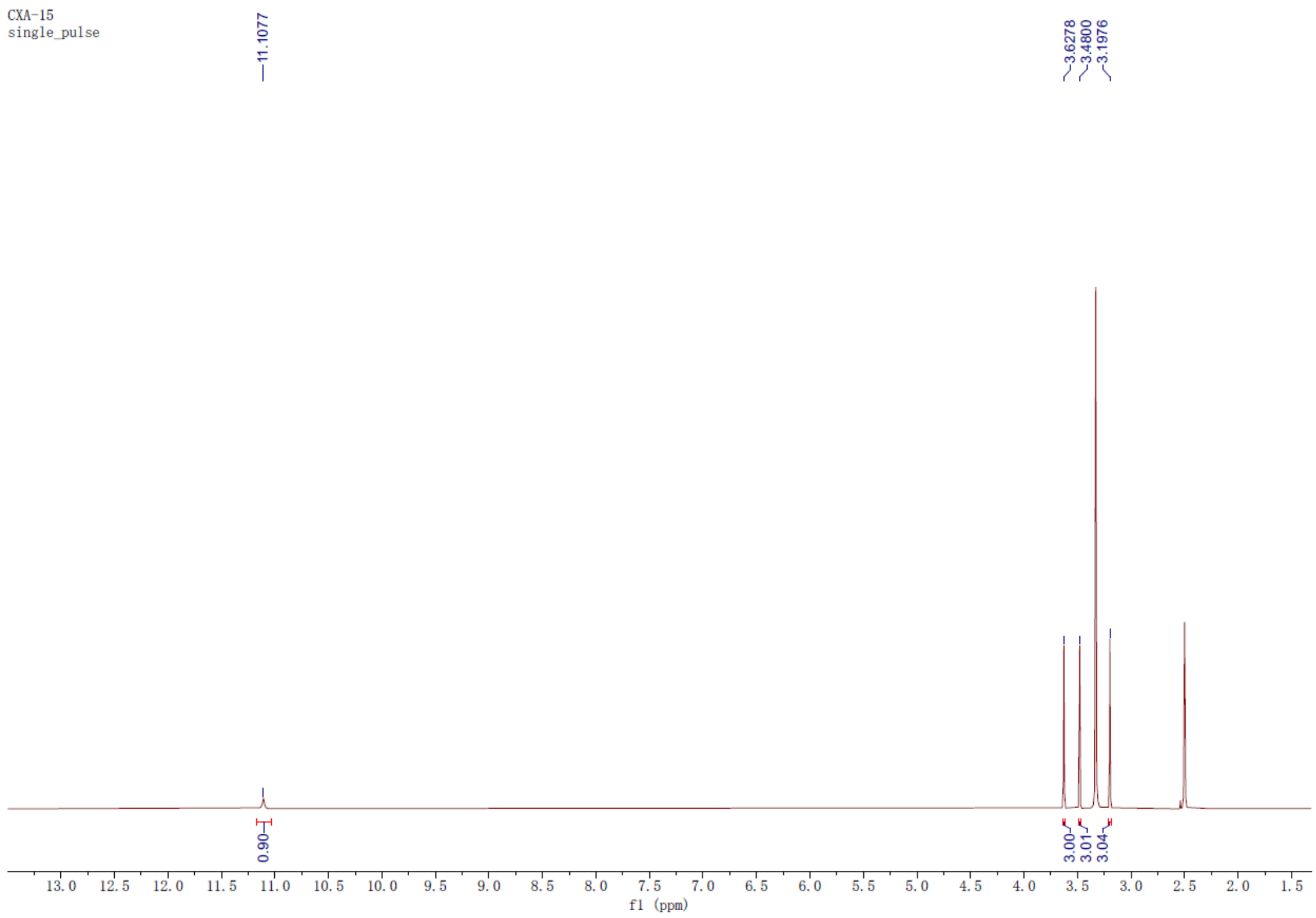


**Figure S51.** $^{1}H$ NMR (600 MHz) spectrum of compound **20** in DMSO-$d_6$.

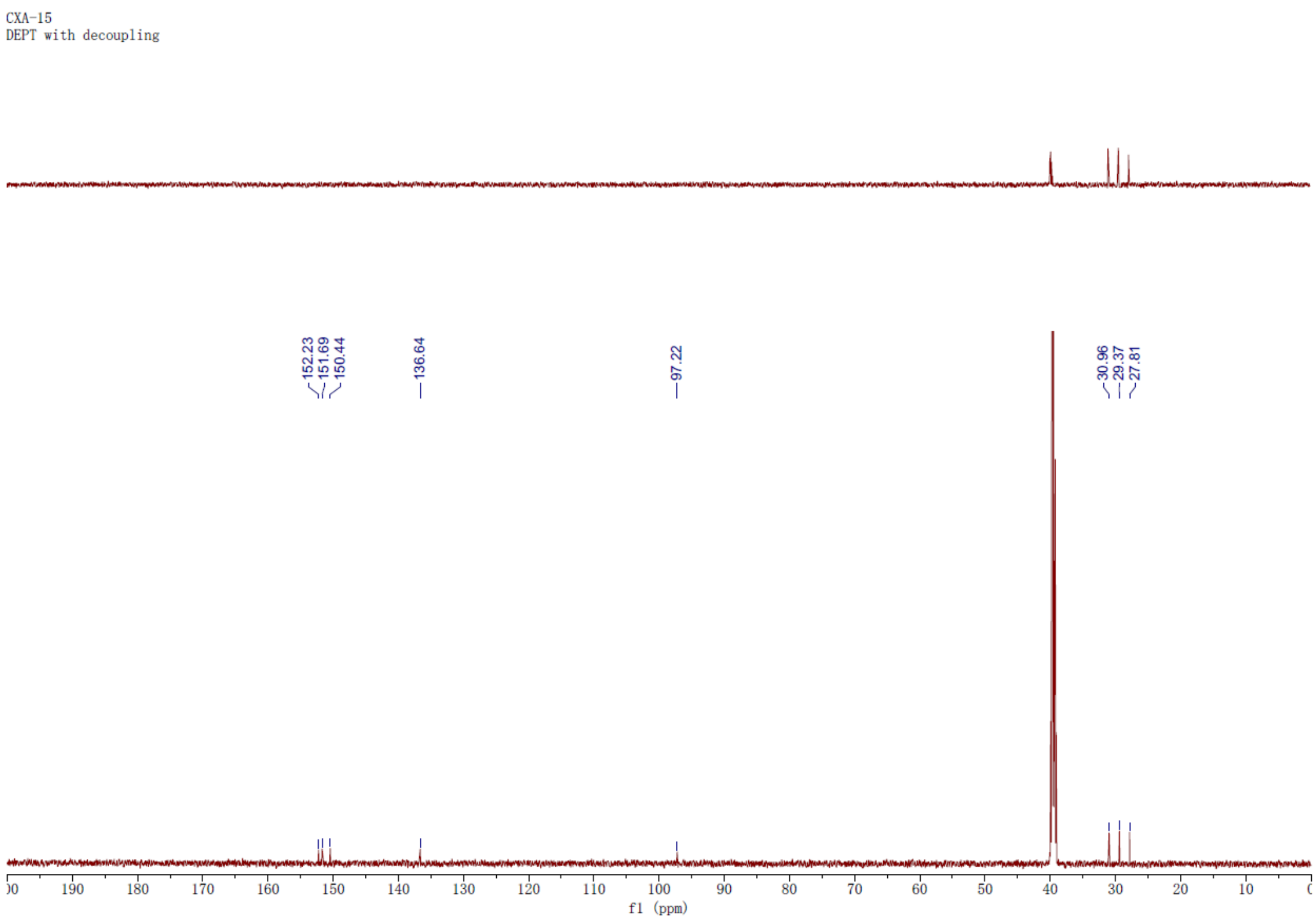


**Figure S52.** $^{13}C$ NMR (151 MHz) spectrum of compound **20** in DMSO-$d_6$.

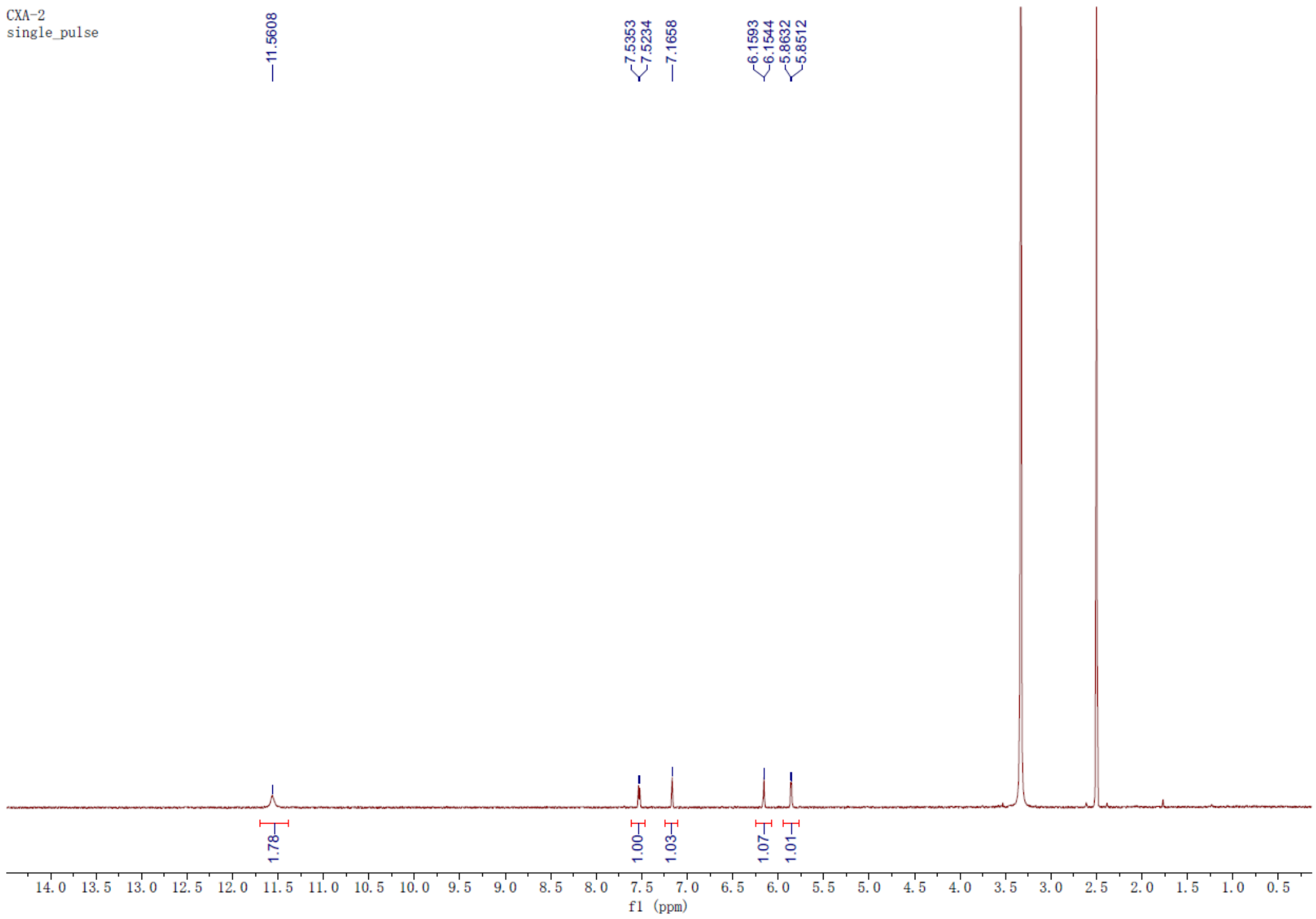


**Figure S53.** $^{1}H$ NMR (600 MHz) spectrum of compound **21** in DMSO-$d_6$.

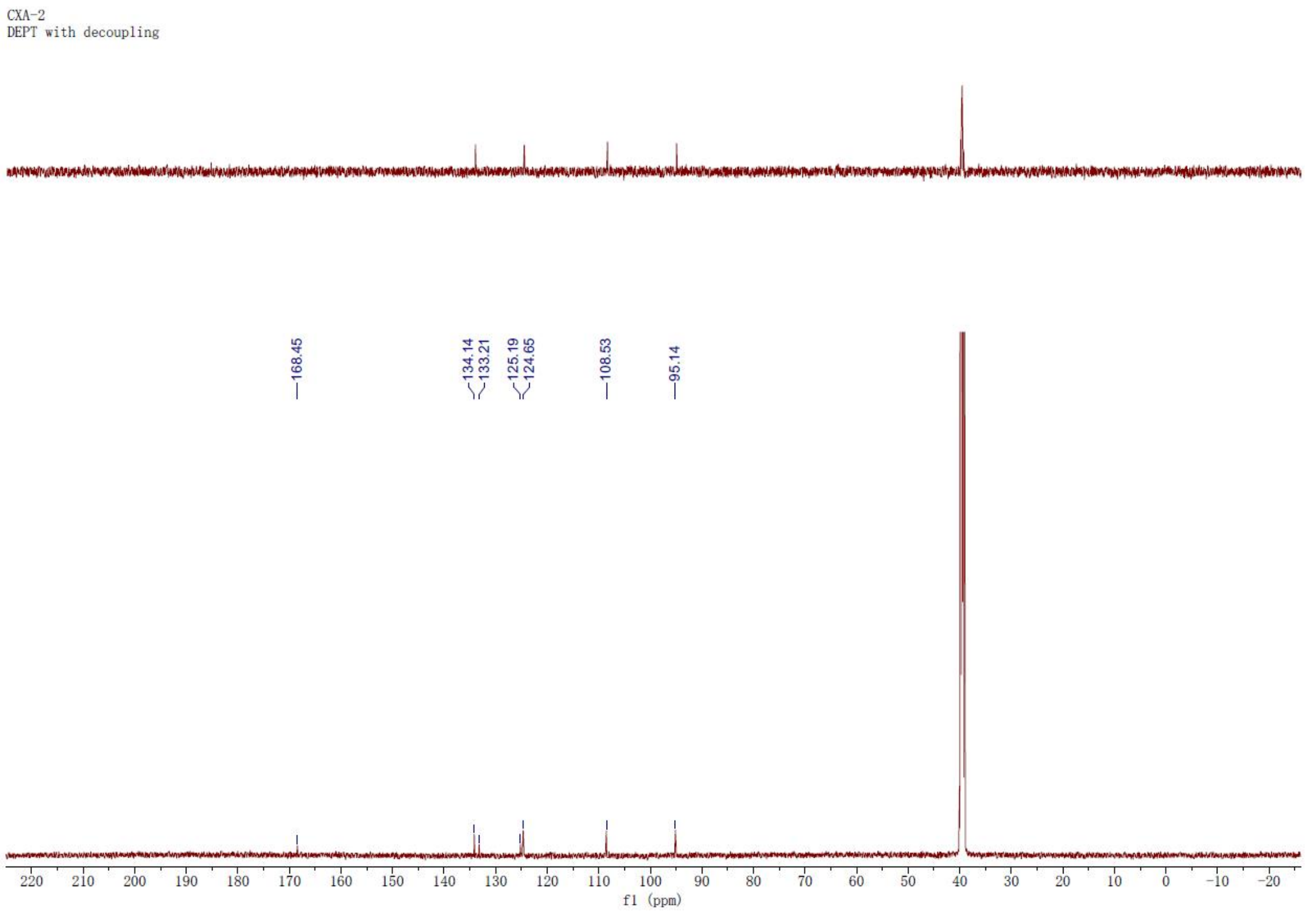


**Figure S54.** $^{13}C$ NMR (151 MHz) spectrum of compound **21** in DMSO-$d_6$.

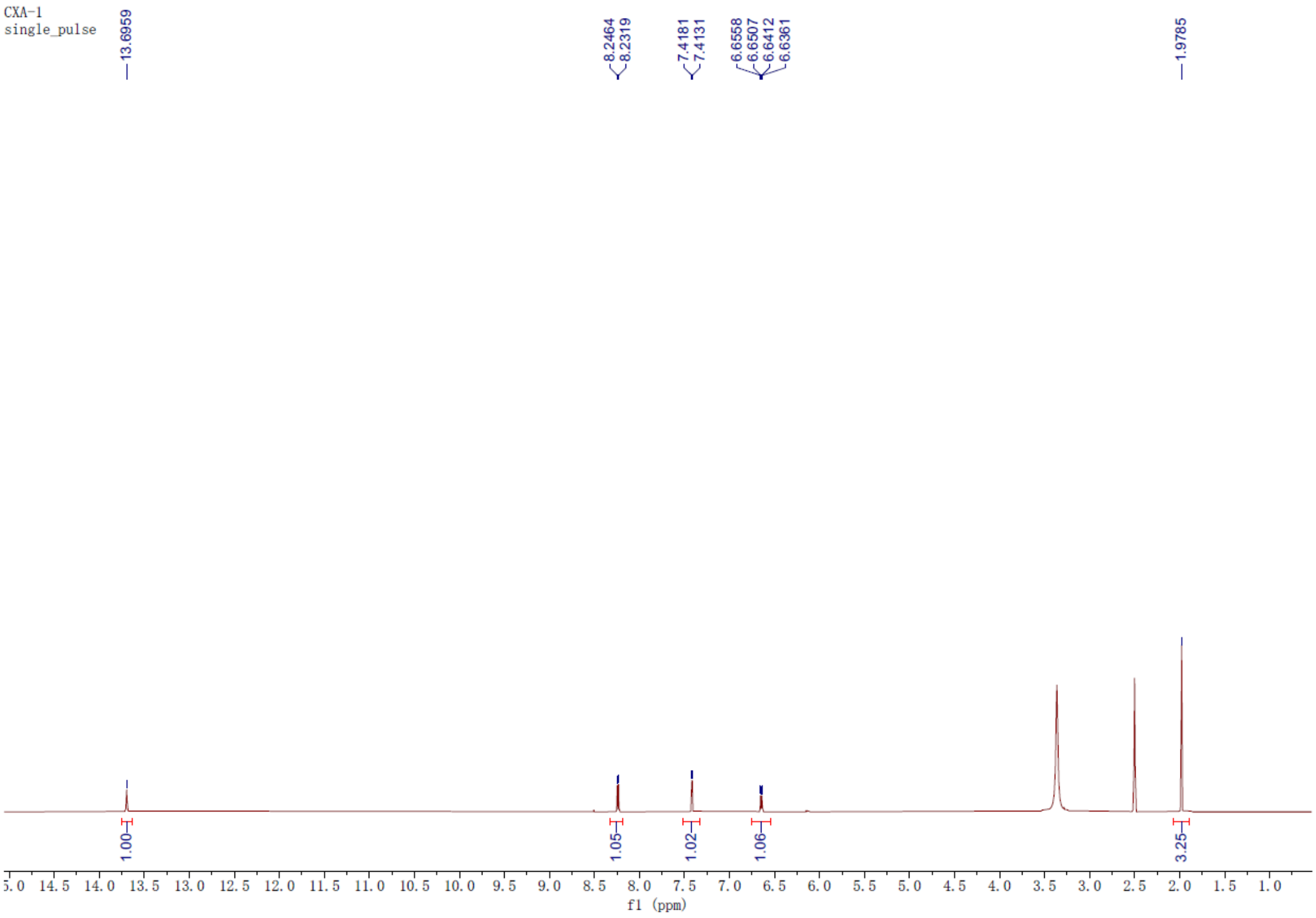


**Figure S55.** $^{1}H$ NMR (600 MHz) spectrum of compound **22** in DMSO-*d*6.

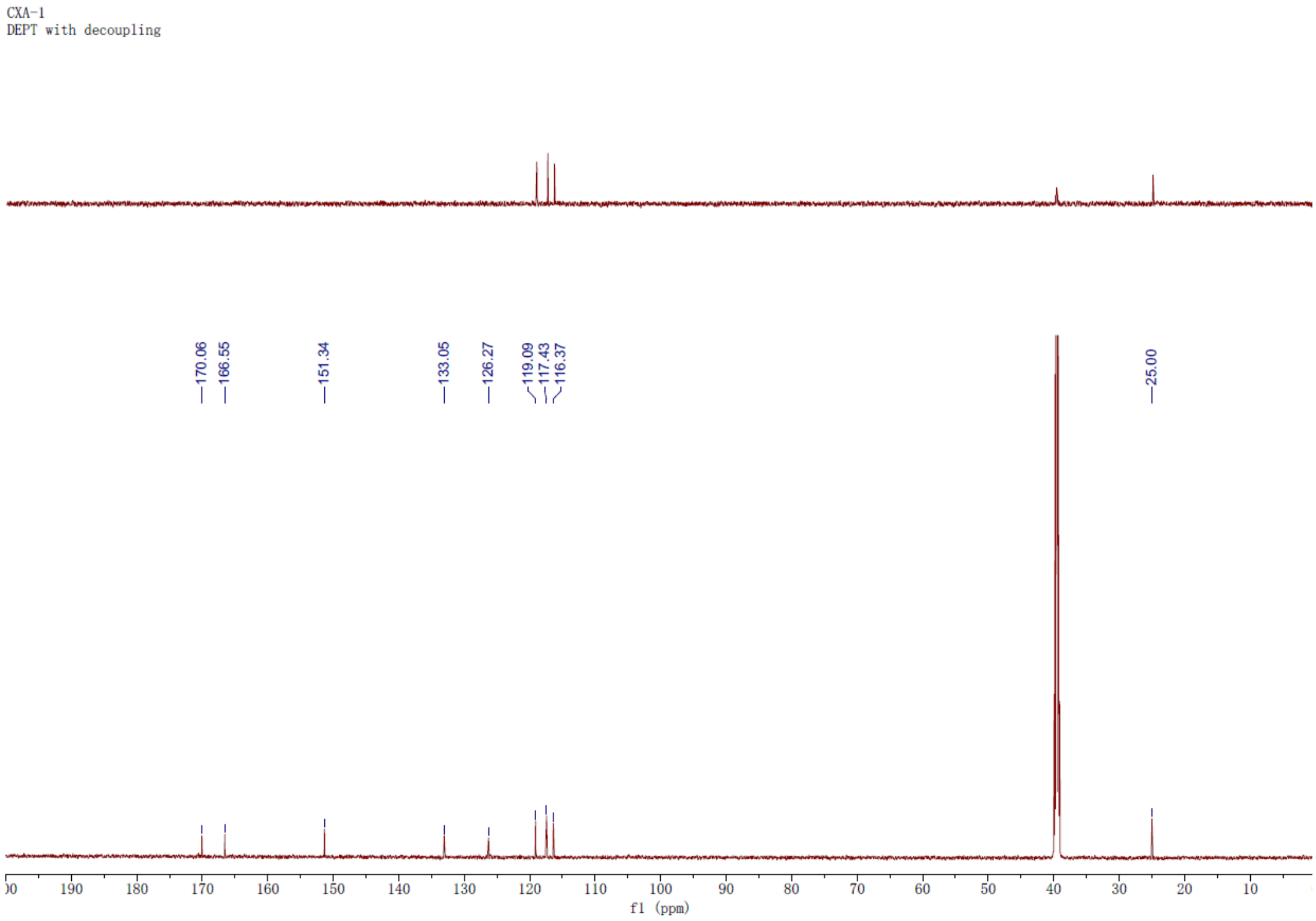


**Figure S56.** $^{13}C$ NMR (151 MHz) spectrum of compound **22** in DMSO-*d*6.

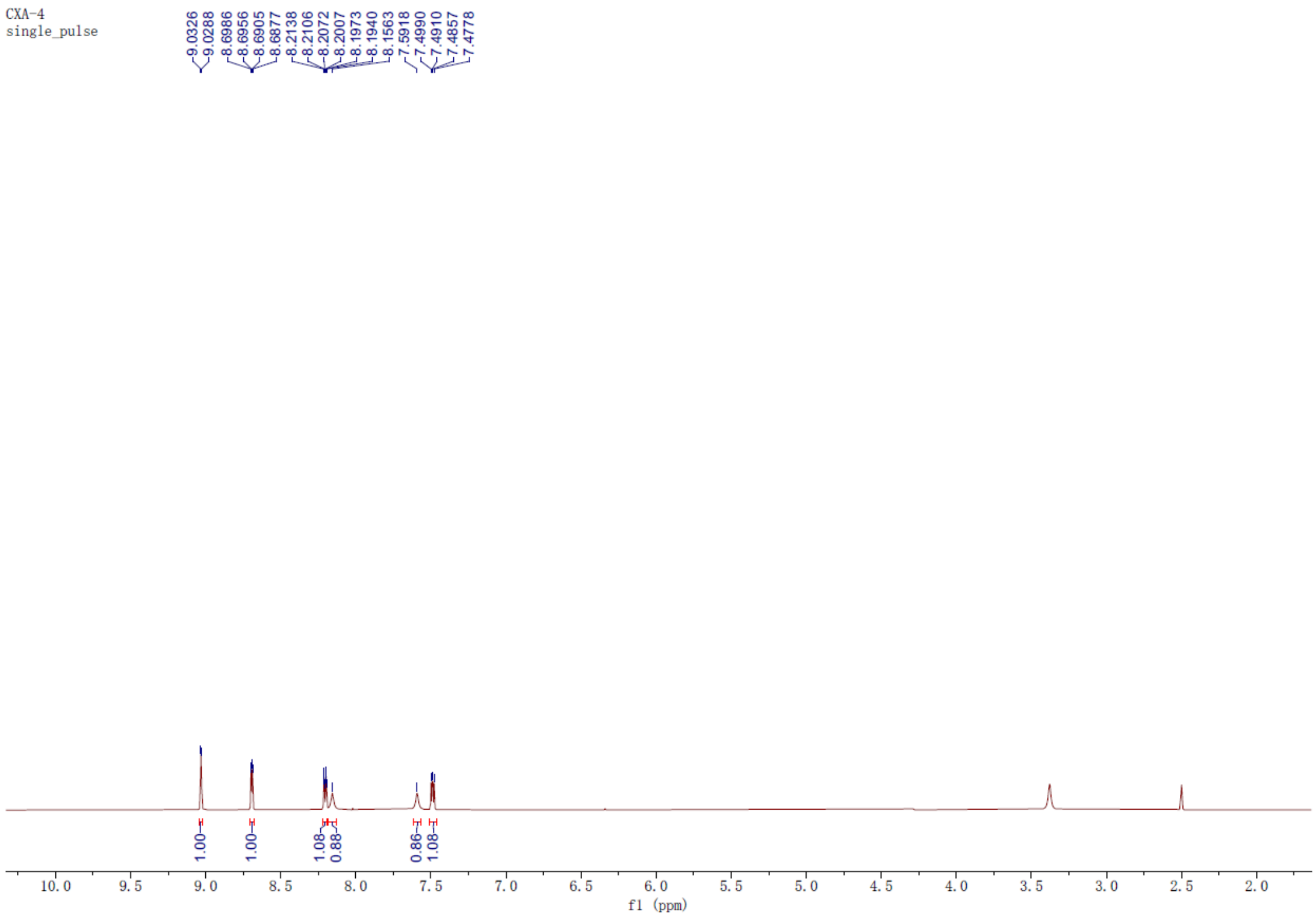


**Figure S57.** $^{1}$H NMR (600 MHz) spectrum of compound **23** in DMSO-$d_6$.

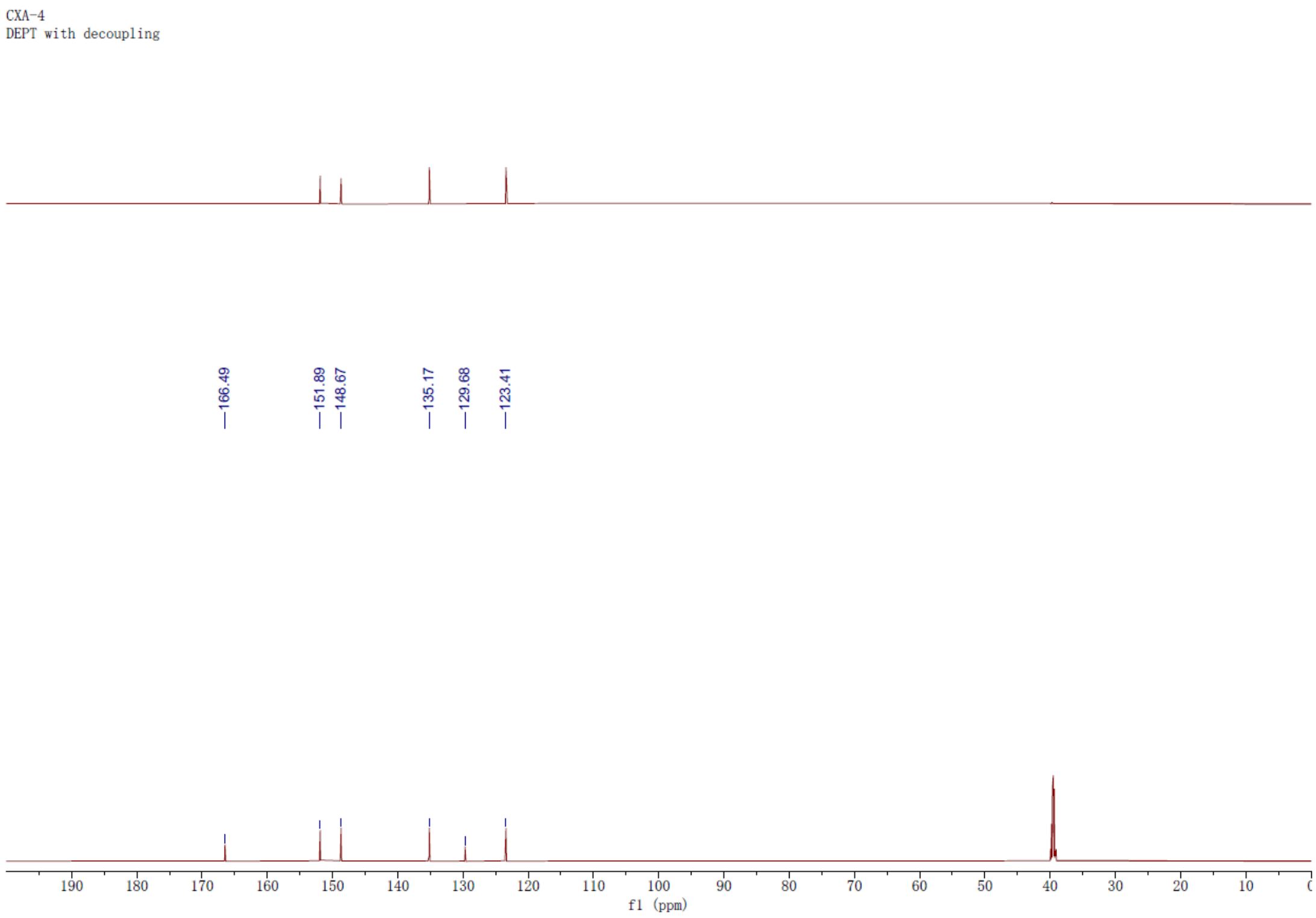


**Figure S58.** $^{13}$C NMR (151 MHz) spectrum of compound **23** in DMSO-$d_6$.

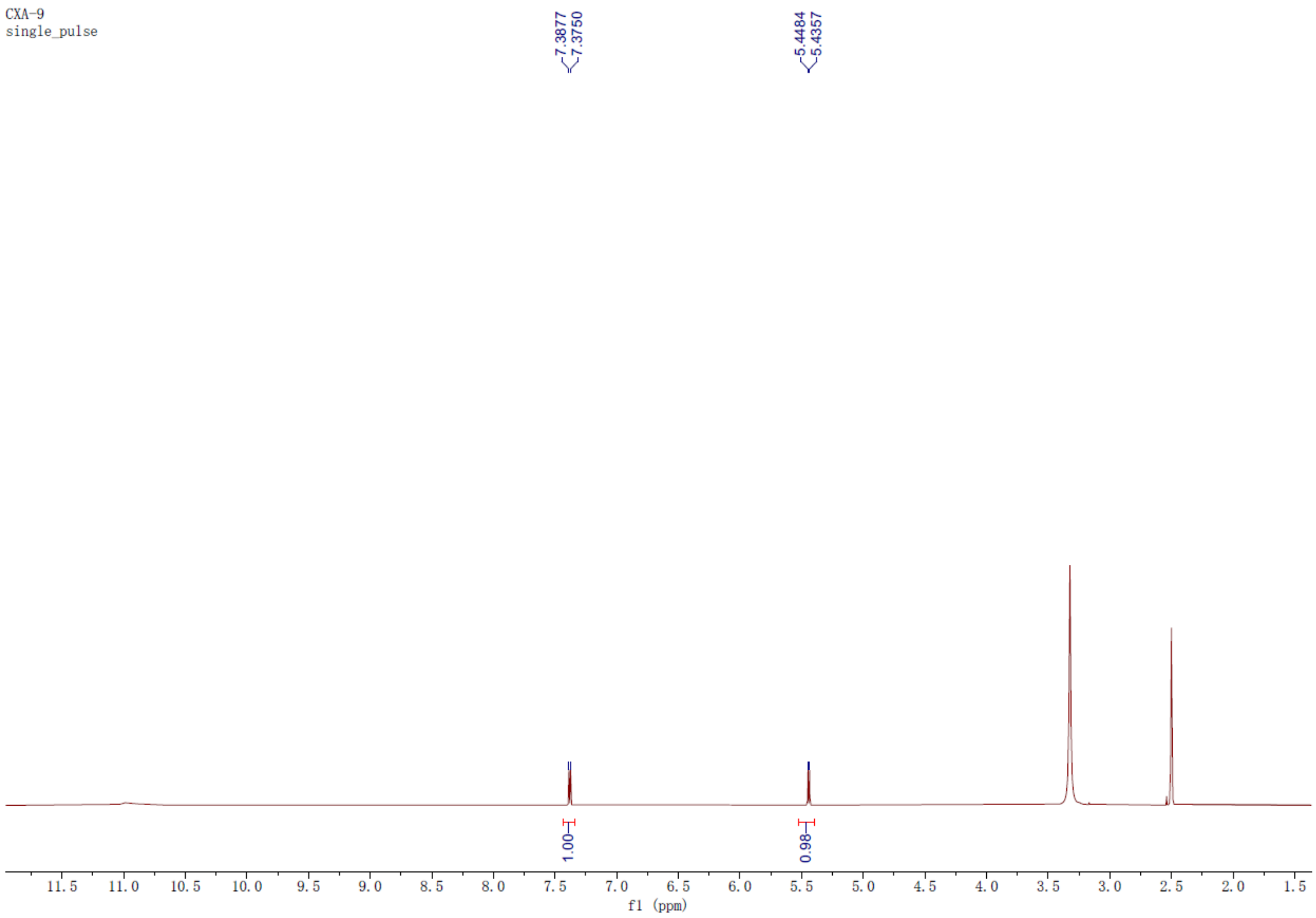


**Figure S59.** $^{1}$H NMR (600 MHz) spectrum of compound **24** in DMSO-*d*6.

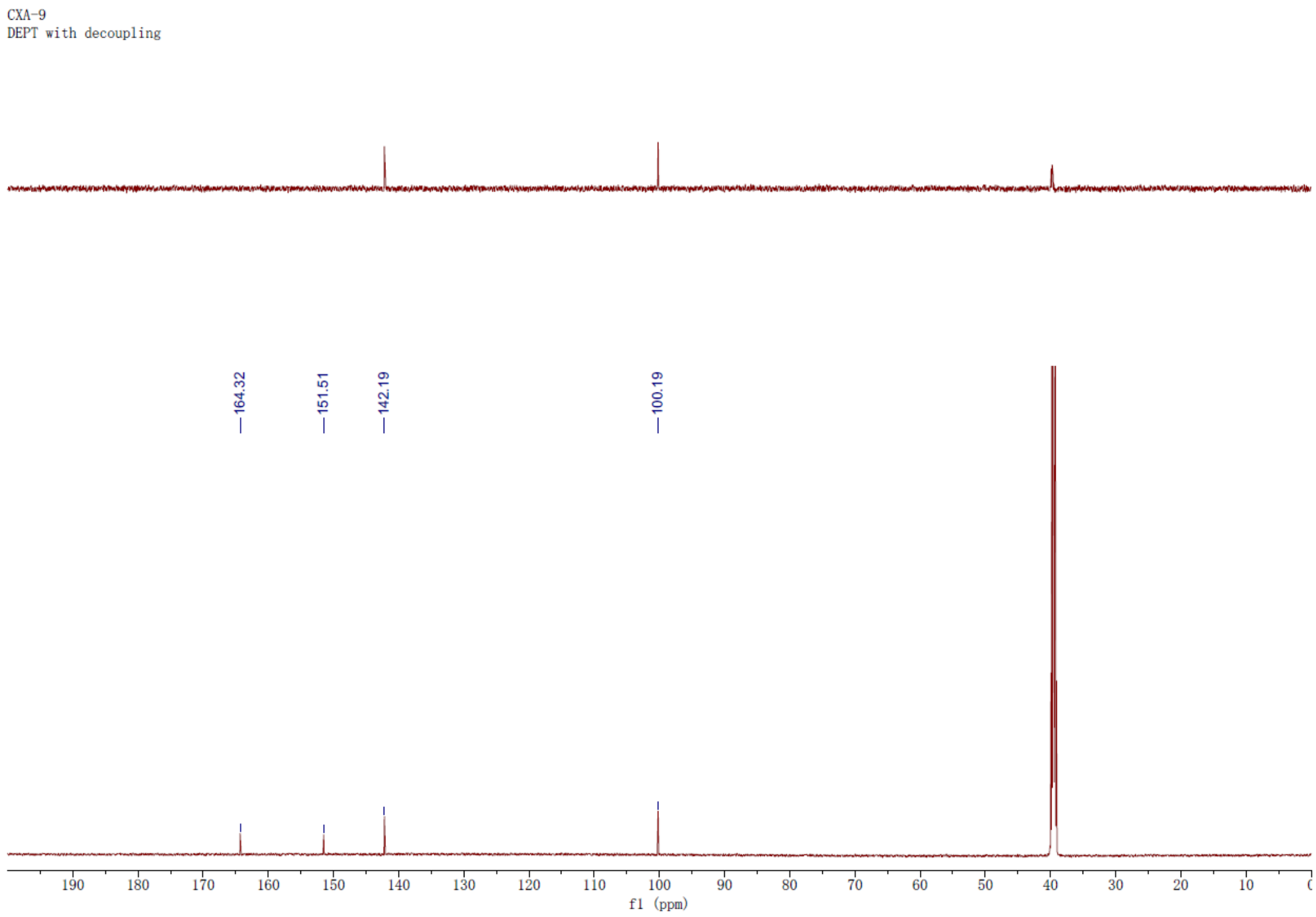


**Figure S60.** $^{13}$C NMR (151 MHz) spectrum of compound **24** in DMSO-*d*6.

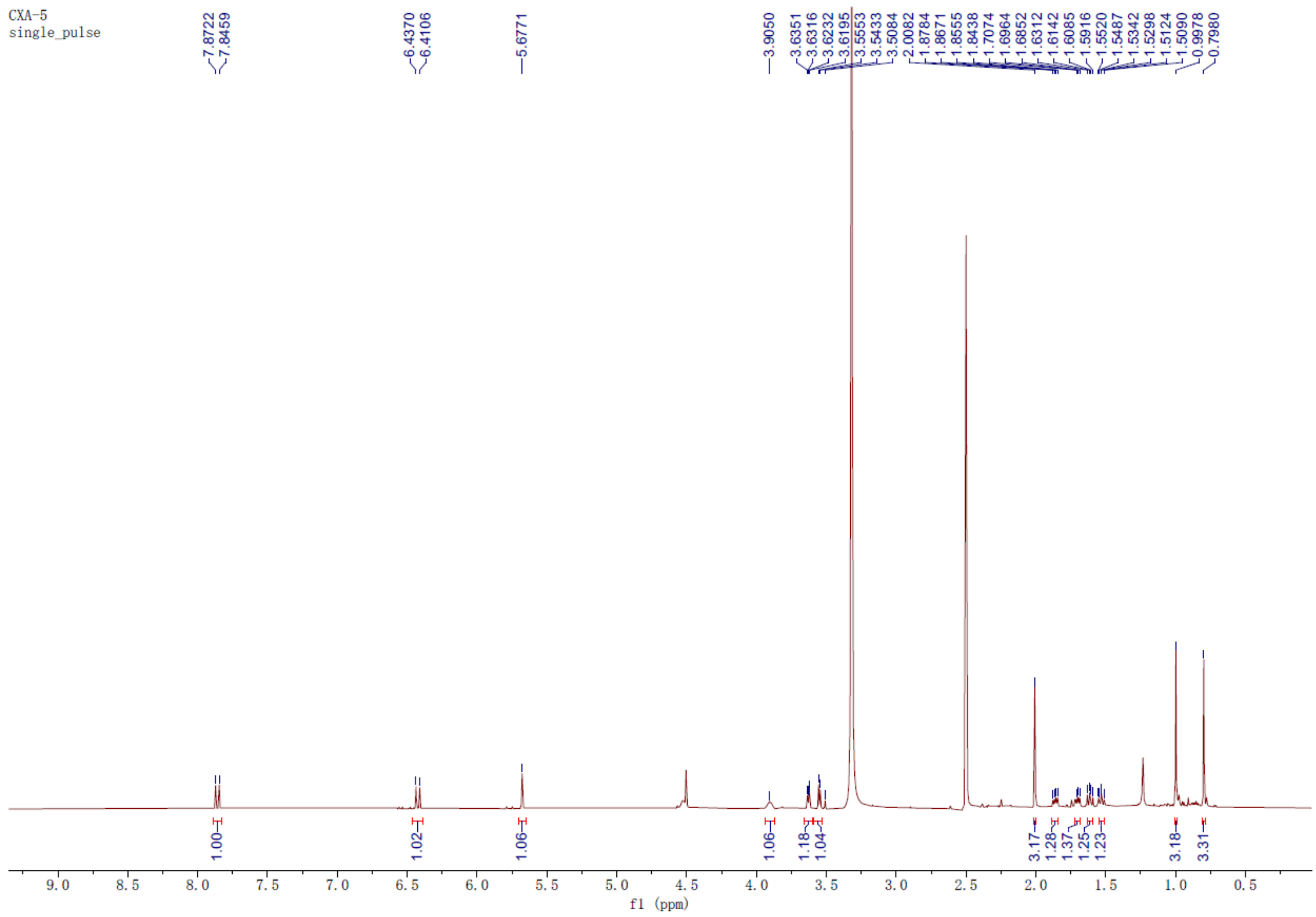


**Figure S61.** $^{1}$H NMR (600 MHz) spectrum of compound **25** in DMSO-$d_6$.

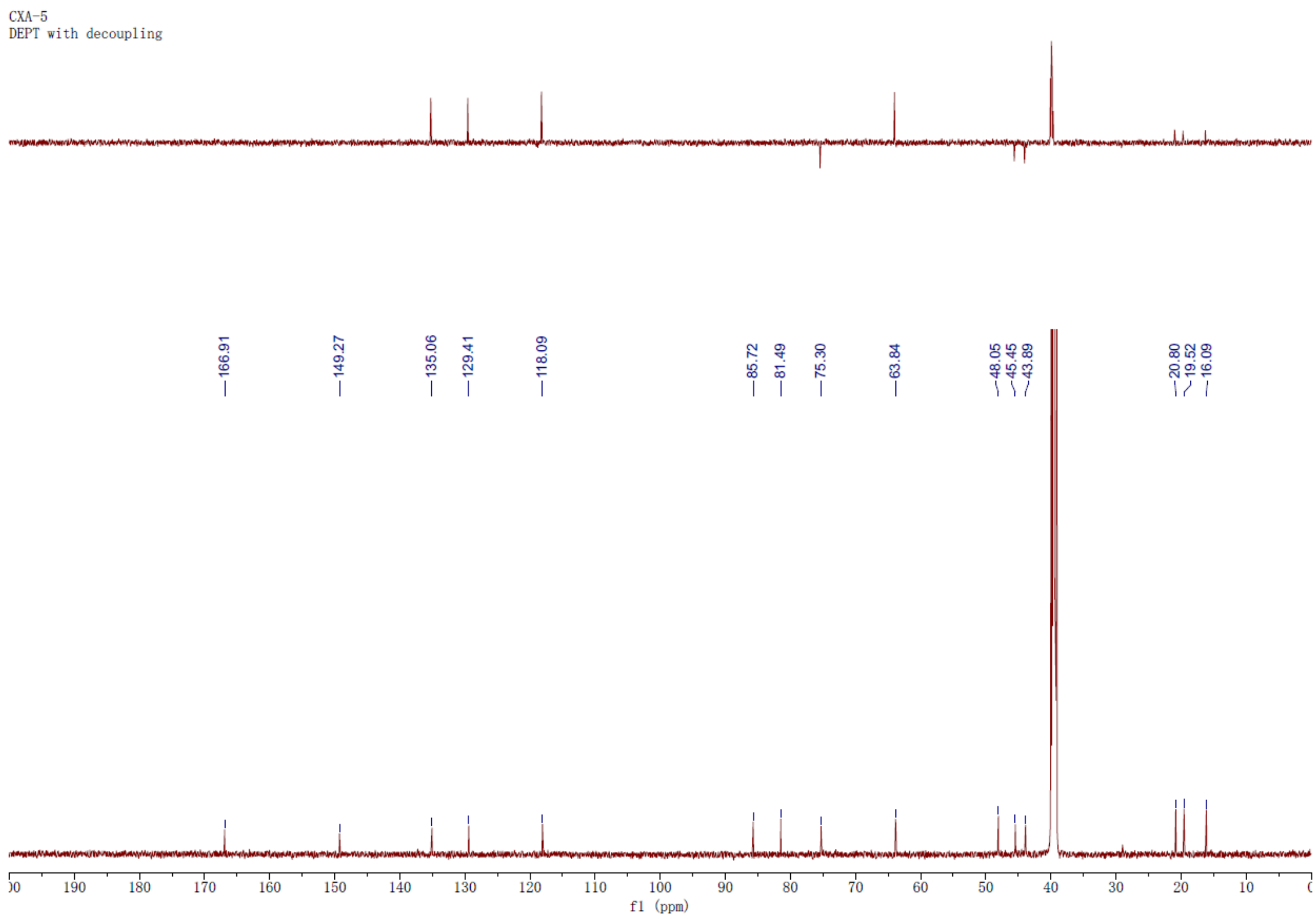


**Figure S62.** $^{13}$C NMR (151 MHz) spectrum of compound **25** in DMSO-$d_6$.

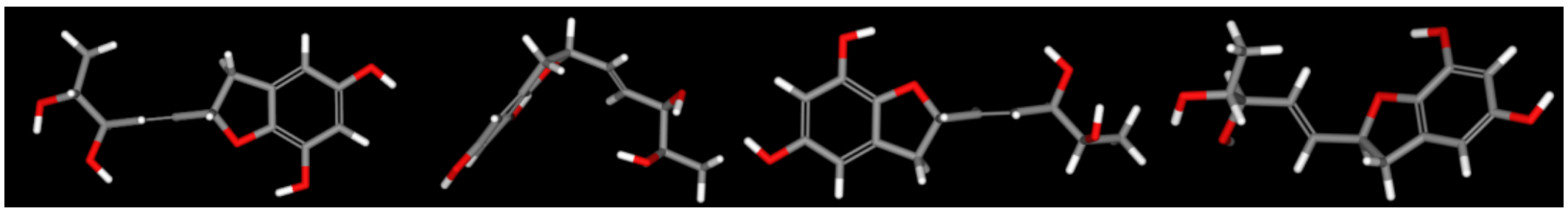

(2*S*,10*R*,11*R*)-**1C1** (2*S*,10*R*,11*R*)-**1C2** (2*S*,10*R*,11*R*)-**1C3** (2*S*,10*R*,11*R*)-**1C4**

| Conformers | Energy (Hartree) | Energy (kcal/mol) | Population (%) | Theory level |
|---|---|---|---|---|
| (2*S*,10*R*,11*R*)-**1C1** | -881.35144760 | -553056.38 | 40.8% | B3LYP/6-311G(d,p) |
| (2*S*,10*R*,11*R*)-**1C2** | -881.35102870 | -553056.12 | 26.3% | B3LYP/6-311G(d,p) |
| (2*S*,10*R*,11*R*)-**1C3** | -881.35072030 | -553055.92 | 18.8% | B3LYP/6-311G(d,p) |
| (2*S*,10*R*,11*R*)-**1C4** | -881.35044270 | -553055.75 | 14.1% | B3LYP/6-311G(d,p) |

**Figure S63.** Gibbs free energy and equilibrium populations of low-energy conformers of **1** in ECD calculations.

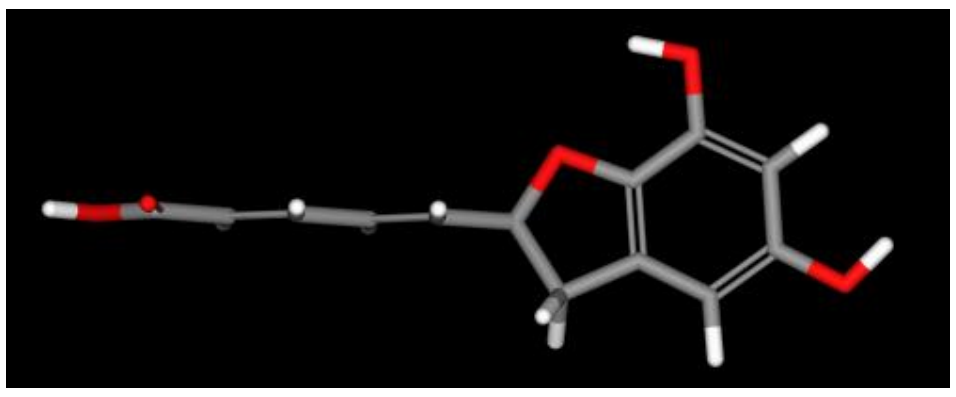

(2*S*)-**2C1**

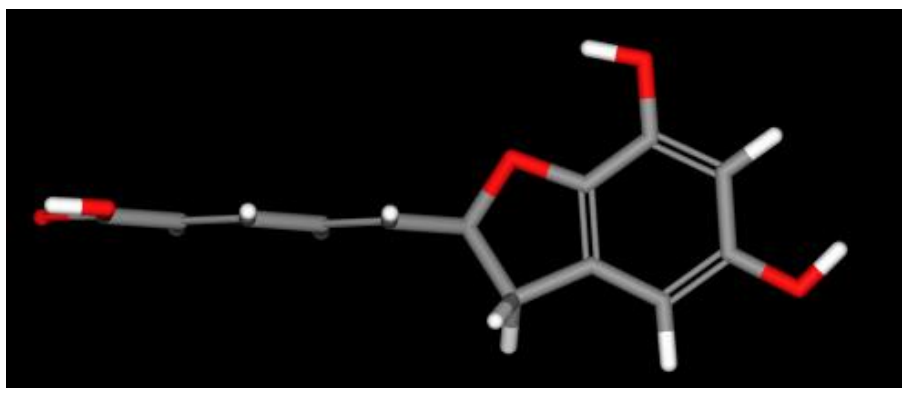

(2*S*)-**2C2**

| Conformers | Energy (Hartree) | Energy (kcal/mol) | Population (%) | Theory level |
|---|---|---|---|---|
| (2*S*)-**2C1** | -878.95310150 | -551551.39 | 72% | B3LYP/6-311G(d,p) |
| (2*S*)-**2C2** | -878.95220480 | -551550.83 | 28% | B3LYP/6-311G(d,p) |

**Figure S64.** Gibbs free energy and equilibrium populations of low-energy conformers of **2** in ECD calculations.

**Table S1.** Relative free energies and Boltzmann populations of low-energy conformers of compounds **1** and **2** within the 7 kcal/mol energy window

| Compound **1** | | | Compound **2** | | |
|---|---|---|---|---|---|
| Conformer | Energy (kcal/mol) | Population (%) | Conformer | Energy (kcal/mol) | Population (%) |
| 1 | 43.58 | 17 | 1 | -10.25 | 11.8 |
| 2 | 43.9 | 9.9 | 2 | -10.18 | 10.5 |
| 3 | 43.97 | 8.8 | 3 | -10.09 | 9 |
| 4 | 43.98 | 8.6 | 4 | -9.95 | 7.1 |
| 5 | 44.14 | 6.6 | 5 | -9.79 | 5.4 |
| 6 | 44.22 | 5.8 | 6 | -9.74 | 5 |
| 7 | 44.23 | 5.7 | 7 | -9.71 | 4.8 |
| 8 | 44.37 | 4.5 | 8 | -9.67 | 4.4 |
| 9 | 44.43 | 4 | 9 | -9.58 | 3.8 |
| 10 | 44.57 | 3.2 | 10 | -9.54 | 3.6 |
| 11 | 44.57 | 3.2 | 11 | -9.48 | 3.2 |
| 12 | 44.82 | 2.1 | 12 | -9.33 | 2.5 |
| 13 | 44.9 | 1.8 | 13 | -9.32 | 2.5 |
| 14 | 44.95 | 1.7 | 14 | -9.27 | 2.3 |
| 15 | 44.98 | 1.6 | 15 | -9.16 | 1.9 |
| 16 | 45.07 | 1.4 | 16 | -9.15 | 1.8 |
| | | | 17 | -9.12 | 1.8 |
| | | | 18 | -9.03 | 1.5 |
| | | | 19 | -9 | 1.4 |
| | | | 20 | -9 | 1.4 |
| | | | 21 | -9 | 1.4 |
| | | | 22 | -8.9 | 1.2 |
| | | | 23 | -8.89 | 1.2 |
| | | | 24 | -8.84 | 1.1 |

**Table S2.** Experimental and calculated chemical shifts of **1** for DP4+ probability statistical analysis.

| Functional | Solvent | Basis Set | | Type of Data | | Type of Data | | Type of Data | |
|---|---|---|---|---|---|---|---|---|---|
| mPW1PW91 | DMSO | 6-311G(d,p) | | Scaled Shifts | | Scaled Shifts | | Scaled Shifts | |
| DP4+ | | **1A** | | **1B** | | **1C** | | **1D** | |
| Bayes's theorem probability | | 98.54% | | 1.41% | | 0.00% | | 0.05% | |
| Nuclei | $\delta_{experimental}$ | $\delta_{calculated}$ | $\delta_{corrected}$ | $\delta_{calculated}$ | $\delta_{corrected}$ | $\delta_{calculated}$ | $\delta_{corrected}$ | $\delta_{calculated}$ | $\delta_{corrected}$ |
| C-2 | 82.4 | 87.0 | 83.4 | 88.3 | 83.5 | 89.2 | 84.9 | 87.3 | 83.5 |
| C-3 | 36.7 | 41.4 | 39.8 | 41.7 | 38.3 | 42.4 | 39.7 | 41.4 | 39.1 |
| C-3a | 127.8 | 134.0 | 128.4 | 134.7 | 128.6 | 135.4 | 129.6 | 134.0 | 128.7 |
| C-4 | 102.1 | 105.5 | 101.1 | 105.2 | 100.0 | 105.2 | 100.5 | 105.5 | 101.1 |
| C-5 | 151.6 | 158.6 | 152.1 | 158.4 | 151.7 | 158.5 | 152.0 | 158.6 | 152.5 |
| C-6 | 102.5 | 102.4 | 98.2 | 102.4 | 97.2 | 102.4 | 97.7 | 102.4 | 98.1 |
| C-7 | 141.1 | 147.5 | 141.4 | 147.2 | 140.8 | 147.3 | 141.1 | 147.6 | 141.9 |
| C-7a | 139.3 | 144.8 | 138.8 | 145.0 | 138.6 | 144.9 | 138.9 | 144.7 | 139.1 |
| C-8 | 129.5 | 139.2 | 133.5 | 139.1 | 132.9 | 137.7 | 131.9 | 140.0 | 134.5 |
| C-9 | 133.7 | 137.4 | 131.7 | 139.1 | 132.9 | 137.6 | 131.7 | 133.7 | 128.4 |
| C-10 | 74.8 | 76.7 | 73.6 | 80.8 | 76.2 | 77.8 | 73.9 | 77.0 | 73.5 |
| C-11 | 69.7 | 72.9 | 69.9 | 76.6 | 72.2 | 74.9 | 71.1 | 75.3 | 71.8 |
| C-12 | 19.1 | 19.1 | 18.4 | 20.4 | 17.5 | 19.3 | 17.2 | 20.0 | 18.3 |

**Table S3.** Cartesian coordinates for the low-energy reoptimized MMFF conformers of **1** at B3LYP/6-31G(d,p) level of theory in methanol.

(2*S*,10*R*,11*R*)-**1C1**

| Center Number | Atomic Number | Atomic Element | Coordinates (Angstroms) X | Y | Z |
|---|---|---|---|---|---|
| 1 | 6 | C | -3.351794 | 1.568392 | -0.076861 |
| 2 | 6 | C | -4.539074 | 0.83687 | 0.07498 |
| 3 | 6 | C | -4.526719 | -0.56032 | 0.177458 |
| 4 | 6 | C | -3.317208 | -1.261036 | 0.127068 |
| 5 | 6 | C | -2.146894 | -0.522927 | -0.019793 |
| 6 | 6 | C | -2.156333 | 0.860135 | -0.12629 |
| 7 | 6 | C | -0.713366 | 1.301893 | -0.232203 |
| 8 | 6 | C | 0.018102 | -0.040928 | -0.531565 |
| 9 | 8 | O | -0.891014 | -1.093746 | -0.07607 |
| 10 | 8 | O | -5.70864 | 1.553171 | 0.115361 |
| 11 | 8 | O | -3.309916 | -2.623326 | 0.21983 |
| 12 | 6 | C | 1.33627 | -0.207623 | 0.154198 |
| 13 | 6 | C | 2.502233 | -0.355223 | -0.480989 |
| 14 | 6 | C | 3.832807 | -0.495331 | 0.199853 |
| 15 | 6 | C | 4.851653 | 0.598189 | -0.175357 |

| | | | | | |
|---|---|---|---|---|---|
| 16 | 6 | C | 4.448555 | 1.984369 | 0.306305 |
| 17 | 8 | O | 4.467426 | -1.723721 | -0.204425 |
| 18 | 8 | O | 6.105474 | 0.284762 | 0.412489 |
| 19 | 1 | H | -3.395827 | 2.649523 | -0.153938 |
| 20 | 1 | H | -5.450214 | -1.123227 | 0.287779 |
| 21 | 1 | H | -0.526145 | 2.036031 | -1.022089 |
| 22 | 1 | H | -0.363001 | 1.740432 | 0.712584 |
| 23 | 1 | H | 0.133482 | -0.164036 | -1.616726 |
| 24 | 1 | H | -6.44956 | 0.937333 | 0.222125 |
| 25 | 1 | H | -2.386994 | -2.915667 | 0.133339 |
| 26 | 1 | H | 1.303236 | -0.179902 | 1.244247 |
| 27 | 1 | H | 2.527326 | -0.398518 | -1.571013 |
| 28 | 1 | H | 3.705912 | -0.478881 | 1.293422 |
| 29 | 1 | H | 4.937841 | 0.600043 | -1.276517 |
| 30 | 1 | H | 5.236913 | 2.700836 | 0.056905 |
| 31 | 1 | H | 4.318401 | 1.98959 | 1.394343 |
| 32 | 1 | H | 3.515698 | 2.312782 | -0.162431 |
| 33 | 1 | H | 3.914126 | -2.459337 | 0.100039 |
| 34 | 1 | H | 6.242769 | -0.662873 | 0.239639 |

| (2*S*,10*R*,11*R*)-**1C2** | | | | | |
|---|---|---|---|---|---|
| Center Number | Atomic Number | Atomic Element | Coordinates (Angstroms) X | Y | Z |
| 1 | 6 | C | 2.635903 | -0.22817 | -1.470577 |
| 2 | 6 | C | 3.29962 | -1.135707 | -0.631591 |
| 3 | 6 | C | 3.13383 | -1.098384 | 0.759322 |
| 4 | 6 | C | 2.290184 | -0.148166 | 1.343744 |
| 5 | 6 | C | 1.6393 | 0.74574 | 0.499559 |
| 6 | 6 | C | 1.801529 | 0.713215 | -0.877336 |
| 7 | 6 | C | 0.97271 | 1.843021 | -1.446958 |
| 8 | 6 | C | 0.085068 | 2.237226 | -0.222049 |
| 9 | 8 | O | 0.78066 | 1.728457 | 0.95129 |
| 10 | 8 | O | 4.114817 | -2.058068 | -1.236918 |
| 11 | 8 | O | 2.121516 | -0.122187 | 2.698977 |
| 12 | 6 | C | -1.306288 | 1.669347 | -0.318002 |
| 13 | 6 | C | -1.866489 | 0.829343 | 0.553161 |
| 14 | 6 | C | -3.248399 | 0.23319 | 0.394421 |
| 15 | 6 | C | -3.152895 | -1.314188 | 0.325442 |
| 16 | 6 | C | -4.511493 | -1.99681 | 0.37954 |
| 17 | 8 | O | -3.924454 | 0.709308 | -0.758339 |
| 18 | 8 | O | -2.556441 | -1.663259 | -0.932343 |

| | | | | | |
|---|---|---|---|---|---|
| 19 | 1 | H | 2.787652 | -0.282925 | -2.543321 |
| 20 | 1 | H | 3.647929 | -1.805658 | 1.405239 |
| 21 | 1 | H | 0.362923 | 1.550498 | -2.308082 |
| 22 | 1 | H | 1.609054 | 2.680029 | -1.762292 |
| 23 | 1 | H | 0.03649 | 3.324806 | -0.100146 |
| 24 | 1 | H | 4.512102 | -2.621217 | -0.555351 |
| 25 | 1 | H | 1.527523 | 0.618481 | 2.9063 |
| 26 | 1 | H | -1.886753 | 2.000521 | -1.178566 |
| 27 | 1 | H | -1.307043 | 0.518237 | 1.434791 |
| 28 | 1 | H | -3.862217 | 0.513058 | 1.262301 |
| 29 | 1 | H | -2.524337 | -1.674907 | 1.154148 |
| 30 | 1 | H | -4.983846 | -1.830775 | 1.353897 |
| 31 | 1 | H | -5.170318 | -1.595882 | -0.396125 |
| 32 | 1 | H | -4.397656 | -3.073676 | 0.224157 |
| 33 | 1 | H | -3.62041 | 0.12328 | -1.477401 |
| 34 | 1 | H | -1.65535 | -1.295823 | -0.932933 |

(2*S*,10*R*,11*R*)-**1C3**

| Center Number | Atomic Number | Atomic Element | Coordinates (Angstroms) X | Y | Z |
|---|---|---|---|---|---|
| 1 | 6 | C | 3.453742 | -1.570629 | 0.03238 |
| 2 | 6 | C | 4.622118 | -0.795658 | 0.076902 |
| 3 | 6 | C | 4.569315 | 0.604315 | 0.065987 |
| 4 | 6 | C | 3.337115 | 1.263635 | 0.007285 |
| 5 | 6 | C | 2.185936 | 0.483017 | -0.032886 |
| 6 | 6 | C | 2.23533 | -0.903292 | -0.026747 |
| 7 | 6 | C | 0.804285 | -1.39365 | -0.038035 |
| 8 | 6 | C | 0.018478 | -0.10163 | -0.412839 |
| 9 | 8 | O | 0.911665 | 1.011296 | -0.084671 |
| 10 | 8 | O | 5.814663 | -1.472435 | 0.128879 |
| 11 | 8 | O | 3.290066 | 2.628275 | -0.009434 |
| 12 | 6 | C | -1.274926 | 0.083118 | 0.314841 |
| 13 | 6 | C | -2.470814 | 0.130692 | -0.278161 |
| 14 | 6 | C | -3.771878 | 0.286145 | 0.457395 |
| 15 | 6 | C | -4.812323 | -0.774707 | 0.039349 |
| 16 | 6 | C | -6.070574 | -0.727264 | 0.910224 |
| 17 | 8 | O | -4.384186 | 1.548267 | 0.129515 |
| 18 | 8 | O | -5.124876 | -0.610677 | -1.339861 |
| 19 | 1 | H | 3.529052 | -2.652761 | 0.041268 |
| 20 | 1 | H | 5.477931 | 1.20064 | 0.093617 |
| 21 | 1 | H | 0.61025 | -2.194009 | -0.758861 |

| | | | | | |
|---|---|---|---|---|---|
| 22 | 1 | H | 0.505306 | -1.765124 | 0.952128 |
| 23 | 1 | H | -0.146441 | -0.071826 | -1.497912 |
| 24 | 1 | H | 6.538938 | -0.828761 | 0.1544 |
| 25 | 1 | H | 2.355694 | 2.885852 | -0.082072 |
| 26 | 1 | H | -1.193289 | 0.152851 | 1.400805 |
| 27 | 1 | H | -2.556089 | 0.060498 | -1.36207 |
| 28 | 1 | H | -3.598464 | 0.221505 | 1.542905 |
| 29 | 1 | H | -4.344879 | -1.762757 | 0.122861 |
| 30 | 1 | H | -5.838004 | -0.957532 | 1.957156 |
| 31 | 1 | H | -6.796556 | -1.460464 | 0.54613 |
| 32 | 1 | H | -6.527959 | 0.266904 | 0.872733 |
| 33 | 1 | H | -3.693927 | 2.228061 | 0.167948 |
| 34 | 1 | H | -5.335037 | 0.334559 | -1.440633 |

(2*S*,10*R*,11*R*)-**1C4**

| Center Number | Atomic Number | Atomic Element | Coordinates (Angstroms) X | Y | Z |
|---|---|---|---|---|---|
| 1 | 6 | C | -3.238767 | -1.207356 | -0.877629 |
| 2 | 6 | C | -4.218475 | -0.206001 | -0.804069 |
| 3 | 6 | C | -4.006832 | 0.96877 | -0.070828 |
| 4 | 6 | C | -2.801687 | 1.167727 | 0.610837 |
| 5 | 6 | C | -1.837118 | 0.169045 | 0.524268 |
| 6 | 6 | C | -2.04558 | -0.998715 | -0.195074 |
| 7 | 6 | C | -0.777663 | -1.816699 | -0.088164 |
| 8 | 6 | C | -0.019501 | -1.079899 | 1.06202 |
| 9 | 8 | O | -0.604803 | 0.248466 | 1.142308 |
| 10 | 8 | O | -5.391029 | -0.434268 | -1.478834 |
| 11 | 8 | O | -2.603208 | 2.311125 | 1.330717 |
| 12 | 6 | C | 1.465609 | -1.004831 | 0.881626 |
| 13 | 6 | C | 2.164561 | 0.110819 | 0.664068 |
| 14 | 6 | C | 3.656639 | 0.147523 | 0.457205 |
| 15 | 6 | C | 4.073506 | 0.324009 | -1.019416 |
| 16 | 6 | C | 3.585853 | 1.626897 | -1.637419 |
| 17 | 8 | O | 4.303212 | -1.065759 | 0.86542 |
| 18 | 8 | O | 5.49087 | 0.313426 | -1.107633 |
| 19 | 1 | H | -3.434948 | -2.105971 | -1.452718 |
| 20 | 1 | H | -4.77232 | 1.738255 | -0.009778 |
| 21 | 1 | H | -0.201761 | -1.781376 | -1.02215 |
| 22 | 1 | H | -0.949012 | -2.86968 | 0.157372 |
| 23 | 1 | H | -0.252553 | -1.58468 | 2.012741 |
| 24 | 1 | H | -5.980202 | 0.3244 | -1.348779 |

| | | | | | |
|---|---|---|---|---|---|
| 25 | 1 | H | -1.732065 | 2.240867 | 1.755738 |
| 26 | 1 | H | 1.98334 | -1.962126 | 0.92565 |
| 27 | 1 | H | 1.645695 | 1.065399 | 0.623334 |
| 28 | 1 | H | 4.082455 | 0.99927 | 1.01307 |
| 29 | 1 | H | 3.647001 | -0.530696 | -1.571964 |
| 30 | 1 | H | 2.493553 | 1.656066 | -1.699754 |
| 31 | 1 | H | 3.933252 | 2.486798 | -1.053172 |
| 32 | 1 | H | 3.99277 | 1.721313 | -2.648636 |
| 33 | 1 | H | 4.203827 | -1.146179 | 1.82665 |
| 34 | 1 | H | 5.77737 | -0.443312 | -0.56797 |

**Table S4.** Cartesian coordinates for the low-energy reoptimized MMFF conformers of **2** at B3LYP/6-31G(d,p) level of theory in methanol.

| (2*S*)-**2C1** | | | | | |
|---|---|---|---|---|---|
| Center Number | Atomic Number | Atomic Element | Coordinates (Angstroms) X | Y | Z |
| 1 | 6 | C | -3.635924 | -1.503095 | -0.069145 |
| 2 | 6 | C | -4.759741 | -0.669967 | -0.173419 |
| 3 | 6 | C | -4.63727 | 0.725533 | -0.154039 |
| 4 | 6 | C | -3.378764 | 1.322633 | -0.026789 |
| 5 | 6 | C | -2.272741 | 0.484223 | 0.072555 |
| 6 | 6 | C | -2.390854 | -0.897459 | 0.057984 |
| 7 | 6 | C | -0.989187 | -1.46044 | 0.142925 |
| 8 | 6 | C | -0.160696 | -0.206992 | 0.562437 |
| 9 | 8 | O | -0.977266 | 0.947787 | 0.195171 |
| 10 | 8 | O | -5.979749 | -1.285774 | -0.292216 |
| 11 | 6 | C | 1.170957 | -0.089201 | -0.102284 |
| 12 | 6 | C | 2.346965 | -0.121273 | 0.548929 |
| 13 | 6 | C | 3.630763 | -0.052096 | -0.118227 |
| 14 | 6 | C | 4.820464 | -0.088836 | 0.512943 |
| 15 | 6 | C | 6.077644 | -0.010089 | -0.252817 |
| 16 | 8 | O | 7.162575 | -0.060547 | 0.565579 |
| 17 | 8 | O | 6.177771 | 0.0876 | -1.461162 |
| 18 | 8 | O | -3.265687 | 2.682573 | -0.002211 |
| 19 | 1 | H | -3.764711 | -2.579994 | -0.087543 |
| 20 | 1 | H | -5.511919 | 1.366859 | -0.228503 |
| 21 | 1 | H | -0.657341 | -1.847076 | -0.830563 |
| 22 | 1 | H | -0.873632 | -2.268699 | 0.8717 |
| 23 | 1 | H | -0.047426 | -0.188639 | 1.65463 |
| 24 | 1 | H | -6.668415 | -0.606743 | -0.357945 |
| 25 | 1 | H | 1.149788 | 0.004583 | -1.188021 |

| | | | | | |
|---|---|---|---|---|---|
| 26 | 1 | H | 2.355098 | -0.205242 | 1.635977 |
| 27 | 1 | H | 3.634938 | 0.036366 | -1.203796 |
| 28 | 1 | H | 4.897677 | -0.176273 | 1.592927 |
| 29 | 1 | H | 7.939768 | -0.002361 | -0.020758 |
| 30 | 1 | H | -2.324677 | 2.896464 | 0.114293 |

| (2*S*)-**2C2** | | | | | |
|---|---|---|---|---|---|
| Center | Atomic | Atomic | Coordinates (Angstroms) | | |
| Number | Number | Element | X | Y | Z |
| 1 | 6 | C | -3.624977 | -1.502515 | -0.080094 |
| 2 | 6 | C | -4.74799 | -0.66881 | -0.188317 |
| 3 | 6 | C | -4.625328 | 0.726635 | -0.164089 |
| 4 | 6 | C | -3.367428 | 1.323018 | -0.027917 |
| 5 | 6 | C | -2.262104 | 0.484052 | 0.074903 |
| 6 | 6 | C | -2.380493 | -0.897562 | 0.055834 |
| 7 | 6 | C | -0.979498 | -1.461136 | 0.147539 |
| 8 | 6 | C | -0.153092 | -0.209151 | 0.575211 |
| 9 | 8 | O | -0.967218 | 0.94695 | 0.205855 |
| 10 | 8 | O | -5.967349 | -1.284004 | -0.315882 |
| 11 | 6 | C | 1.182703 | -0.089433 | -0.08089 |
| 12 | 6 | C | 2.354623 | -0.125123 | 0.577033 |
| 13 | 6 | C | 3.644866 | -0.053417 | -0.080245 |
| 14 | 6 | C | 4.823241 | -0.097463 | 0.573209 |
| 15 | 6 | C | 6.141179 | -0.023742 | -0.077929 |
| 16 | 8 | O | 6.075938 | 0.095653 | -1.433208 |
| 17 | 8 | O | 7.2007 | -0.064003 | 0.517142 |
| 18 | 8 | O | -3.254073 | 2.682826 | 0.001317 |
| 19 | 1 | H | -3.753989 | -2.579326 | -0.101914 |
| 20 | 1 | H | -5.499439 | 1.368354 | -0.241328 |
| 21 | 1 | H | -0.86847 | -2.271298 | 0.874895 |
| 22 | 1 | H | -0.642058 | -1.845488 | -0.824975 |
| 23 | 1 | H | -0.046494 | -0.193619 | 1.668069 |
| 24 | 1 | H | -6.655783 | -0.604753 | -0.38152 |
| 25 | 1 | H | 1.167405 | 0.009185 | -1.166391 |
| 26 | 1 | H | 2.356706 | -0.214292 | 1.663665 |
| 27 | 1 | H | 3.64672 | 0.042235 | -1.164119 |
| 28 | 1 | H | 4.869535 | -0.193121 | 1.65423 |
| 29 | 1 | H | 7.001801 | 0.134655 | -1.735202 |
| 30 | 1 | H | -2.314139 | 2.895973 | 0.127297 |

**Table S5.** Gibbs free energies and Boltzmann populations of all conformers of compound **1** included in the NMR calculationsa.

(2*S**,10*R**,11*R**)-**1A**

| **Conf.** | Structure | G (Hartree) | ΔG (kcal/mol) | Population (%) |
|---|---|---|---|---|
| **1AC1** | 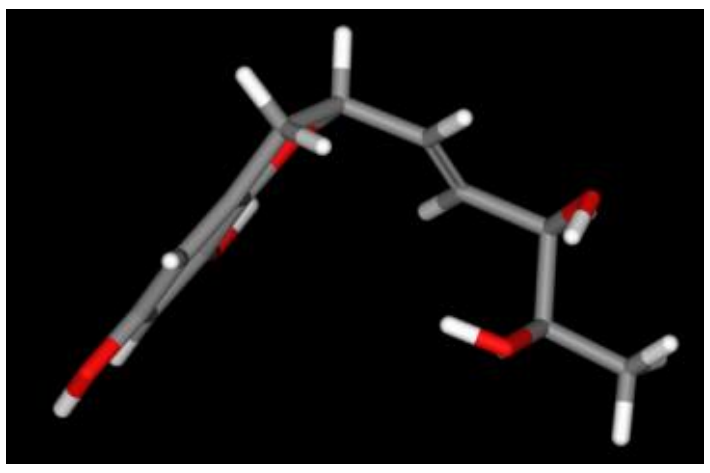 | -881.15677340 | -552934.22 | 83.7 |
| **1AC2** | 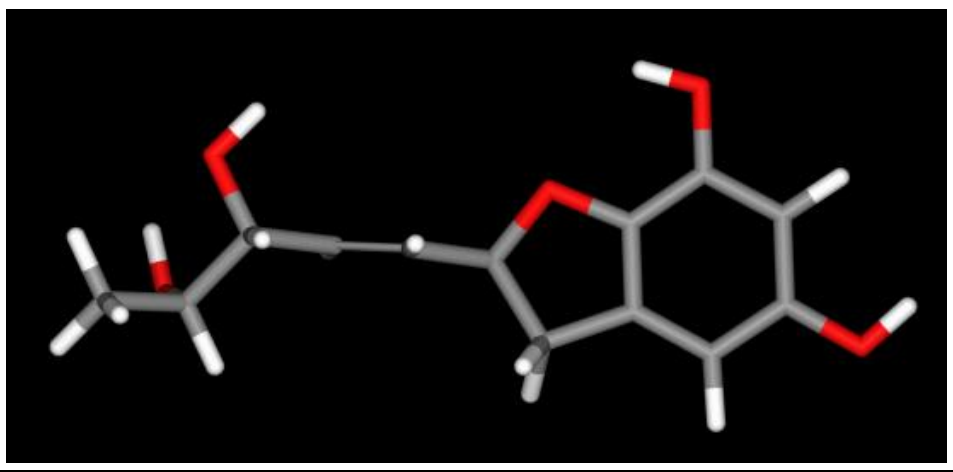 | -881.15522460 | -552933.25 | 16.3 |

2*R**,10*R**,11*R**-**1B**

| **Conf.** | Structure | G (Hartree) | ΔG (kcal/mol) | Population (%) |
|---|---|---|---|---|
| **1BC1** | 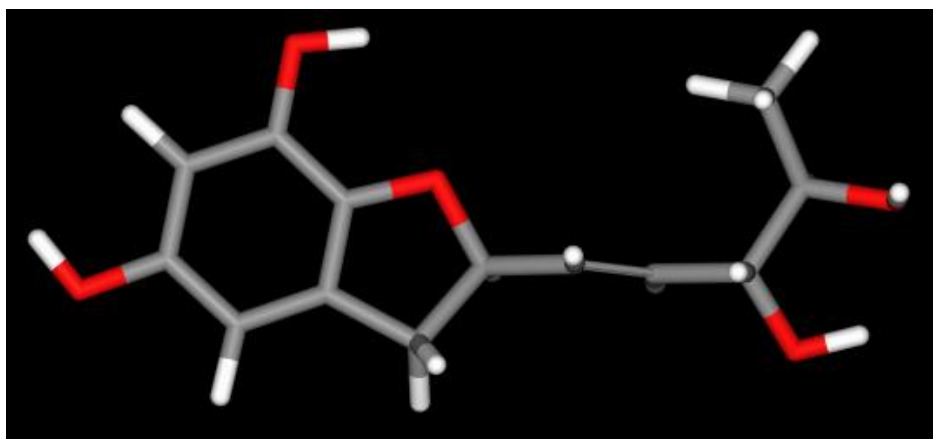 | -881.15597690 | -552933.72 | 42.9 |
| **1BC2** | 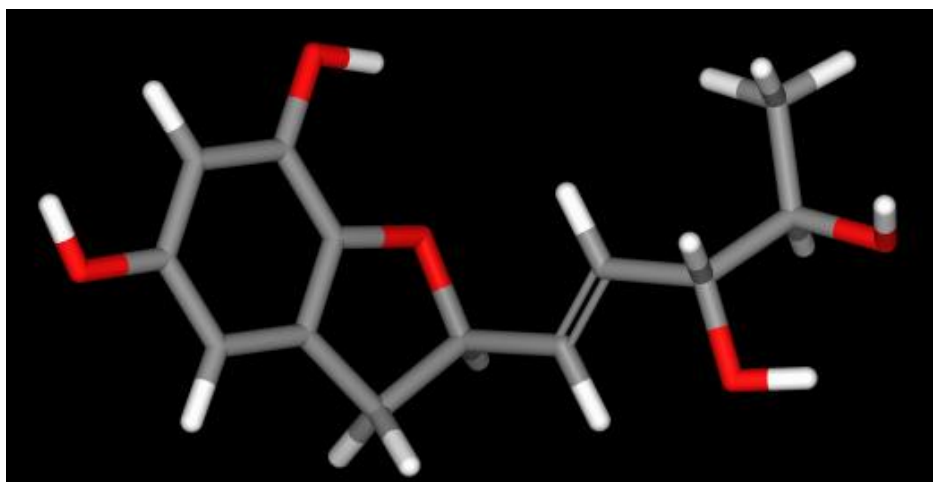 | -881.15568100 | -552933.53 | 31.1 |
| **1BC3** | 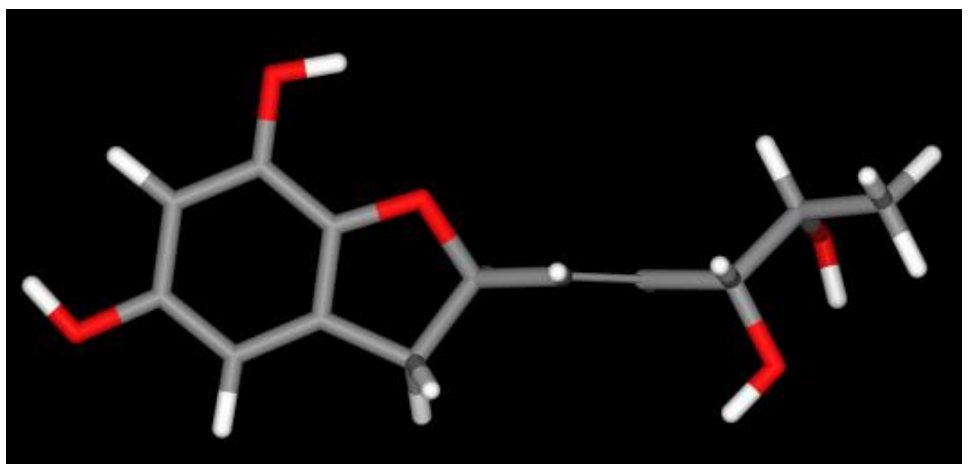 | -881.15507240 | -552933.15 | 16.4 |
| **1BC4** | 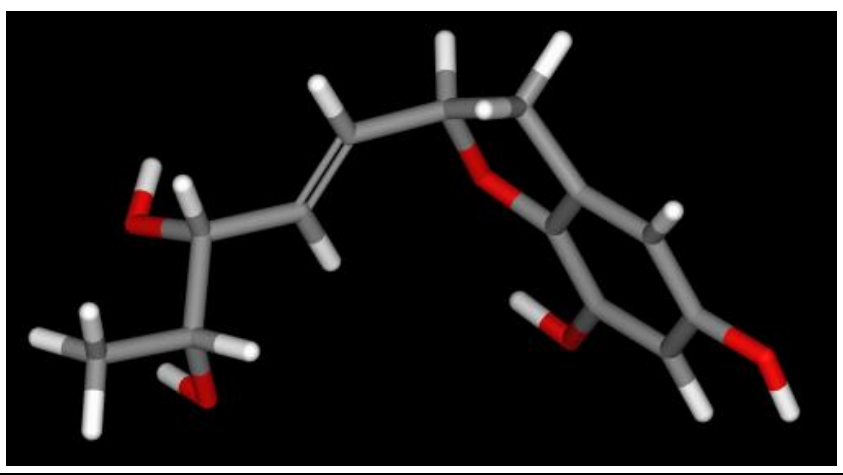 | -881.15455700 | -552932.83 | 9.6 |

| (2*S**,10*S**,11*R**)-**1C** | | | | |
|---|---|---|---|---|
| **Conf.** | Structure | G (Hartree) | ΔG (kcal/mol) | Population (%) |
| **1CC1** | 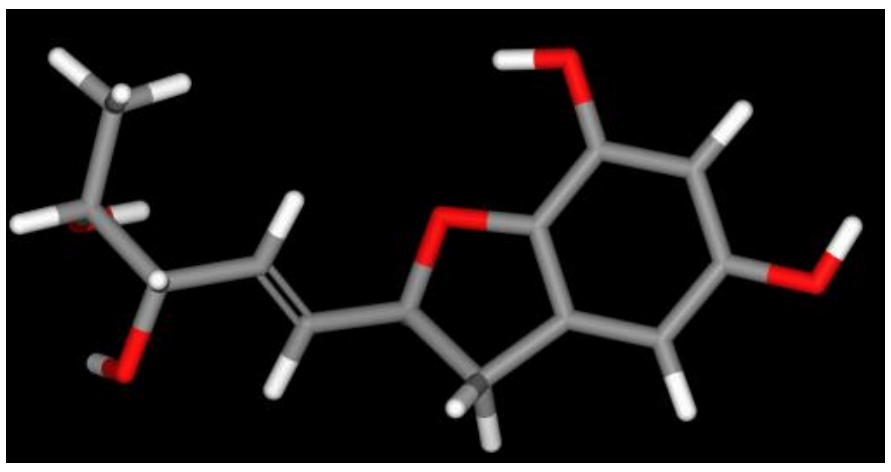 | -881.15562700 | -552933.50 | 51.0 |
| **1CC2** | 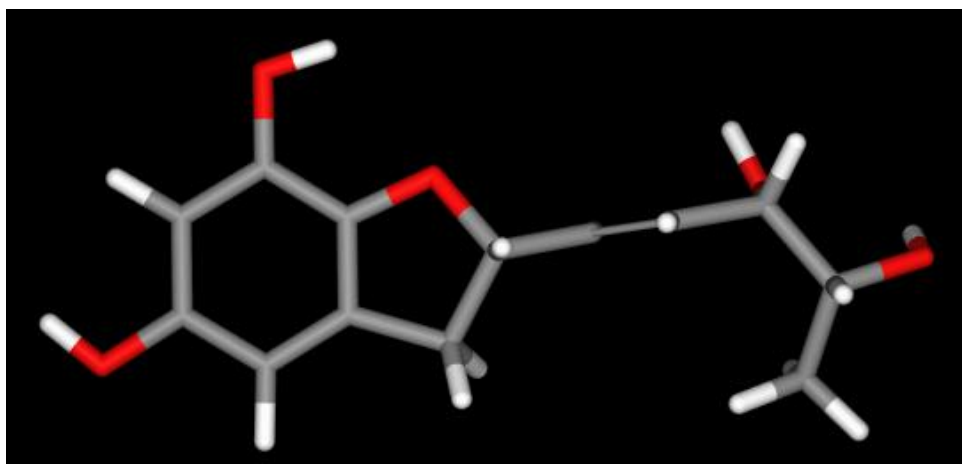 | -881.15506280 | -552933.15 | 28.3 |
| **1CC3** | 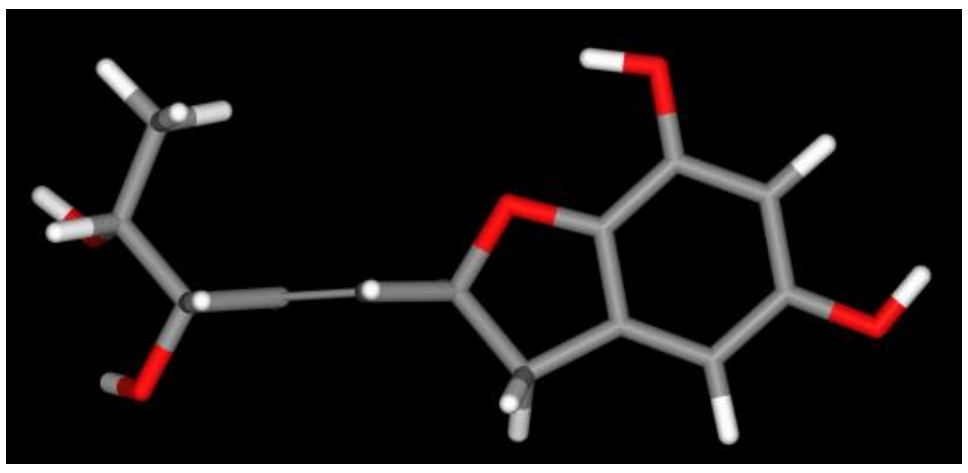 | -881.15430850 | -552932.67 | 12.6 |
| **1CC4** | 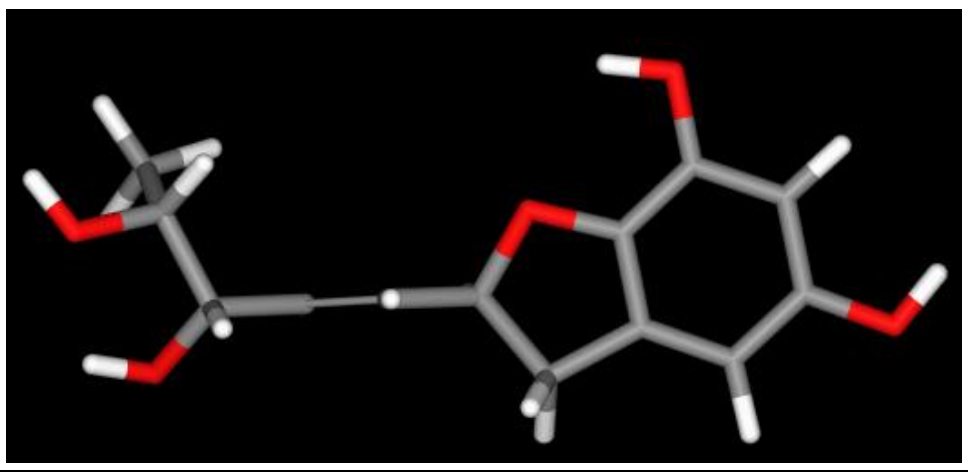 | -881.15389250 | -552932.41 | 8.1 |

| (2*R**,10*S**,11*R**)-**1D** | | | | |
|---|---|---|---|---|
| **Conf.** | Structure | G (Hartree) | ΔG (kcal/mol) | Population (%) |
| **1DC1** | 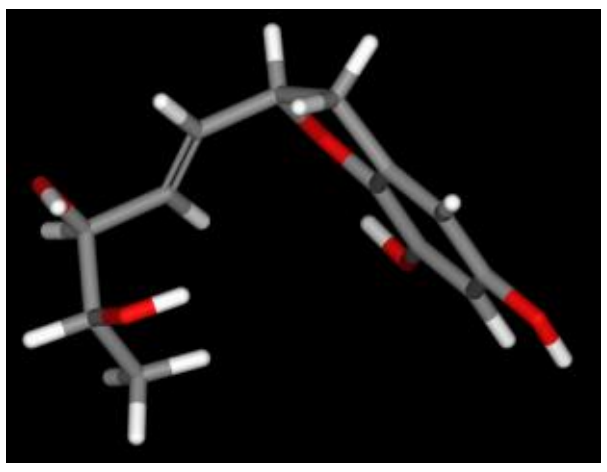 | -881.15610140 | -552933.80 | 80.0 |
| **1DC2** | 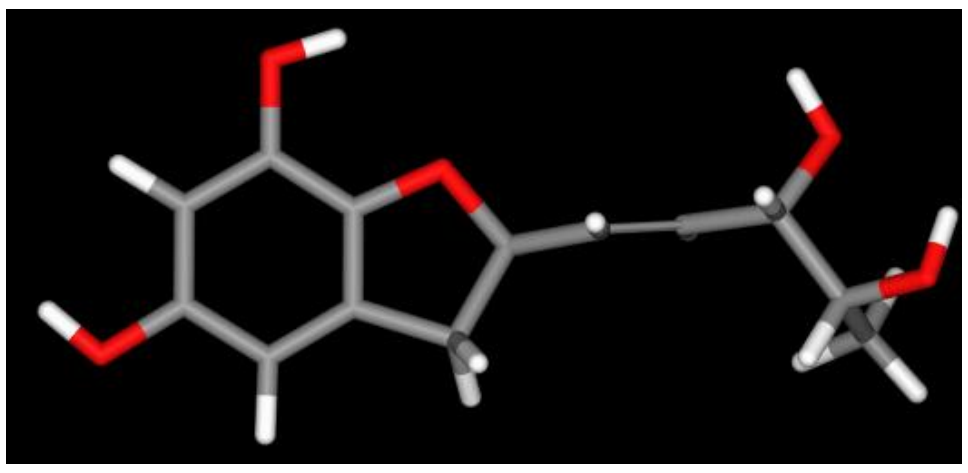 | -881.15448300 | -552932.78 | 14.3 |
| **1DC3** | 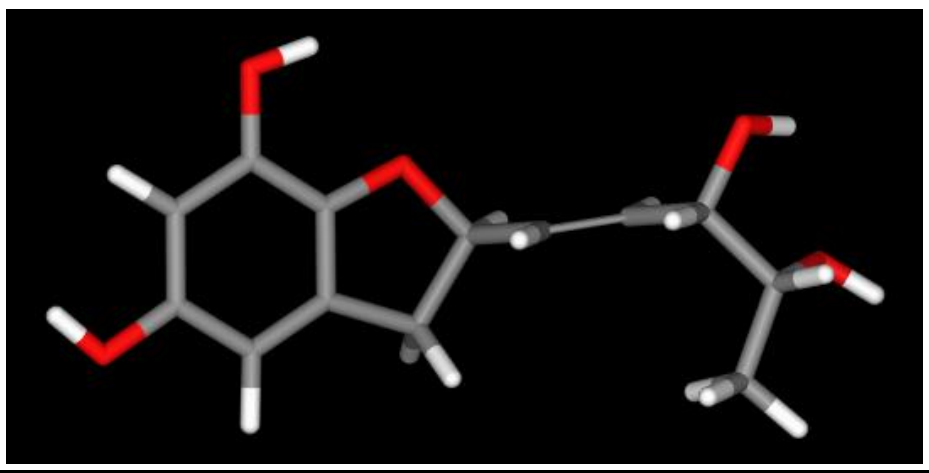 | -881.15359790 | -552932.23 | 5.7 |
